# Molecular Structural, Hydrogen Bonding Interactions, and Chemical Reactivity Studies of Ezetimibe-L-Proline Cocrystal Using Spectroscopic and Quantum Chemical Approach

**DOI:** 10.3389/fchem.2022.848014

**Published:** 2022-02-15

**Authors:** Preeti Prajapati, Jaya Pandey, Poonam Tandon, Kirti Sinha, Manishkumar R. Shimpi

**Affiliations:** ^1^ Department of Physics, University of Lucknow, Lucknow, India; ^2^ Chemistry of Interfaces, Luleå University of Technology, Luleå, Sweden; ^3^ Department of Materials and Environmental Chemistry, Stockholm University, Stockholm, Sweden

**Keywords:** hydrogen bonds, ezetimibe-L-proline, quantum chemical calculations, vibrational spectroscopy, pharmaceutical cocrystal, density functional theory calculations

## Abstract

Ezetimibe (EZT) being an anticholesterol drug is frequently used for the reduction of elevated blood cholesterol levels. With the purpose of improving the physicochemical properties of EZT, in the present study, cocrystals of ezetimibe with L-proline have been studied. Theoretical geometry optimization of EZT-L-proline cocrystal, energies, and structure–activity relationship was carried out at the DFT level of theory using B3LYP functional complemented by 6-311++G(d,p) basis set. To better understand the role of hydrogen bonding, two different models (EZT + L-proline and EZT + 2L-proline) of EZT-L-proline cocrystal were studied. Spectral techniques (FTIR and FT-Raman) combined with quantum chemical methodologies were successfully implemented for the detailed vibrational assignment of fundamental modes. It is a zwitterionic cocrystal hydrogen bonded with the OH group of EZT and the COO^−^ group of L-proline. The existence and strength of hydrogen bonds were examined by a natural bond orbital analysis (NBO) supported by the quantum theory of atoms in molecule (QTAIM). Chemical reactivity was reflected by the HOMO–LUMO analysis. A smaller energy gap in the cocrystal in comparison to API shows that a cocrystal is softer and chemically more reactive. MEPS and Fukui functions revealed the reactive sites of cocrystals. The calculated binding energy of the cocrystal from counterpoise method was −11.44 kcal/mol (EZT + L-proline) and −26.19 kcal/mol (EZT + 2L-proline). The comparative study between EZT-L-proline and EZT suggest that cocrystals can be better used as an alternative to comprehend the effect of hydrogen bonding in biomolecules and enhance the pharmacological properties of active pharmaceutical ingredients (APIs).

## Introduction

The study of an active pharmaceutical ingredient (API) is an essential part of drug discovery and pharmaceutical development. In an approach to develop pharmaceutical compound, improvisation of the physicochemical properties of drugs are needful. Recent research works on cocrystals have drawn much attention toward pharmaceutical applications where properties such as solubility, dissolution rate, bioavailability, hardness (or tableting), and stability of a drug can be improved leaving the chemical properties of pure API unchanged (Chan et al., 2013; Dudenko et al., 2013; Pagire et al., 2013; Sarma and Saikia, 2014; Mekala et al., 2016; Saikia et al., 2016). Cocrystals are crystalline materials composed of multiple components, one being an API and other being a pharmaceutically acceptable coformer connected *via* hydrogen bond, halogen bonds, and π−π interactions (Ali et al., 2012; Pandey et al., 2016).

Ezetimibe (EZT) is a lipid-lowering drug used for the treatment of intestinal resorption of cholesterols and related phytosterols by inhibiting the brush border of the microvilli in the small intestine (Ravikumar and Sridhar, 2005; Brüning et al., 2010; Shimpi et al., 2014). High hydrophobic nature of EZT exhibits low solubility, hence low bioavailability (35–65%) (Shimpi et al., 2014). The current study is an attempt to improve the physicochemical properties of EZT, which is a valuable goal to enhance its therapeutic efficacy. Its cocrystal with L-proline has been studied in which EZT is an API and L-proline is used as a coformer. L-proline is a naturally occurring zwitterion containing an amino group (NH_2_^+^) and a carboxylate group (COO^−^). In order to improve pharmacological properties of the cocrystal (the adduct form due to non-covalent interactions between API and coformer), in particular EZT-L-proline, we have revealed that the EZT-L-proline cocrystal showed improved apparent solubility and solid state stability (Shimpi et al., 2014). Cocrystal solubility is shown to be directly proportional to the solubility of constituent reactants for cocrystals (Alhalaweh et al., 2012). The inclusion of a more water soluble coformer in the cocrystal of a poorly soluble API will typically result in a cocrystal with higher solubility and dissolution rate, compared to the single component crystal of the API. The resulting cocrystal will generate solution levels of the APIs that are supersaturated relative to the poorly soluble crystalline form of the APIs. Rodríguez-Hornedo demonstrated the solubility advantage of pharmaceutical cocrystals using the supersaturation index obtained from eutectic point measurements of cocrystals (Good and Rodríguez-Hornedo, 2009; Shimpi et al., 2018). Moreover, Nangia et al. justified that the enhanced solubility of drug cocrystals is similar to the supersaturation phenomenon of amorphous drugs and also developed the “spring and parachute” concept for amorphous drug dissolution, which is adapted to explain the solubility advantage of pharmaceutical cocrystals (Babu and Nangia, 2011). Cocrystals of EZT-L-proline exist in a monoclinic cell with lattice parameters *a* = 16.54954 Å, *b* = 5.79905 Å, and *c* = 14.03528 Å; α = 90°, β = 104.007°, and γ = 90° in the *P*2_1_ space group. A structural analysis showed that the OH group of EZT is hydrogen bonded with the COO^−^ group of L-proline (Lüdeker and Brunklaus, 2015). Hydrogen bonds between carboxylates and weakly acidic hydroxyl groups in zwitterionic cocrystals are more preferable (Duggirala et al., 2016). However, cocrystals of EZT with imidazole and methyl paraben were also reported earlier (Shimpi et al., 2014; Sugandha et al., 2014).

The density functional theory (DFT) is one of the most reliable computational method for calculating the electronic structure and energies of polyatomic systems (Reva et al., 2003; Dudenko et al., 2013; Srivastava et al., 2018). It is effectively used to solve the problems of material sciences, condensed matter physics, and various other areas. To the best of our knowledge, no theoretical DFT calculations and vibrational analysis on the EZT-L-proline cocrystal have been done so far; therefore, the present study composed of a combination of spectroscopic and computational study supported by DFT. Two different models viz., EZT + L-proline and EZT + 2L-proline were studied theoretically in detail to understand the picture of the hydrogen bond network and their effect in the cocrystals. In order to demonstrate the quantitative and qualitative interpretation of IR and Raman spectra, the calculated vibrational analysis of EZT + L-proline and EZT + 2L-proline were done using potential energy distribution (PED). The calculations were performed at B3LYP level of theory employing 6–311++G(d,p) basis set. Effect and potential of hydrogen bonding was explored by the natural bond orbital analysis (NBO) and quantum theory of atom in molecules (QTAIM). This study also demonstrates the frontier molecular orbital (FMOs) analysis, molecular electrostatic potential map (MEPS), and chemical reactivity descriptors for EZT-L-proline cocrystals with the purpose to explore its molecular properties. Change in chemical properties from API to a cocrystal was examined by chemical reactivity descriptors.

## Experimental Details

Ezetimibe, 1-(4-fluorophenyl)-3-[3-4-(fluorophenyl)-3-hydroxypropyl]-4-(4-hydroxyphenyl)-2 azetidinone, was (purchase from Tecoland; batch number 20120615) generously gifted by Dr. Scott Child (Renovo research, Atlanta, United States). Also, a 7-ml glass vial was charged with 101.7 mg of ezetimibe (0.24 mmol) and 28 mg of L-proline (0.24 mmol). Ethyl acetate:heptane:2,2,2-trifluroethanol (3 ml, 1:3:0.5, vol/vol) was added to make a slurry. The mixture was allowed to dry at room temperature for 72 h to get 110 mg of EZT-L-proline cocrystals.

IR spectral analysis was performed by using a Bruker Vertex 80v FTIR spectrometer in the spectral range 0–4,000 cm^−1^. Spectra of powdered samples were obtained by averaging 128 scans at a resolution of 4 cm^−1^. Raman spectra were collected on a Chromex Sentinel dispersive Raman unit in the region from −250 to 3,750 cm^−1^ equipped with a 785 nm 70 mW excitation laser and a TE-cooled CCD. The data were further collected by SentinelSoft data acquisition software.

## Computational Details

Initially, the electronic structure, optimized geometries, vibrational frequencies, natural bond orbital (NBO) analysis, and molecular electrostatic potential surface (MEPS) of EZT, L-proline, and EZT-L-proline cocrystals were calculated by using density functional theory with B3LYP (Lee et al., 1988; Becke, 1993; Parr and Yang, 1995; Shukla et al., 2017) functional employing 6–311++G(d,p) basis set (Petersson et al., 1988; Petersson and Al-Laham, 1991; Chai and Head-Gordon, 2008; Mendes et al., 2017). All calculations were done with the Gaussian 09 package program (Frisch et al., 2009). Graphical representation of IR and Raman spectra and visualization of all the figures were done using GaussView (Frisch et al., 2000) and Chemcraft (Zhurko and Zhurko, 2005). Vibrational assignment of normal modes were computed on the basis of potential energy distribution (PED) using Gar2ped (Martin and Alsenoy, 1995) with the help of Pulay’s recommendation (Fogarasi et al., 1992). Topological and geometrical parameters at the bond critical point (BCP) were studied within the framework of QTAIM (Bader and Cheeseman, 2000).

## Results and Discussion

### Geometry Optimization and Energies

**FIGURE 1:**
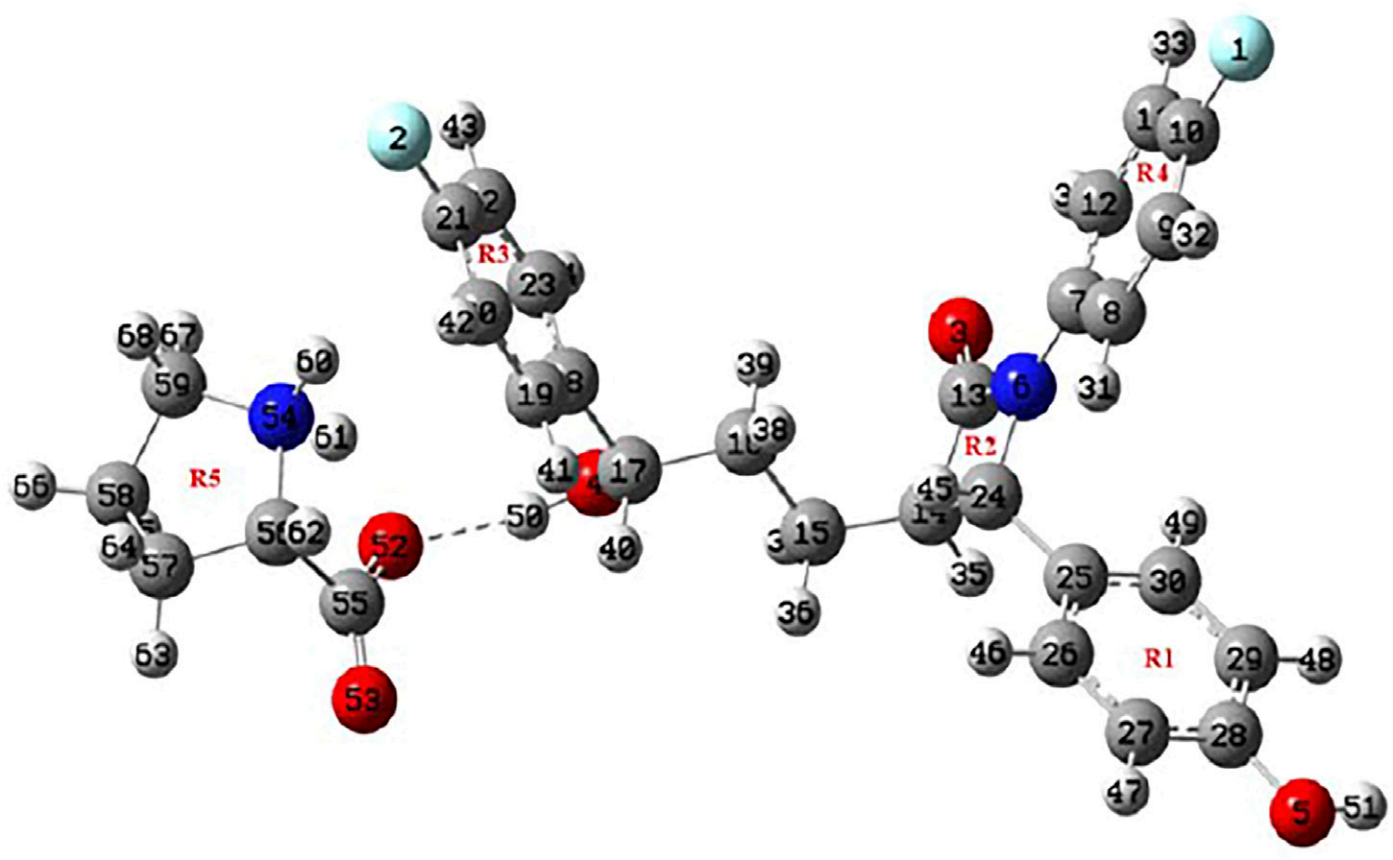
Optimized structure of EZT + L-proline.

**FIGURE 2:**
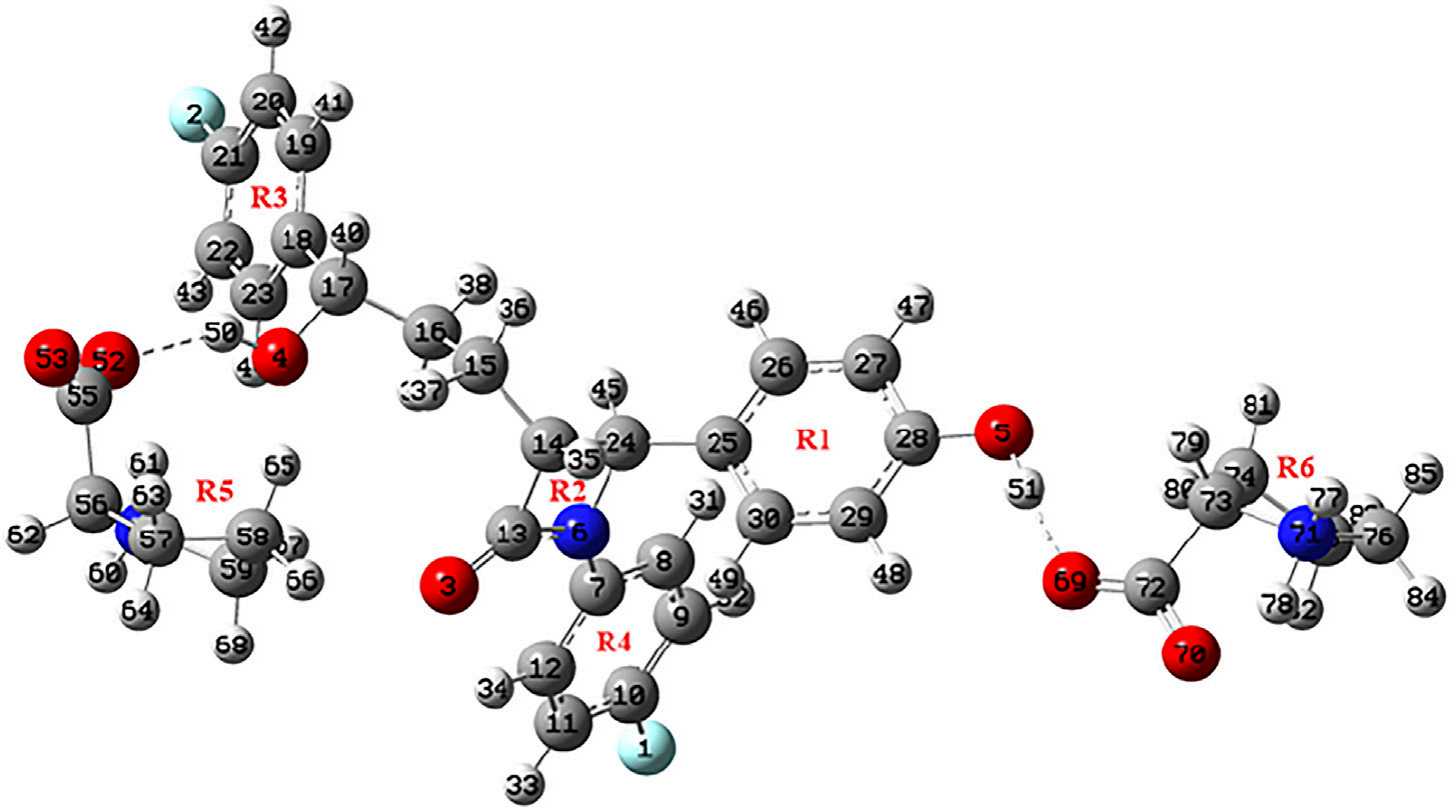
Optimized structure of EZT + 2L-proline.

The crystal structures of EZT and L-proline are already known and both crystallizes in the orthorhombic system with the *P*2_1_2_1_2_1_ space group (Ravikumar and Sridhar, 2005; Brüning et al., 2010; Shimpi et al., 2014). David et al. (Lüdeker and Brunklaus, 2015) reported the crystal structure of EZT-L-proline, which belongs to a monoclinic crystal system with the *P*2_1_ space group. The molecular structures of EZT + L-proline and EZT + 2L-proline were taken from crystallographic data of the EZT-l-proline cocrystal (Lüdeker and Brunklaus, 2015). All the molecules were optimized, and the ground state structures of EZT + L-proline, EZT + 2L-proline, EZT, and L-proline were obtained at B3LYP/6-311++G(d,p) level of theory, as shown in Figures 1, 2, respectively, with the adopted number scheme. Molecular structures of EZT and l-proline are shown in Supplementary Figures S1, S2.

Geometrical parameters (bond lengths, bond angles, and dihedral angles) of EZT, L-proline, and EZT-L-proline cocrystals are tabulated in Supplementary Table S1. A comparison between calculated values of the EZT-L-proline cocrystal and EZT was done, which shows that the calculation is same within 0.009 Å in bond lengths, 0.7° in bond angles, and 8.1° in dihedral angles. However, some variations were observed in the bond lengths of O4-C17 and O4-H50 where the calculated values of EZT/EZT + L-proline were 1.4385/1.4154 and 0.9631/0.9782 Å, respectively. Difference in bond angles around O4-C17-C16 and O4-C17-H40 were also observed. The deviations around these bonds occur in cocrystals due to the presence of hydrogen bonds between the hydrogen atom of an aliphatic hydroxyl group of EZT and the oxygen atom (O52) of L-proline, which are actually not present in APIs.

Also, the comparison of optimized structural parameters (bond lengths, bond angles, and dihedral angles) of EZT + L-proline and EZT + 2L-proline with the experimental values was made, as seen from Supplementary Table S1. Geometrical parameters shows that results of EZT + 2L-proline showed better agreement with the experimental data than those of EZT + L-proline due to the consideration of maximum possible nearest neighboring hydrogen bond interactions. Comparison of experimental and calculated geometrical parameters of EZT and L-proline suggests that the calculation were well capable of replicating the values of crystallographic data. However, some noticeable deviations in experimental and calculated values were found due to the fact that calculation was performed for isolated molecules in the gas phase, whereas the experiment was conducted in the bulk state where all the interactions of crystal packing were involved, as present in the real system.

Ground state optimized energies of EZT + L-proline and EZT + 2L-proline calculated by DFT are −1,808.98 and −2,210.26 Hartrees, respectively. Since the molecules were connected *via* an intermolecular hydrogen bond in EZT-L-proline cocrystals (both the models), their binding energy can be computed as the difference between the total energy of cocrystals (EZT + L-proline and EZT + 2L-proline) and the sum of the individual energies of EZT and L-proline, and the estimated binding energy was −12.82 kcal/mol for EZT + L-proline. Similarly, the binding energy for EZT + 2L-proline was calculated as −27.59 kcal/mol using DFT theory. The calculated binding energy was further corrected for basis set superposition error (BSSE) by using the standard counterpoise method (Boys and Bernardi, 1970; Kobko and Dannenberg, 2001; Ozel et al., 2013), and it was −11.44 kcal/mol (EZT + L-proline) and −26.19 kcal/mol (EZT + 2L-proline).

### Vibrational Analysis

**FIGURE 3:**
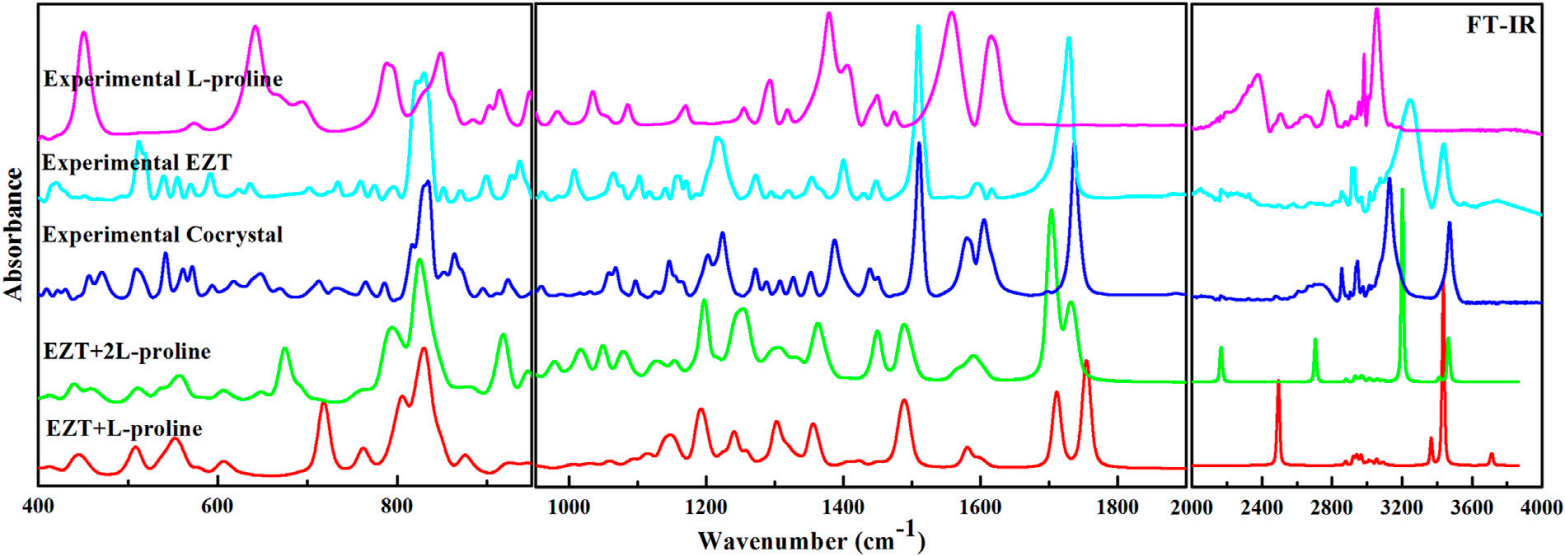
Experimental FTIR spectra of EZT-L-proline, EZT, and L-proline with calculated IR spectra of the cocrystal (EZT + L-proline and EZT + 2L-proline) in the regions 400–1,000, 1,000–2,000, and 2,200–4,000 cm^−1^.

**FIGURE 4:**
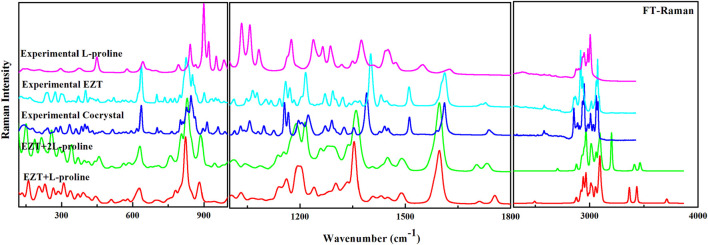
Experimental FT-Raman spectra of EZT-l-proline, EZT, and L-proline with calculated Raman spectra of the cocrystal (EZT + L-proline and EZT + 2L-proline) in the regions 400–1,000, 1,000–2,000, and 2,200–4,000 cm^−1^.

EZT, L-proline, and EZT-L-proline cocrystals (EZT + L-proline/+2L-proline) contain 51, 17, and 68/85 atoms (N), respectively, thus there are 147, 45, and 198/249 (3N-6) normal modes, which are all active in both IR and Raman. Theoretical and experimental vibrational assignment of EZT (API) and L-proline (coformer) are given in Supplementary Tables S2, S3. A detailed assignment of vibrational wavenumbers of EZT-L-proline cocrystal (both the models) is demonstrated in Supplementary Tables S4, S5, respectively. The vibrational spectra of all the molecules were calculated at their optimized geometries employing B3LYP functional with 6-311++G(d,p) basis set. Raman scattering cross section (∂σj/∂Ω), which is proportional to Raman intensities, can be taken from Raman scattering amplitudes, since DFT calculations do not give Raman intensities directly (Runge and Gross, 1984; Polavarapu, 1990; Guirgis et al., 2003). As usual, the calculated wavenumbers were found to be overestimated mainly due to the negligence of the anharmonicity effect and the environment (gas and solid phase). Therefore, the calculated wavenumbers were scaled down by 0.9679 (Qiu et al., 2013) to get a better match of calculated and observed spectra. The comparison of experimental and calculated (scaled) spectra of EZT and L-proline are given in Supplementary Figures S3–S6. Comparison between theoretical and experimental IR and Raman spectra of EZT, L-proline, and EZT-l-proline cocrystals in the region 400–4,000 cm^−1^ and 100–4,000 cm^−1^, respectively, is shown in Figures 3, 4.

Significant changes in bond lengths and stretching wavenumbers were observed due to cocrystal formation between EZT and L-proline, which is presented in Table 1. From the observed spectra (Supplementary Figure S7), it was clearly found that mainly hydroxyl (OH) groups of API and carboxylate (COO^−^) groups of the coformer were involved in cocrystal formation. This was also depicted by Shimpi et al. (2014). In the case of EZT + L-proline, all nearest neighbor interactions were not considered, as a consequence of which discrepancies were found in the higher regions in calculated wavenumbers (Figures 3, 4). To overcome this shortcoming, calculations on EZT + 2L-proline were further done by including the possible neighboring hydrogen bond interaction in the cocrystal. In pure API two OH peaks at 3,434 and 3,254 cm^−1^, respectively, were observed, which were assigned as the stretching vibration mode of O4H and O5H groups, respectively. The difference in wavenumber has occurred due to the fact that both the hydroxyl groups were also involved in hydrogen bond interactions with the neighboring molecules; as it can be seen from Supplementary Figure S8, the O5H group was interacting with one molecule and the O4H group was interacting with two of its nearest molecules that was not theoretically predicted because the calculation was performed on a single isolated molecule in which the nearest neighbor interactions were not taken into account. An experimental spectral analysis of API (EZT) and the cocrystals (EZT + L-proline and EZT + 2L-proline) confirms the presence of two hydroxyl peaks in the IR spectrum. The stretching vibration mode of O5H was calculated at 3,710 cm^−1^ in EZT and at 3,711 cm^−1^ in EZT + L-proline, which was assigned at 3,465 cm^−1^ in EZT + 2L-proline corresponding to IR peak at 3,471 cm^−1^ (cocrystal). Another hydroxyl (O4H) peak was calculated at 3,700/3434/3,202 cm^−1^ in EZT/EZT + L-proline/EZT + 2L-proline, and this OH peak was recorded at 3,128 cm^−1^. From Figure 3 and also from Table 1 it was remarked that large deviation in calculated wavenumber of O5H was observed because nearest neighbor interactions were not considered, and thus this OH group is free in case of EZT + L-proline, but in case of EZT + 2L-proline, a better agreement between experimental and theoretical values was obtained due to the incorporation of hydrogen bond interactions (Supplementary Figures S9, S10). Downshift in the wavenumbers and shifting of bond lengths of both the OH groups were observed when a comparison between the API and the cocrystal was done. Since the O4H group was involved in hydrogen bond formation with the carboxylate group of L-proline in both EZT + L-proline and EZT + 2L-proline, decrement in wavenumber and shifting in the bond length occurred that can be clearly seen from Table 1. Elongation in the bond length of the O5H group by 0.0261 Å has occurred due to the intermolecular hydrogen bonding of OH and COO^−^ groups in EZT + 2L-proline, while no such remarkable changes were noticed in EZT + L-proline.

**TABLE 1:** Comparison of experimental and theoretical stretching frequency (cm^−1^) and bond length (Å) involved in hydrogen bonding.

O-H group			C=O group				NH_2_^+^ group			COO^−^ group		
Molecules	Bond length (Å)	Stretching frequency (cm^−1^)		Bond length (Å)	Stretching frequency (cm^−1^)		Bond length (Å)	Stretching frequency (cm^−1^)		Bond length (Å)	Stretching frequency (cm^−1^)	
		IR	Raman		IR	Raman		IR	Raman		IR	Raman
Experimental												
EZT	0.8199	3,434	—	1.2298	1728	1729	—	—	—	—	—	—
	0.8199	3,254										
L-proline	—	—	—	—	—	—	0.9981 (N54H60)	3183 (asym)	—	1.2576	1613	1625
							0.9841 (N54H61)	3134 (sym)		1.2751	
EZT-L-proline	0.9922	3,471	—	1.1953	1736	1727	1.0134 (N54H60)	3075 (asym)	3077	1.2105	1605	1611
	1.0396	3,128					1.0134 (N54H61)	2741 (sym)	2576	1.2103		
Theoretical												
EZT	0.9633	3,710	3,710	1.2068	1758	1758	—	—	—	—	—	—
	0.9631	3,700	3,700									
L-proline	—	—	—	—	—	—	0.9981(N54H60)	3391(asym)	3391(asym)	1.2273	1729	1729
							1.0172(N54H61)	3322(sym)	3322(sym)	1.2632		
EZT + L-proline	0.9630	3,711	3711	1.2074	1755	1755	1.0198(N54H60)	3366(asym)	3366(asym)	1.2216	1712	1712
	0.9782	3,434	3434				1.0846(N54H61)	2492(sym)	2492(sym)	1.2803		
EZT+2L-proline	0.9766, 0.9894	3,465, 3,202	3,465, 3,202	1.2134	1733	1733	1.0162(N71H77)	3416(R6-asym)	3416(R6-asym)	1.2237	1706(R5) 1704(R6)	1706(R5) 1704(R6)
							1.1229(N71H78)	3411(R5-asym)	3411(R5-asym)	1.2740		
							1.0166(N54H60)	2705(R5-sym)	2705(R5-sym)	1.2353		
							1.0672(N54H61)	2166(R6-sym)	2166(R6-sym)	1.2645		

The COO^−^ group of L-proline generates a peak at 1,613 cm^−1^ in IR and at 1,625 cm^−1^ in Raman spectra, which was theoretically assigned as asymmetrical stretching vibrational mode of the carboxylate group at 1,729 cm^−1^. Moving from the coformer to the cocrystal, the stretching mode of the same group was calculated at 1,712 cm^−1^ (EZT + L-proline) and 1,706 cm^−1^ (EZT + 2L-proline) for the observed peak at 1,605 cm^−1^, with a downshift of wavenumber followed by a decrement of bond length of *C* = O52 by 0.0171 Å and *C* = O69 by 0.0108 Å clearly indicating that this particular group of L-proline was hydrogen bonded with the OH groups of EZT to form the cocrystal. From Table 1, appreciable changes were observed both in API (OH groups) and coformer (COO^−^ group) as expected. The lowering of wavenumber in OH groups of API with elongation of bond length and decrement of wavenumber in the COO^−^ group of the coformer with a decreased bond length confirms that both the groups are equally involved in the formation of cocrystals *via* O4-H50•••O52-C55 and O5-H51•••O69-C72 hydrogen bond. Changes were also suspected in the NH_2_^+^ group of L-proline due to the presence of intramolecular hydrogen bond between C55-O52•••H61-N54 (EZT + L-proline and EZT + 2L-proline) and C72-O69•••H78-N71 (EZT + 2L-proline).

A very unusual change in the bond length of N54-H60 and N54-H61 was noticed; in pure L-proline, there was a difference of 0.014 Å in the bond length, but in case of the cocrystal no such difference was found in the NH_2_^+^ group of L-proline. The reason behind this might be the presence of a strong intramolecular interaction between the O52•••H61-N54 group, Supplementary Figure S9, S10, whereas, in case of the cocrystal, the carbonyl group (C55 = O52) of L-proline was already making a hydrogen bond with the O4H group of EZT due to which the intramolecular interaction among O52•••H61-N54 was weakened, and hence the bond length of N54-H60 and N54-H61 remain unchanged. This asymmetrical stretching mode of the NH_2_^+^ group was assigned at 3,391/3,416 cm^−1^ in coformer/cocrystal reflecting increment in the bond length by 0.1,057 Å. The symmetrical stretching mode was calculated at 3,322 cm^−1^ and 2,705 cm^−1^ in L-proline and EZT + 2L-proline, respectively. The spectral analysis also showed that L-proline exists as a zwitterion because no OH peak corresponding to L-proline was observed in the coformer or in the cocrystal.

### Natural Bond Orbital Analysis

The natural bond orbital (NBO) analysis was originated to examine the effect of covalency and hybridization in a molecular system. H-bond and other strong bound van der Waal interactions were first examined by Foster and Weinhold (1980) and further extended by Reed et al.(1988). It is very efficient method for studying inter- and intramolecular bonding and also enables a reliable way to examine the hyperconjugative interactions and charge transfer in a molecular system (Chandran et al., 2012). Charge transfer from filled donor orbitals to unfilled acceptor orbitals are strengthened by the second order perturbation energy (E2) value. A higher value of E2 reflects more intensive interaction among them (Khan et al., 2017).

The most important interactions between donor and acceptor orbitals for the cocrystal are listed in Supplementary Table S6. It is seen from the Table that in the cocrystal (EZT + 2L-proline), charge transfer takes place from EZT (unit 1) to L-proline (unit 2) due to transition of LP(1) O4→ σ* (N54-H61) with a stabilization energy of 0.39 kcal/mol and confirms the presence of a classical hydrogen bond N54-H61•••O4. Another interaction of σ (O5-H51) → σ * (O69-C72) stabilizes the molecule to 0.17 kcal/mol from unit 1 to 3, leading to the formation of O5-H51•••O69 bond, which is also responsible for the formation of the cocrystal between the API and the coformer. A strong charge transfer took place from LP(1)O52/LP(3)O52 → σ* (O4-H50) (from unit 2 to unit 1) and from LP(1)O69/LP (2) O69 → σ* (O5-H51) (unit 3 to unit 1), which confirms the presence of a classical intermolecular interaction O4-H50•••O52 and O5-H51•••O69 that stabilizes the molecular system up to 5.21/4.62 kcal/mol and 7.87/18.65 kcal/mol.

A very strong electron transition between lone pair of nitrogen (N6) and antibonding π* (O3-C13)/π* (C7-C8) was noticed, which stabilizes the API (EZT) with a maximum energy of 60.01/35.81 kcal/mol. Similarly, charge transfer between LP (3) O52 → π* (O53-C55) stabilizes the coformer (L-proline) with a stabilization energy of 68.49 kcal/mol.

### Atom in Molecules Calculations

QTAIM methodology discovered by Bader et al. (1983), Bader and MacDougall (1985), and Bader (1990) was applied to get a deeper insight about H-bond interactions in a molecular system. The bond critical point (BCP) indicates the accumulation of electron density and can be used to study the chemical bonds and their character. Geometrical and topological parameters are useful quantities to explain the nature and strength of the H-bond. Existence of H-bonds was given by Koch and Popelier (1995). Strength and nature of H-bonds were characterized by Rozas et al. (2000), which were as follows: 1) (∇^2^ρ_BCP_) <0 and HBCP <0 for strong H-bond and covalent in nature, 2) (∇^2^ρ_BCP_) >0 and HBCP <0 for medium H-bond and partially covalent in nature, and 3) (∇^2^ρ_BCP_) >0 and HBCP > 0 for weak H-bond and electrostatic in nature.

**FIGURE 5:**
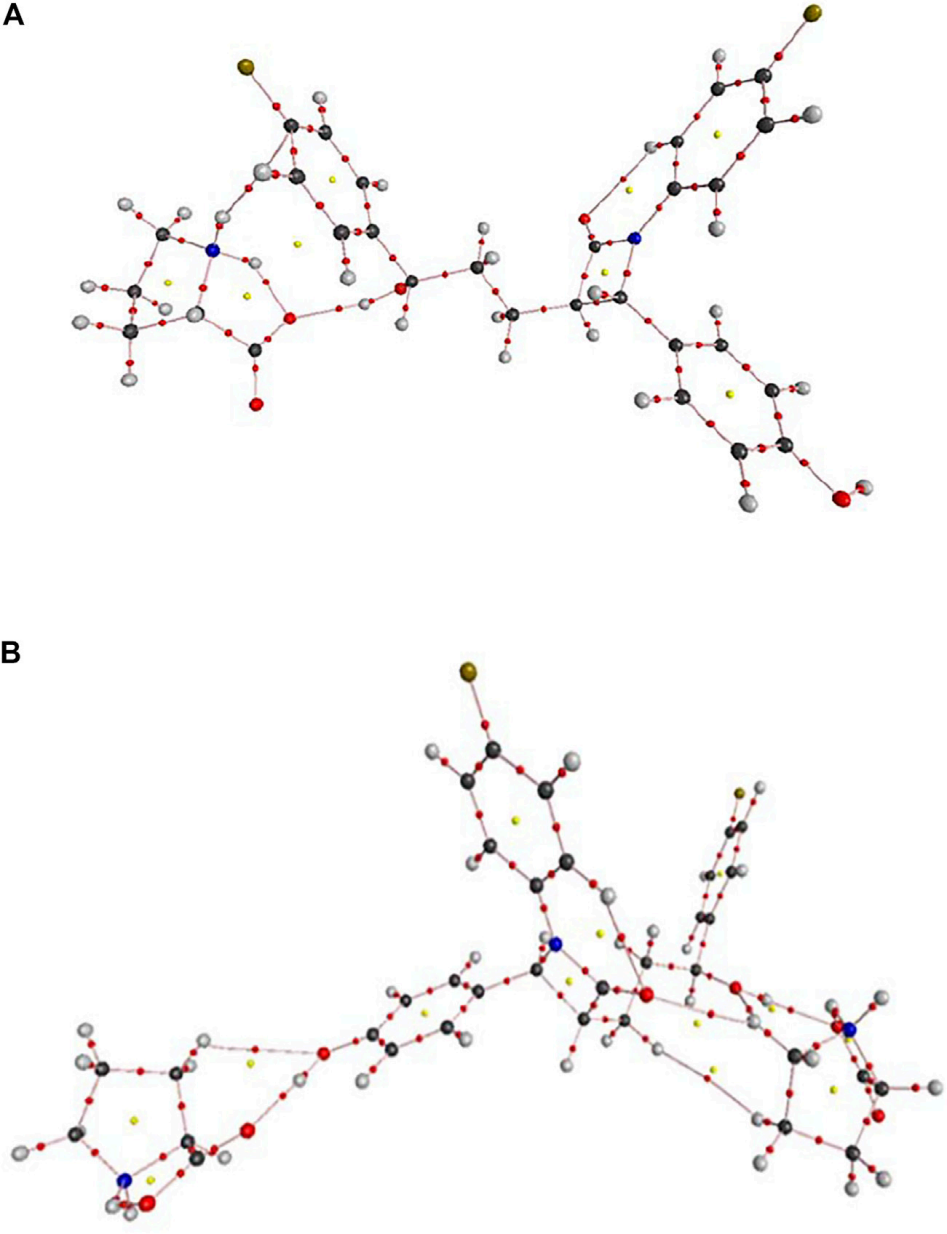
**(A)** Molecular graph of the EZT + L-proline cocrystal. Bond critical points (small red spheres), ring critical points (small yellow sphere), and bond paths (pink lines) were calculated using B3LYP/6-311++G(d,p). **(B)** Molecular graph of the EZT + 2L-proline cocrystal. Bond critical points (small red spheres), ring critical points (small yellow sphere), and bond paths (pink lines) were calculated using B3LYP/6-311++G(d,p).

The molecular graph of EZT-L-proline (EZT + L-proline and EZT + 2L-proline) using AIM program at the B3LYP/6-311++G(d,p) level is drawn in Figures 5A,B, respectively. Calculated topological and geometrical parameters along with energies of the interacting atoms are provided in Table 2 (EZT + L-proline) and Table 3 (EZT + 2L-proline). Various kinds of interactions in the cocrystal (EZT + 2L-proline) were found including inter- and intramolecular H-bonds. The bond length of H50•••O52 and H51•••O69 interactions between the API and the coformer was smaller, therefore these forms strong intermolecular H-bonds. Another strong intramolecular H-bond (H61•••O52) was observed in L-proline between the amino group (NH_2_^+^) and the carboxylate group (COO^−^). On the basis of criteria set by Rozas et al., the strength of these hydrogen bonds is medium and also partially covalent in nature, as (∇^2^ρ_BCP_) > 0, HBCP < 0. According to the strength of the H-bonds, they are arranged in the following order: O70•••H78 > H50•••O52 > H51•••O69 > H61•••O52 > H34•••O3 > H67•••O3 > H67•••O4 > O5•••H80.

**TABLE 2:** Geometrical parameters (bond length) and topological parameters of bonds of interacting atoms in the cocrystal (EZT + L-proline): electron density (ρ_BCP_), Laplacian of electron density (▽^2^ρ_BCP_), electron kinetic energy density (G_BCP_), electron potential energy density (V_BCP_), bond ellipticity (ε), total electron energy density (H_BCP_) at bond critical point (BCP), and estimated hydrogen bond energy (E_int_).

Interaction	Bond length (Å)	ρ_BCP_	G_BCP_	V_BCP_	▽^2^ρ_BCP_	H_BCP_	ε	E_int_ (kcal/mol)
H61•••O52	1.5792	0.0704	0.0173	−0.0722	0.1505	−0.0549	0.0204	−22.6527
C21•••H60	2.6918	0.0072	−0.0094	−0.0032	0.0204	−0.0126	1.7082	−1.0040
H50•••O52	1.8249	0.0316	−0.0010	−0.0267	0.1152	−0.0277	0.0617	−8.3771
H34•••O3	2.4238	0.0116	−0.0012	−0.0073	0.0389	−0.0085	0.1566	−2.2903

**TABLE 3:** Geometrical parameters (bond length) and topological parameters of bonds of interacting atoms in the cocrystal (EZT+2L-proline): electron density (ρ_BCP_), Laplacian of electron density (▽^2^ρ_BCP_), electron kinetic energy density (G_BCP_), electron potential energy density (V_BCP_), bond ellipticity (ε), total electron energy density (H_BCP_) at bond critical point (BCP), and estimated hydrogen bond energy (E_int_).

Interactions	Bond length (Å)	ρ_BCP_	G_BCP_	V_BCP_	▽^2^ρ_BCP_	H_BCP_	ε	E_int_ (kcal/mol)
H50•••O52	1.6666	0.0576	0.0097	−0.0550	0.1457	−0.0452	0.0339	−17.2437
H61•••O52	1.8371	0.0305	−0.0016	−0.0262	0.1784	−0.0278	0.0067	−8.2266
H34•••O3	2.4288	0.0116	−0.0012	−0.0073	0.0388	−0.0085	0.0157	−2.2747
H67•••O4	2.7945	0.0051	−0.0008	−0.0034	0.0020	−0.0042	0.0841	−1.0636
H67•••O3	2.5054	0.0083	−0.0010	−0.0048	0.0272	−0.0058	0.0293	−1.4966
H51•••O69	1.6919	0.0451	0.00384	−0.4219	0.1380	−0.4180	0.0326	−3.2402
O5•••H80	2.7696	0.0051	−0.0008	−0.0031	0.0189	−0.0039	0.6036	−0.9789
O70•••H78	1.4717	0.0919	0.03361	−0.1005	0.1332	−0.0336	0.0145	−31.5412

The bond ellipticity measures the extent of charge accumulation (Koch and Popelier, 1995; Seliger et al., 2010). It gives the measure of π character and the structural stability of a bond. A higher value of ε in O5•••H80, H67•••O3, H67•••O4, and H67•••C21 confirms that these bonds are comparatively weaker than other bonds. Interaction energy of the cocrystal (EZT + 2L-proline) calculated on the basis of AIM theory is the energy of O4-H50•••O52 and O5-H51•••O69 bonds which is −30.48 kcal/mol.

### Chemical Reactivity

#### Molecular Electrostatic Potential Surface

The molecular electrostatic potential surface (MEPS) is an effective tool in identifying prospective sites involved in electrophilic/nucleophilic attacks as well as their relative reactivity. It is frequently used to understand the localization of electron density and the nature of interactions between the molecules through pictorial representation with a colored spectrum; red represents the region of negative electrostatic potential, blue represents the region of most positive electrostatic potential, and green represents the region of zero potential (Prajapati et al., 2016).

**FIGURE 6:**
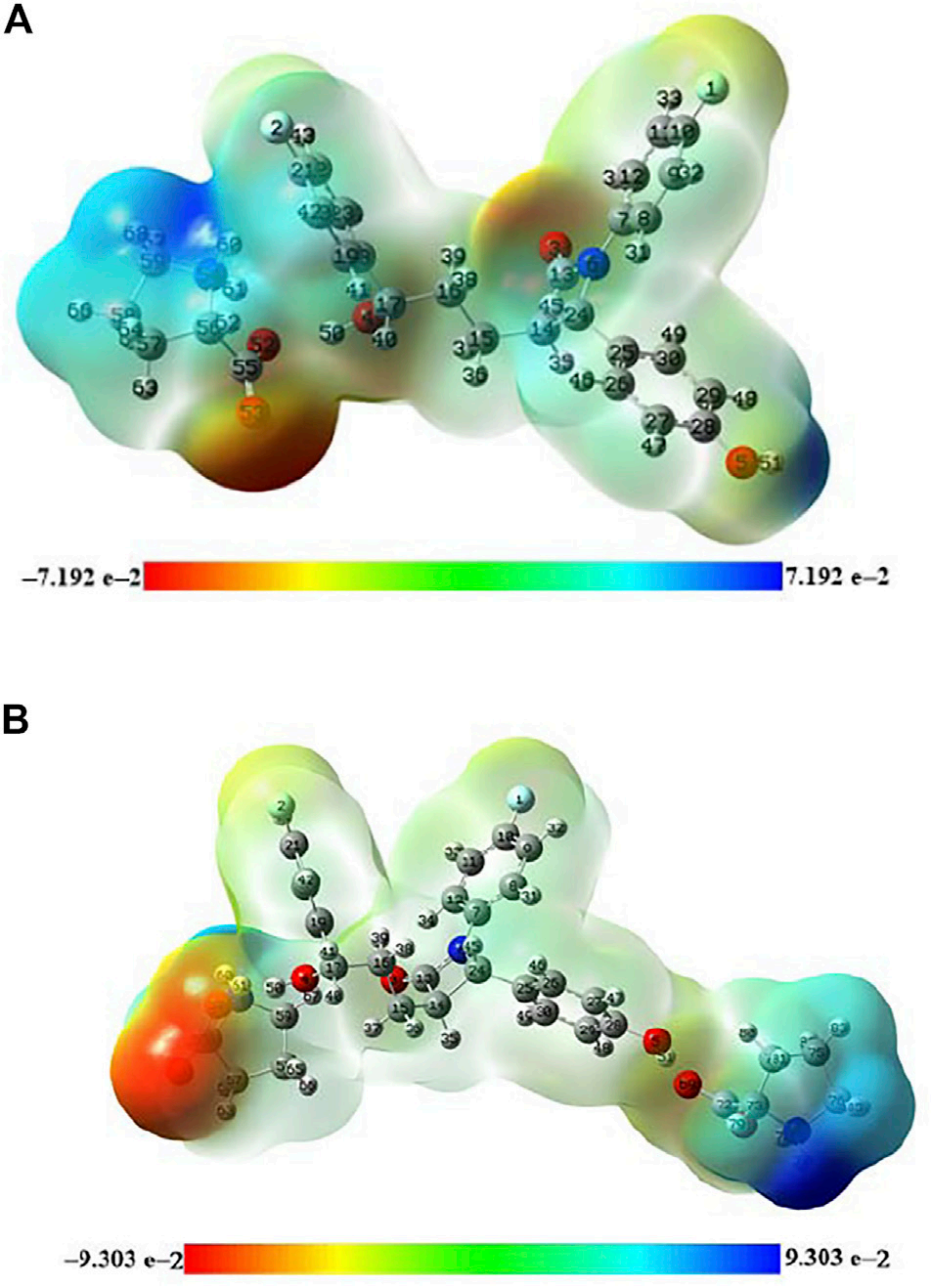
**(A)** Molecular electrostatic potential surface (MEPS) of EZT + L-proline formed by mapping total density over electrostatic potential in the gas phase. **(B)** Molecular electrostatic potential surface (MEP) of EZT + 2L-proline formed by mapping total density over electrostatic potential in the gas phase.

The MEPS of EZT, L-proline, EZT + L-proline, and EZT + 2L-proline are presented in Supplementary Figures S11, S12, Figures 6A,B, respectively. According to the calculated MEPS map, the negative potential was mainly over the carbonyl group in both the API (EZT) and the coformer (L-proline), reflecting it as a center of electrophilic attack. On the other hand, the blue shade over both aromatic and aliphatic OH groups of EZT and the NH_2_^+^ group of L-proline denotes the center of nucleophilic attack. An interesting fact came out that electrostatic potential around the OH (O5-H51) group of EZT and the COO^−^ (O69=C72) group of the EZT-L-proline cocrystal reduces due to the formation of hydrogen bonds between them. In this way, the MEPS map can be used to predict the reactive sites and hence the possible hydrogen bonding in a molecular system.

#### Molar Refractivity

The MR value of a pharmaceutical compound is an important parameter for the determination of biological activity. It reflects the dispersivity of the valence electrons, which depends on the mass, charge, and polarizability of the molecule. MR is defined by the Lorenz–Lorentz equation (Padron et al., 2002; Verma and Hansch, 2005). The MR value for EZT and EZT + L-proline/EZT + 2L-proline are calculated as 68.89 (Prajapati et al., 2016) and 79.54/91.01 e.s.u, respectively. An appreciable result was observed from the theoretical values that the MR values have increased from the API (EZT) to the cocrystal (EZT-L-proline cocrystal), which confirms the reactivity of cocrystal in comparison to APIs.

#### Frontier Molecular Orbital Analysis

**FIGURE 7:**
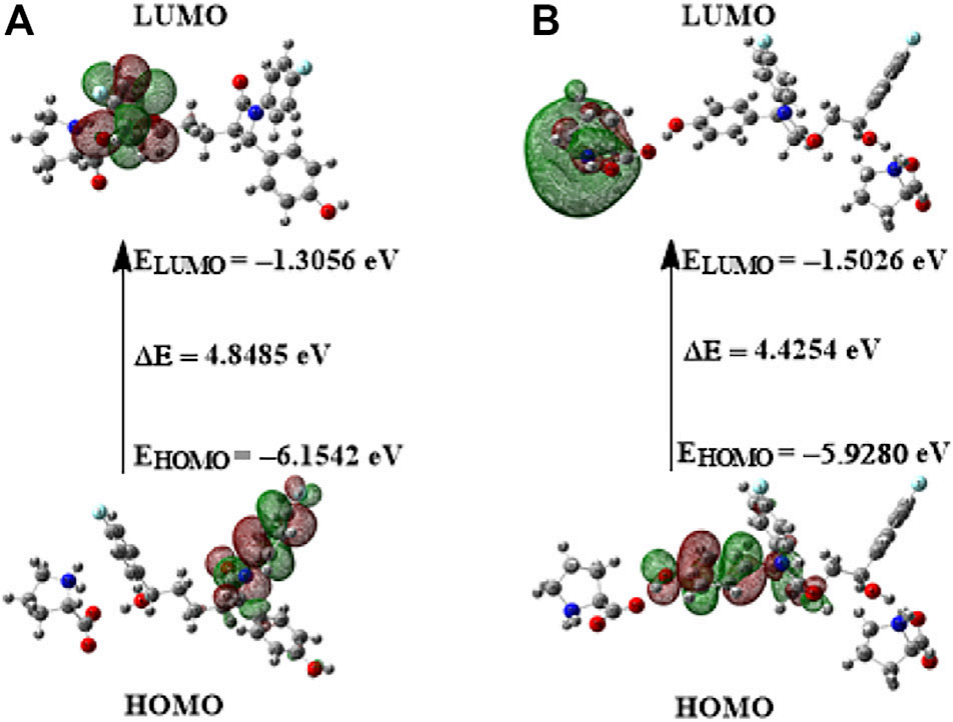
HOMO–LUMO plot of the EZT-L-proline cocrystal with orbitals involved in electronic transitions: **(A)** EZT + L-proline and **(B)** EZT + 2L-proline.

Chemical stability of the molecule is greatly influenced by transitions between the highest occupied molecular orbital (HOMO) and the lowest unoccupied molecular orbital (LUMO). These are the frontier molecular orbitals, and the energy difference between HOMO and LUMO is of great importance (Padron et al., 2002). In an attempt to determine the chemical reactivity of the molecular system, HOMO and LUMO energies and the energy gap (E_LUMO_–E_HOMO_) were calculated using B3LYP/6-311++G(d,p) basis set for both the API and the cocrystal. The molecular orbitals plot of frontier orbitals of EZT and the cocrystal (EZT + L-proline and EZT + 2L-proline) is pictorially represented in Supplementary Figure S13 and Figure 7, respectively. Chemical stability of a molecular system is estimated by its HOMO–LUMO energy gap. The calculated HOMO–LUMO energy gap of EZT and EZT-L-proline/EZT + 2L-proline are 5.2979 eV, 4.8485/4.4254 eV, respectively. A small energy gap between molecular orbitals ensures low stability and hence high reactivity of the system. A larger energy gap implies low reactivity and high stability of the molecule (Srivastava et al., 2016). The energy gap decreases after the formation of the cocrystal (EZT + L-proline and EZT + 2L-proline). It is clearly seen from the HOMO and LUMO energies of EZT and the ezetimibe-L-proline cocrystal (in both the models) that with the addition of L-proline (conformer) to the EZT the energy gap between HOMO and LUMO decreases in the ezetimibe-L-proline cocrystal. This reduction of energy gap shows that the cocrystal is chemically more reactive than the API. In case of the EZT-L-proline cocrystal (both the models), charge is mostly localized on EZT in HOMO, while charge transfers from EZT to L-proline in LUMO due to electronic transitions.

#### Global Reactivity Descriptors

Different types of global reactivity descriptors, including, electronegativity (*χ*), chemical potential (*µ*), global hardness (*η*), global electrophilicity index (*ω*), and global softness (*S*) are found to be computationally effective in predicting the reactivity features of a molecular system. Global reactivity descriptors were calculated on the basis of Koopman’s theorem using energies of frontier molecular orbitals E_HOMO_ and E_LUMO_, as follows (Pearson, 1963; Parr and Yang, 1989):

$$\mathrm{\chi =}-\frac{1}{2}\left(E_{\mathrm{HOmo}}+E_{\mathrm{LUMO}}\right),$$

$$\mathrm{\mu =}-\mathrm{\chi =}-\frac{1}{2}\left(E_{\mathrm{HOmo}}+E_{\mathrm{LUMO}}\right),$$

$$\eta =\frac{1}{2}\left(E_{\mathrm{LUMO}}+E_{\mathrm{HOMO}}\right),$$

$$\omega =\frac{\mu^{2}}{2\eta },$$

$$S=\frac{1}{2\eta }.$$

Energies of frontier molecular orbitals, energy gap, electronegativity (*χ*), chemical potential (*μ*), hardness (*η*), softness (*S*), and electrophilicity index (*ω*) for the API, coformer, and cocrystal (EZT + L-proline and EZT + 2L-proline) were calculated (Pearson, 1989; Parr and Pearson, 1983; Geerlings et al., 2003; Parr et al., 1999; Chattaraj et al., 2006; Padmanabhan et al., 2007) and are tabulated in Table 4. Softness of the molecule can be correlated to the reactivity and hence to the stability of the system. High reactivity suggests that the molecule is softer (polarizable) and is less stable (Srivastava et al., 2016). The value of global hardness (*η*) was higher in the API (2.6489 eV) and lower in the cocrystal (2.4243/2.2127 eV in EZT + L-proline/EZT + 2L-proline). Similarly, the higher value of global softness (*S*) in the cocrystal (0.2062/2.2259 eV) and the lower value in the API (0.1888 eV) confirms high chemical reactivity of the cocrystal (EZT + L-proline/EZT + 2L-proline) in comparison of API (EZT). Furthermore, the calculated high value of *ω* reflects that the cocrystal behaves as a strong electrophile. Overall results suggest that chemical properties alters from the API to the cocrystal, which also shows that the cocrystal is chemically more reactive than the API and may also be used as a better alternative for the improvisation of physicochemical properties of APIs.

**TABLE 4:** Calculated E_LUMO_, E_HOMO_, energy gap (E_LUMO_–E_HOMO_), chemical potential (*μ*), electronegativity (*χ*), global hardness (*η*), global softness (*S*), and global electrophilicity index (*ω*) for EZT, L-proline, and EZT-l-proline (EZT + L-proline and EZT + 2L-proline).

Reagent	E_LUMO_ (eV)	E_HOMO_ (eV)	ΔE(E_LUMO_−E_HOMO_) (eV)	χ (eV)	*μ* (eV)	*η* (eV)	*S* (e/V)	*ω* (eV)	ΔN
EZT	−0.6566	−5.9545	5.2979	3.3055	−3.3055	2.6489	0.1888	2.0625	1.2358
L-proline	−1.3189	−6.1460	4.8271	3.7325	−3.7325	2.4136	0.2072	2.8861	1.5464
EZT + L-proline	−1.3056	−6.1542	4.8485	3.7289	−3.7289	2.4243	0.2062	2.8693	1.5385
EZT + 2L-proline	−1.5026	−5.9280	4.4254	4.4666	−4.4666	2.2127	0.2259	4.5081	2.0999

#### Electrophilicity-Based Charge Transfer Descriptors

ECT descriptors are determined by the difference between ΔN_max_ values of interacting molecules.

$$\mathrm{ECT}=\left(\Delta N_{\max}\right)A-\left(\Delta N_{\max}\right)B,$$

where (ΔN_max_) *A* = −μ_A_/η_A_ and (ΔN_max_) *B* = −μ_B_/η_B_. Among two interacting molecules A and B, if ECT < 0 then charge flows from A to B, and if ECT > 0 then charge flows from B to A. From Table 4, it was found that ECT < 0 (−0.3106) for the interacting molecules ezetimibe (A) and L-proline (B), which indicates that charge flows from ezetimibe to L-proline. Therefore, ezetimibe acts as electron donor and L-proline act as electron acceptor. In a similar manner, the high value of chemical potential and the low value of the electrophilicity index for ezetimibe show its nucleophilic nature. The low value of chemical potential (*μ*) and the high value of the electrophilicity (*ω*) index for L-proline favor its electrophilic character.

#### Local Reactivity Descriptors

In addition to global reactivity descriptors, local reactivity descriptors can be used to locate the exact position of electrophilic and nucleophilic sites present in the cocrystal. Fukui functions were first introduced by Parr and Yang (1984). Hirshfeld atomic charges (for neutral, cation, and anion states) were used to calculate Fukui functions (*f*_k_^+^, *f*_k_^−^, *f*_k_^0^), softness (S_k_^+^, S_k_^−^, S_k_^0^), and local electrophilicity indices (*ω*_k_^+^, *ω*_k_^−^, *ω*_k_^0^). The calculated values of local reactivity descriptors of the cocrystal (EZT-L-proline) for all the atomic sites are listed in Supplementary Table S7. According to the calculated values, it was found that oxygen atoms of L-proline (O53) and EZT (O3) have maximum values of *f*_k_^+^, S_k_^+^, and *ω*_k_^+^ and are more prone to nucleophilic attack, whereas maximum values of *f*_k_^−^, S_k_^−^, and *ω*_k_^−^ of the hydrogen atom of L-proline (H68) show that it is prone to electrophilic attack.

## Conclusion

Combination of quantum chemical calculations and vibrational spectroscopy has gained much potential to study the physical and chemical properties of biological systems. The current studies on the EZT-L-proline cocrystal highlighted that the changes occur from the API to the cocrystal in terms of spectra and chemical reactivity. Results are concluded as follows:

➢ Vibrational studies shows that the OH group of the API (EZT) was connected through an H-bond with the COO^−^ group of the coformer (L-proline) in the cocrystal (EZT-L-proline), which was confirmed by the red shift in the wavenumber with elongation of the bond length of these groups. Spectral investigation reveals better match between experimental and calculated data, but somehow a large deviation in higher regions of the wavenumber appeared due to non-consideration of neighboring intermolecular hydrogen bond interactions. Calculations on EZT + 2L-proline removed such discrepancies of experimental and theoretical data.
➢ Changes in the environment of hydroxyl and carboxylate groups of the API and the coformer in the cocrystal (EZT + L-proline and EZT + 2L-proline) were found. The intermolecular hydrogen bond interaction of O5H of EZT in the cocrystal was not taken in case of EZT + L-proline, which has generated a mismatch of calculated and observed values, but it has been removed in EZT + 2L-proline because of inclusion of that interaction. Hence, calculated spectral findings of EZT + 2L-proline were more appropriate.
➢ NBO and AIM analyses were used to study hydrogen bonds present in the cocrystal. Charge transfer between LP(1)O52/LP(3)O52 → σ* (O4-H50) and LP(1)O69/LP(2)O69 → σ* (O5-H51) within the API and the coformer displayed the presence of O52•••H50 and O69•••H51 bonds, which was also complemented by AIM theory. (∇^2^ρ_BCP_) > 0 and HBCP < 0 suggest the moderate nature of these H-bonds.
➢ Reactivity was mainly found around hydroxyl and carboxylate groups according to the molecular electrostatic potential surface (MEPS). A higher value of molar refractivity (MR) shows that the cocrystal is more polarizable than the API.
➢ A smaller energy gap ensures that the cocrystal (4.4254 eV) is more reactive than the API (5.2979 eV); also, a higher value of electrophilicity index (*ω* = 2.8639 eV) of EZT-L-proline makes it a strong electrophile. Study of chemical reactivity descriptors of EZT and the ezetimibe-L-proline cocrystal shows that the chemical properties of EZT modify with the formation of the cocrystal with L-proline.
➢ ECT < 0 (−0.3106) suggests that charge transfer takes place from the API (ezetimibe) to the coformer (L-proline). Fukui functions predicted the particular reactive sites of the cocrystal. Local reactivity descriptors predicted that oxygen atoms of L-proline (O53) and EZT (O3) have preferred nucleophilic attack, whereas the hydrogen atom of L-proline (H68) is prone to electrophilic attack.

Thus, with the proper addition of a suitable coformer to an API, the pharmacokinetic properties of a drug can be modified. We hope that this work will provide a significant way to spectroscopists as well as to the pharmaceutical industry for the study of enhancement of physicochemical properties of active pharmaceutical ingredients.

## Data Availability

The original contributions presented in the study are included in the article/Supplementary Material, further inquiries can be directed to the corresponding authors.
