# Supplementary material for: Molecular Structural, Hydrogen Bonding Interactions, and Chemical Reactivity Studies of Ezetimibe-L-Proline Cocrystal Using Spectroscopic and Quantum Chemical Approach
Source: Front Chem. 2022 Feb 15;10:848014. doi: 10.3389/fchem.2022.848014 (PMC8885513; doi:10.3389/fchem.2022.848014)
Supplement: Supplementary file 1 [file DataSheet1.docx]

Supplementary Material

Molecular Structural, Hydrogen Bonding Interactions and Chemical Reactivity Studies of Ezetimibe-L-proline Cocrystal using Spectroscopic and Quantum Chemical Approach

Preeti Prajapatia, Jaya Pandeya, Poonam Tandon*a, Kirti Sinhaa, Manishkumar R. Shimpi*b,c

*aDepartment of Physics, University of Lucknow, Lucknow 226007, India*

*bChemistry of Interfaces, Luleå University of Technology, SE-971 87, Luleå, Sweden.*

*cDepartment of Materials and Environmental Chemistry, Stockholm University, Svante Arrhenius väg 16c, 10691 Stockholm, Sweden.*

Fig S1 and S2 represents the ground state optimized structure of EZT and L-proline. Fig S3-S6demonstrates the IR (400-3800 cm−1) and Raman (100-3800 cm−1) spectra of EZT and L-proline respectively. Changes occurred due to the formation of cocrystal among the OH groups of EZT and COO− group of L-proline are shown in Fig S7. Intermolecular hydrogen bonding interactions in EZT, L-proline and cocrystal has been shown in Fig S8-S10. Fig S11-S12 represented the molecular electrostatic potential surface (MEPS) map of EZT andL-proline respectively. HOMO-LUMO energy gap of EZT is shown in Fig S13. Comparison between experimental and optimized geometrical parameters of EZT, L-proline and EZT- L-proline (cocrystal) is shown in Table S1. Table S2, S3, S4 and S5 presented the detailed vibrational assignment of EZT, L-proline and EZT-L-prolinecocrystal (EZT+L-proline and EZT+2L-proline), respectively. Natural bond orbital (NBO) calculation for cocrystal is given in S6. Table S7 deeply presented the reactive sites of cocrystal by means of Fukui function.


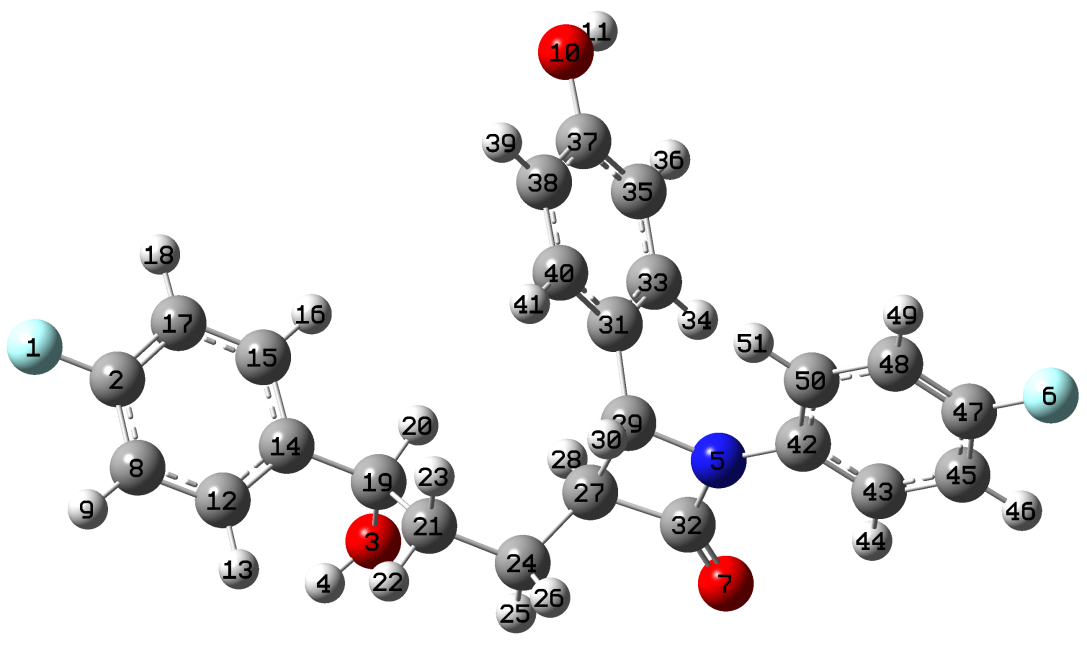


**Fig S1** Optimized structure of EZT using B3LYP/6-311++G(d,p) method.


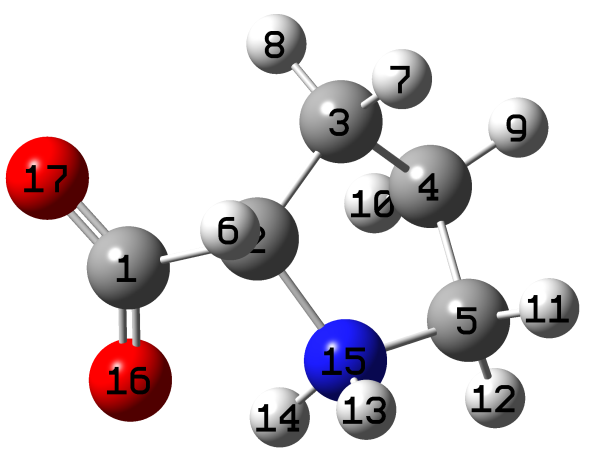


**Fig S2** Optimized structure of L-prolineusing B3LYP/6-311++G(d,p) method.


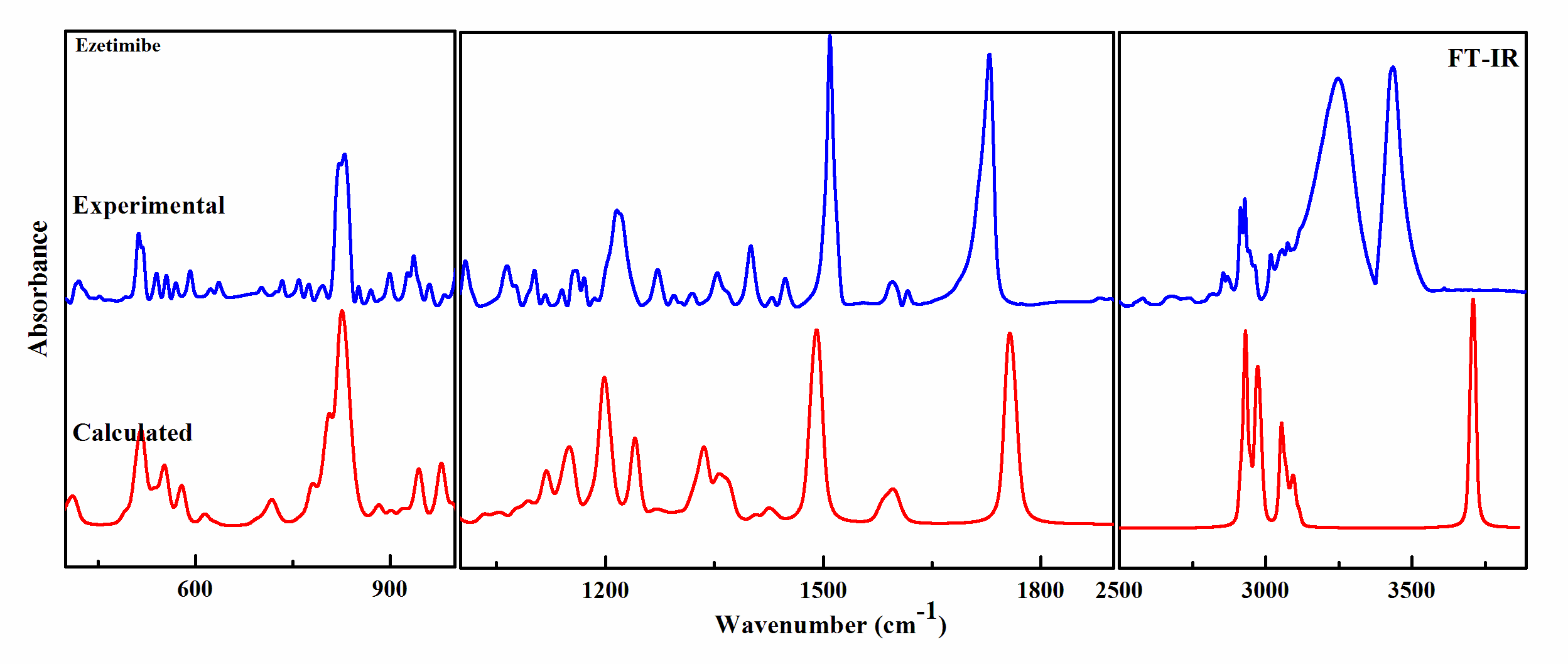


**Fig S3** Experimental FT-IR spectra of EZT with calculated IR spectra of EZT in the region 400-1000 cm−1, 1000-1900 cm−1 and 2500-3800 cm−1.


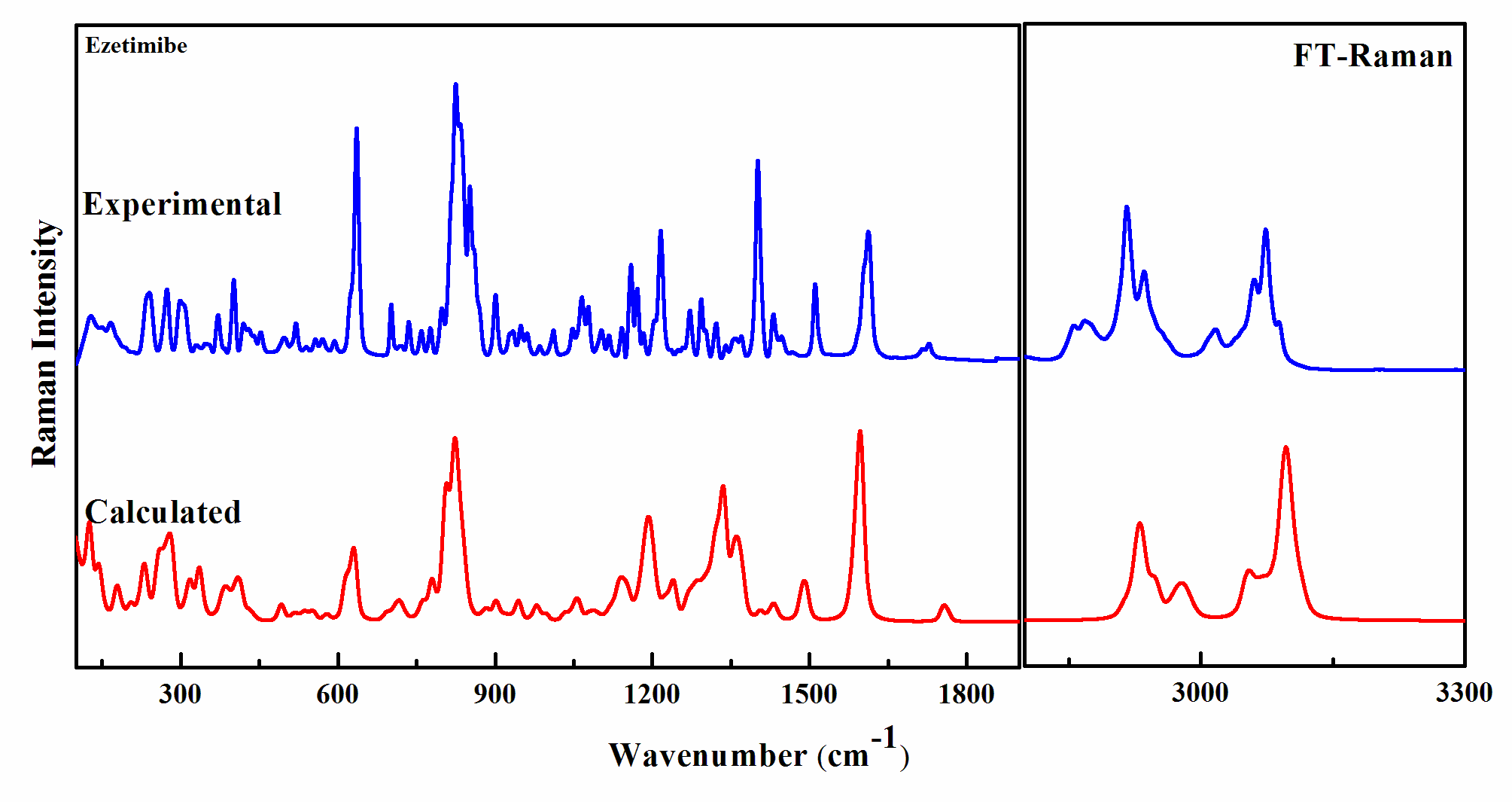


**Fig S4** Experimental FT-Raman spectra of EZT with calculated Raman spectra of EZT in the region 100-1900 cm−1 and 2700-3800 cm−1.


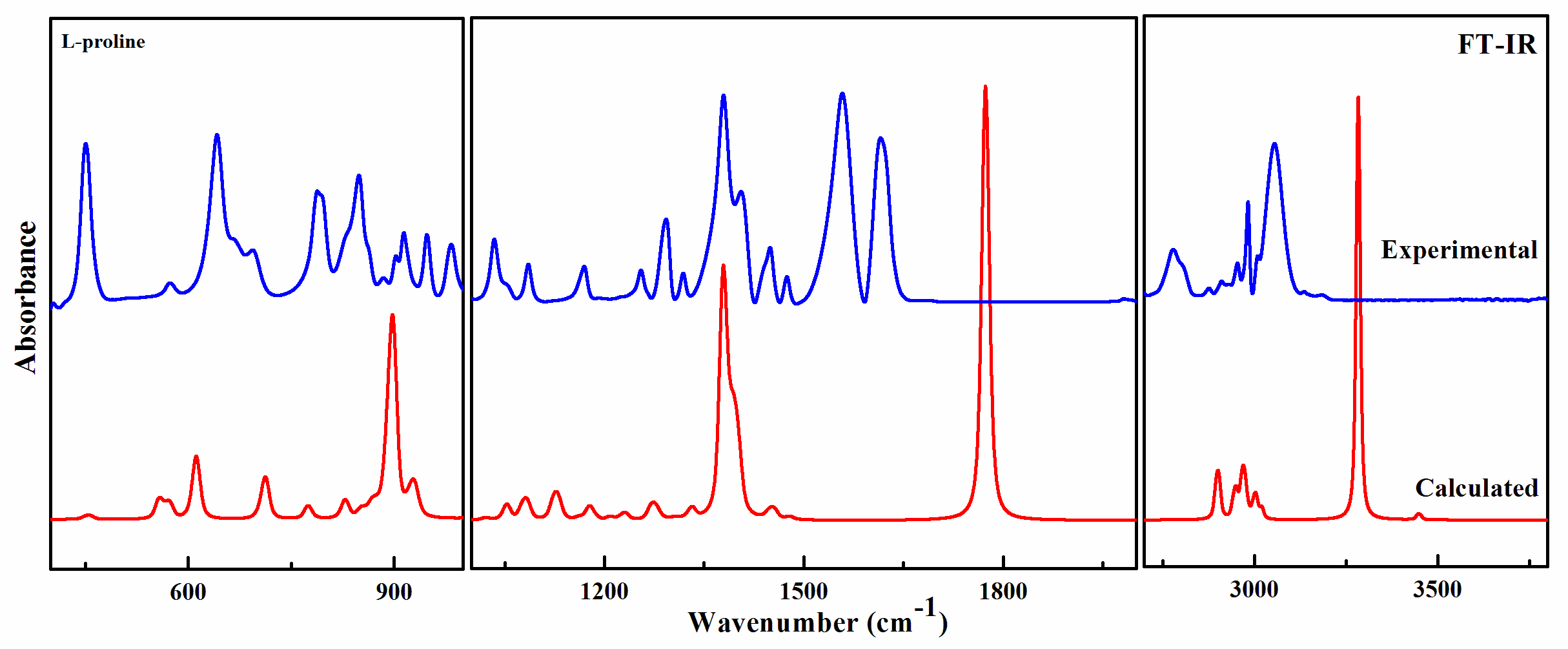


**Fig S5** Experimental FT-IR spectra of L-proline with calculated IR spectra ofL-proline in the region 400-1000 cm−1, 1000-2000 cm−1 and 2500-3800 cm−1.


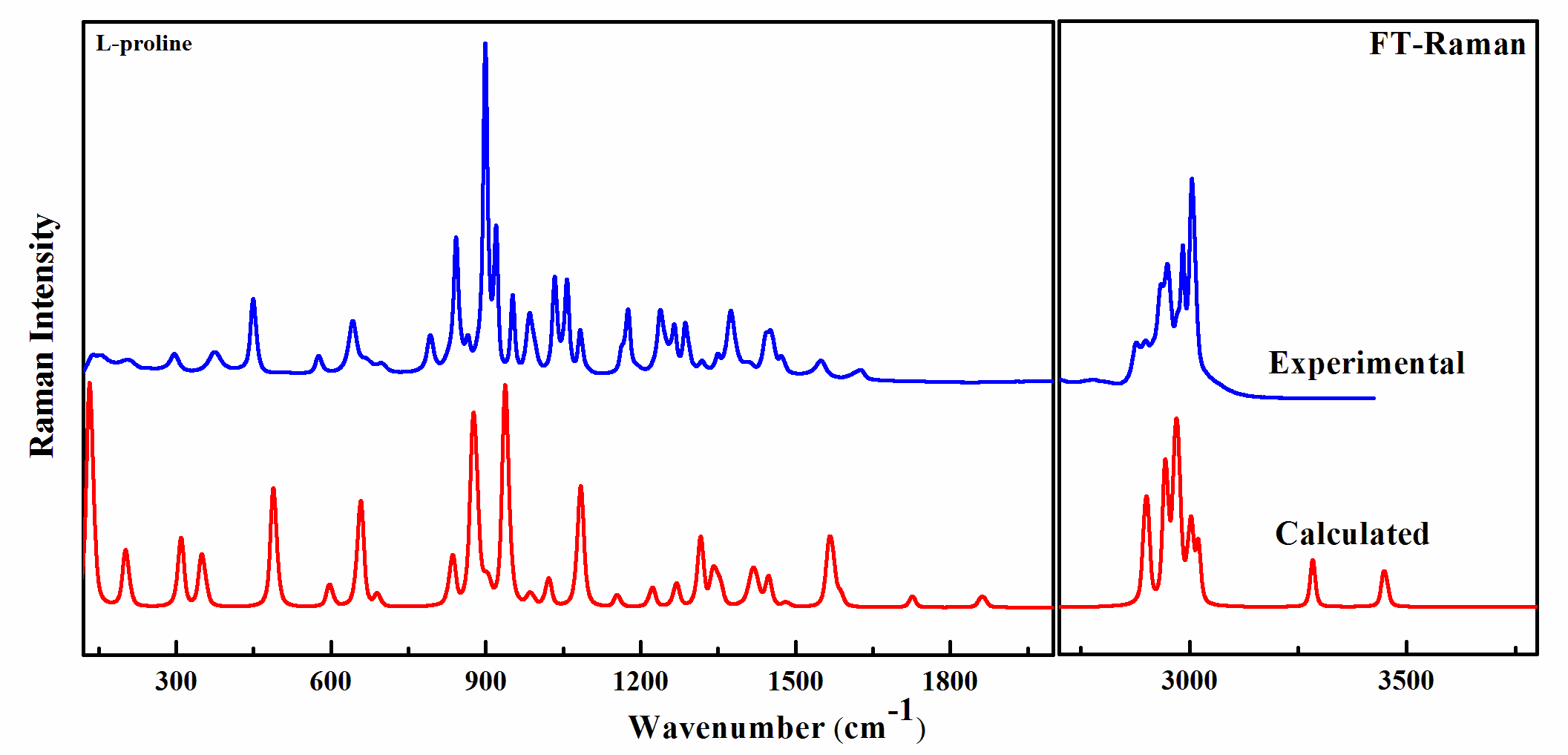


**Fig S6** Experimental FT-Raman spectra L-proline with calculated IR spectra of L-proline in the region 100-1900 cm−1 and 2700-3800 cm−1.


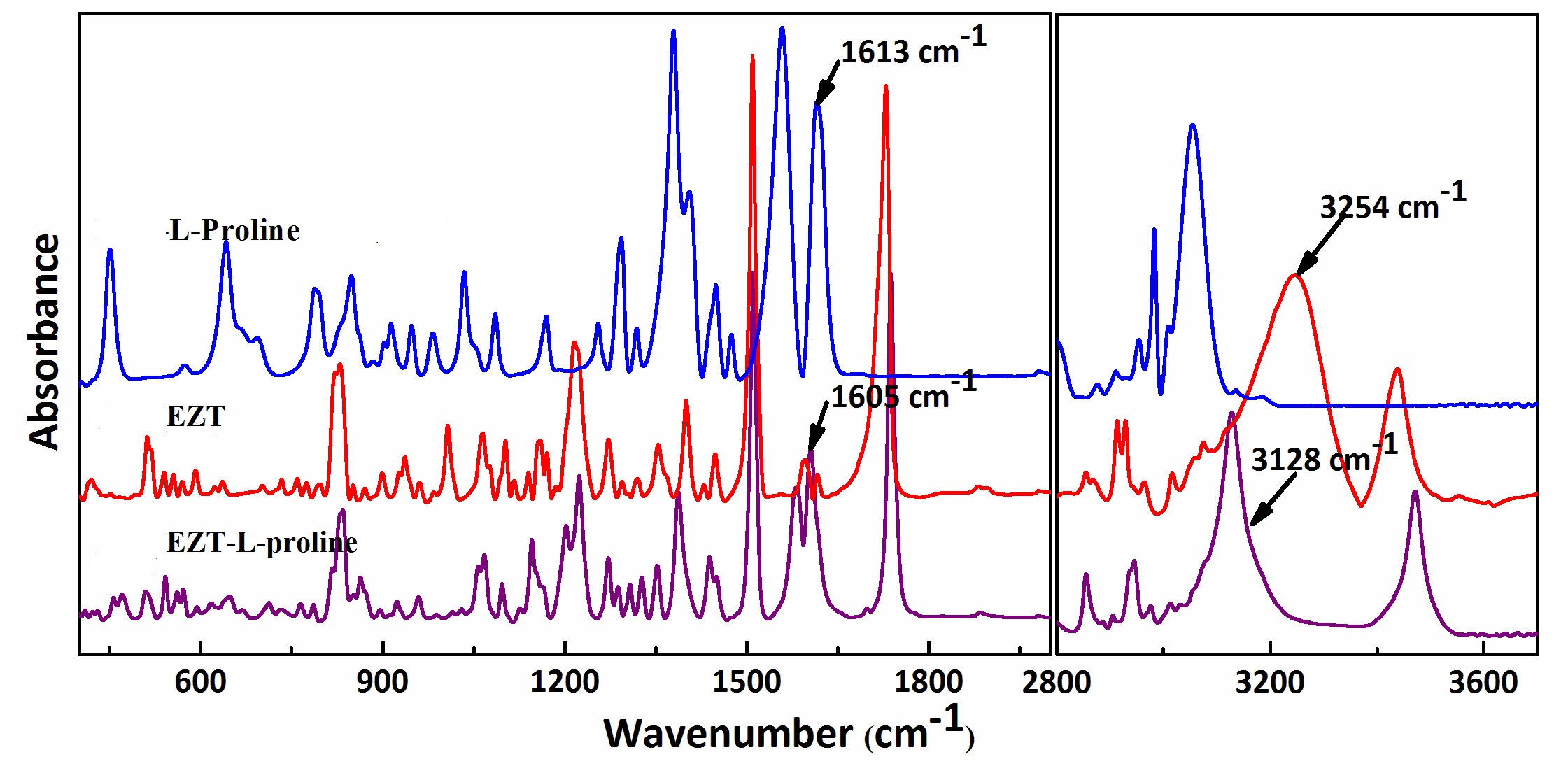


**Fig S7** Experimental FT-IR spectra of EZT-L-prolinecocrystal, EZT and L-proline in the region 400-1000 cm−1, 1000-2000 cm−1 and 2700-3800 cm−1 showing the changes occur in wavenumber of groups involved in cocrystal.


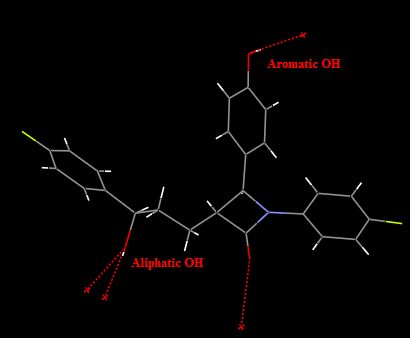


**Fig S8**Showing the intermolecular hydrogen bond interactions of ezetimibe.


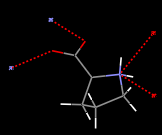


**Fig S9**Showing the intermolecular hydrogen bond interactions of L-proline.


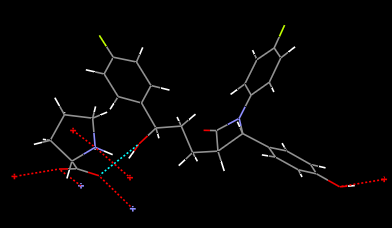


**Fig S10**Showing the intermolecular hydrogen bond interactions of cocrystal.


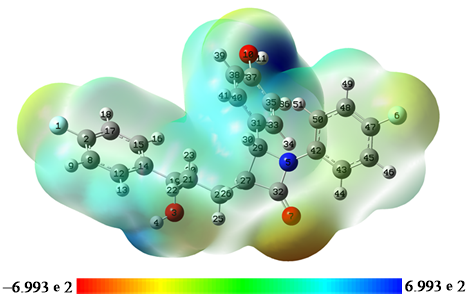


**Fig S11** Molecular electrostatic potential surface (MEPS) of EZTformed by mapping total density over electrostatic potential in gas phase.


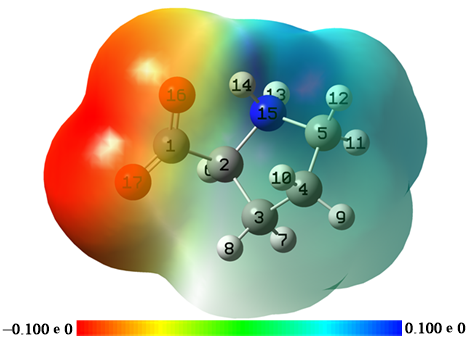


**Fig S12** Molecular electrostatic potential surface (MEPS) of L-proline formed by mapping total density over electrostatic potential in gas phase.


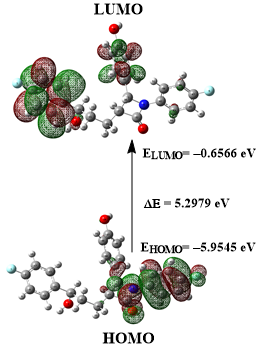


**Fig S13** HOMO-LUMO plot of EZT with orbitals involved in electronic transitions.

**Table S1** Comparison of experimental and theoretical stretching frequency (cm−1), bond length (Å) involved in hydrogen bonding.

| **Parameters** | **Experimental** | **Optimized** | | | **Parameters** | **Experimental** | **Optimized** | | |
| --- | --- | --- | --- | --- | --- | --- | --- | --- | --- |
|  | **EZT-L-proline**  **cocrystal** | **EZT and L-proline** | **EZT+L-proline** | **EZT+2L-proline** |  | **EZT-L-proline**  **cocrystal** | **EZT and L-proline** | **EZT+L-proline** | **EZT+2L-proline** |
| **Bond length**  **(Å)** | | | | | **Dihedral angle**  **(°)** | | | | |
| (F1-C10) | 1.3340 | 1.3581 | 1.3587 | 1.3586 | (H50-O4-C17-C16) | 133.66 | 81.04 | 168.36 | 169.67 |
| (F2-C21) | 1.3404 | 1.3552 | 1.3596 | 1.3584 | (H50-O4-C17-C18) | −97.83 | −46.70 | −69.35 | −67.76 |
| (O3−C13) | 1.1954 | 1.2068 | 1.2074 | 1.2134 | (H50−O4−C17−H40) | 16.03 | −162.18 | 50.42 | 52.45 |
| (O4−C17) | 1.3612 | 1.4385 | 1.4154 | 1.4237 | (C17−O4−O52−C55) | 90.94 | − | −33.28 | −125.63 |
| (O4−H50) | 1.0396 | 0.9631 | 0.9782 | 0.9766 | (O51−O5−C28−C27) | −163.12 | 178.95 | 179.80 | 179.84 |
| (O5−C28) | 1.3608 | 1.3673 | 1.3679 | 1.3537 | (O51−O5−C28−C29) | 17.05 | −0.76 | −0.56 | −0.58 |
| (O5−51) | 0.9922 | 0.9631 | 0.963 | 0.9894 | (C13−N6−C7−C8) | 154.65 | −171.31 | 171.25 | 171.18 |
| (N6−C7) | 1.3758 | 1.4040 | 1.4029 | 1.4038 | (C13−N6−C7−C12) | −24.48 | 8.53 | −8.58 | −8.60 |
| (N6−C13) | 1.3452 | 1.3854 | 1.3864 | 1.3786 | (C24−N6−C7−C8) | −0.15 | −2.02 | 0.24 | 1.40 |
| (N6−C24) | 1.4667 | 1.4835 | 1.482 | 1.4901 | (C24−N6−C7−C12) | −179.28 | 177.82 | −179.59 | −178.37 |
| (C7−C8) | 1.4767 | 1.4002 | 1.4005 | 1.4004 | (C7−N6−C13−O3) | 30.13 | −4.69 | 4.46 | 3.47 |
| (C7−C12) | 1.3666 | 1.4039 | 1.4041 | 1.4036 | (C7−N6−C13−C14) | −167.57 | 174.10 | −174.88 | −175.33 |
| (C8−C9) | 1.4252 | 1.3927 | 1.3927 | 1.3923 | (C24−N6−C13−O3) | −169.36 | −176.65 | 177.71 | 175.79 |
| (C8−H31) | 1.0896 | 1.0823 | 1.0824 | 1.0821 | (C24−N6−C13−C14) | −7.06 | 2.14 | −1.64 | −3.00 |
| (C9−C10) | 1.3110 | 1.3843 | 1.3843 | 1.3845 | (C7−N6−C24−C14) | 169.21 | −174.26 | 175.03 | 175.46 |
| (C9−H32) | 1.0768 | 1.0828 | 1.0828 | 1.0828 | (C7−N6−C24−C25) | −70.59 | 65.54 | −65.25 | −64.05 |
| (C10−C11) | 1.3357 | 1.3863 | 1.3862 | 1.3860 | (C7−N6−C24−H45) | 59.51 | −61.80 | 62.14 | 63.07 |
| (C11−C12) | 1.4208 | 1.3908 | 1.3909 | 1.3910 | (C13−N6−C24−C14) | 6.88 | −2.09 | 1.60 | 2.93 |
| (C11−H33) | 1.0802 | 1.0829 | 1.0829 | 1.0830 | (C13−N6−C24−C25) | 127.08 | −122.29 | 121.32 | 123.41 |
| (C12−H34) | 1.0956 | 1.0814 | 1.0814 | 1.0812 | (C13−N6−C24−H45) | −102.83 | 110.36 | −111.30 | −109.46 |
| (C13−C14) | 1.5511 | 1.5357 | 1.5371 | 1.5335 | (N6−C7−C8−C9) | −178.83 | 179.78 | −179.75 | −179.60 |
| (C14−C15) | 1.5155 | 1.5270 | 1.5298 | 1.5283 | (N6−C7−C8−H31) | 1.89 | −0.83 | 0.86 | 1.17 |
| (C14−C24) | 1.5654 | 1.5736 | 1.5744 | 1.5737 | (C12−C7−C8−C9) | 0.30 | −0.07 | 0.08 | 0.18 |
| (C14−H35) | 1.0899 | 1.0926 | 1.0932 | 1.0935 | (C12−C7−C8−H31) | −178.98 | 179.32 | −179.31 | −179.05 |
| (C15−C16) | 1.4722 | 1.5371 | 1.5331 | 1.5371 | (N6−C7−C12−C11) | 178.75 | −179.71 | 179.67 | 179.54 |
| (C15−H36) | 1.0859 | 1.0961 | 1.0971 | 1.0956 | (N6−C7−C12−H34) | −1.07 | −0.16 | 0.05 | −0.16 |
| (C15−H37) | 1.0919 | 1.0930 | 1.0921 | 1.0937 | (C8−C7−C12−C11) | −0.40 | 0.14 | −0.16 | −0.24 |
| (C16−C17) | 1.4790 | 1.5371 | 1.5362 | 1.5366 | (C8−C7−C12−H34) | 179.78 | 179.68 | −179.78 | −179.94 |
| (C16−H38) | 1.0890 | 1.0964 | 1.0959 | 1.0939 | (C7−C8−C9−C10) | −0.09 | −0.01 | 0.03 | −0.02 |
| (C16−H39) | 1.0905 | 1.0959 | 1.0952 | 1.0960 | (C7−C8−C9−H32) | −179.37 | 179.91 | −179.88 | −179.83 |
| (C17−C18) | 1.4904 | 1.5372 | 1.5264 | 1.5209 | (H31−C8−C9−C10) | 179.15 | −179.40 | 179.42 | 179.21 |
| (C17−H40) | 1.0864 | 1.0924 | 1.1003 | 1.0999 | (H31−C8−C9−32) | −0.14 | 0.51 | −0.49 | −0.59 |
| (C18−C19) | 1.3966 | 1.3982 | 1.3973 | 1.3966 | (C8−C9−C10−F1) | 179.77 | 179.99 | 180.00 | −179.97 |
| (C18−C23) | 1.3845 | 1.4009 | 1.4003 | 1.3989 | (C8−C9−C10−C11) | −0.02 | 0.01 | −0.05 | −0.08 |
| (C19−C20) | 1.4304 | 1.3934 | 1.3978 | 1.3948 | (H32−C9−C10−F1) | −0.88 | 0.08 | −0.09 | −0.16 |
| (C19−H41) | 1.0862 | 1.0846 | 1.0855 | 1.0854 | (H32−C9−C10−C11) | 179.33 | −179.90 | 179.86 | 179.73 |
| (C20−C21) | 1.3781 | 1.3850 | 1.3867 | 1.3842 | (F1−C10−C11−C12) | −179.88 | −179.92 | 179.92 | 179.92 |
| (C20−H42) | 1.0924 | 1.0829 | 1.0835 | 1.0829 | (F1−C10−C11−H33) | 0.11 | 0.12 | −0.08 | −0.02 |
| (C21−C22) | 1.3477 | 1.3862 | 1.3888 | 1.3867 | (C9−C10−C11−C12) | −0.09 | 0.06 | −0.04 | 0.02 |
| (C22−C23) | 1.3760 | 1.3927 | 1.3939 | 1.3921 | (C9−C10−C11−H33) | 179.91 | −179.90 | 179.97 | −179.91 |
| (C22−H43) | 1.0887 | 1.0829 | 1.0835 | 1.0831 | (C10−C11−C12−C7) | 0.30 | −0.13 | 0.14 | 0.13 |
| (C23−H44) | 1.0814 | 1.0850 | 1.0836 | 1.0836 | (C10−C11−C12−H34) | −179.89 | −179.67 | 179.76 | 179.83 |
| (C24−C25) | 1.4884 | 1.5086 | 1.5081 | 1.5056 | (H33−C11−C12−C7) | −179.69 | 179.83 | −179.87 | −179.94 |
| (C24−H45) | 1.0901 | 1.0955 | 1.0945 | 1.0938 | (H33−C11−C12−H34) | 0.12 | 0.29 | −0.25 | −0.24 |
| (C25−C26) | 1.3534 | 1.3989 | 1.3991 | 1.3988 | (O3−C13−C14−C15) | −68.17 | 56.68 | −56.26 | −53.16 |
| (C25−C30) | 1.3773 | 1.3981 | 1.3978 | 1.3996 | (O3−C13−C14−C24) | 168.84 | 176.69 | −177.75 | −175.88 |
| (C26−C27) | 1.3457 | 1.3908 | 1.3906 | 1.3906 | (O3−C13−C14−H35) | 59.64 | −71.66 | 71.68 | 73.76 |
| (C26−H46) | 1.0911 | 1.0858 | 1.0858 | 1.0863 | (N6−C13−C14−C15) | 129.61 | −122.03 | 123.03 | 125.56 |
| (C27−C28) | 1.3520 | 1.3908 | 1.3946 | 1.3985 | (N6−C13−C14−C24) | 6.62 | −2.01 | 1.54 | 2.84 |
| (C27−H47) | 1.0761 | 1.0832 | 1.0832 | 1.0837 | (N6−C13−C14−H35) | −102.58 | 109.63 | −109.02 | −107.52 |
| (C28−C29) | 1.3466 | 1.3969 | 1.3965 | 1.4026 | (C13−C14−C15−C16) | −40.30 | 48.06 | −43.60 | −56.81 |
| (C29−C30) | 1.4231 | 1.3912 | 1.3913 | 1.3900 | (C13−C14−C15−H36) | −159.32 | 170.30 | −167.11 | 178.98 |
| (C29−H48) | 1.0796 | 1.0861 | 1.0861 | 1.0845 | (C13−C14−C15−H37) | 83.35 | −67.34 | 78.02 | 64.09 |
| (C30−H49) | 1.0962 | 1.0846 | 1.0844 | 1.0853 | (C24−C14−C15−C16) | 55.16 | 70.43 | 58.31 | 45.22 |
| (H50−O52) | 2.0348 | − | 1.8249 | 1.8369 | (C24−C14−C15−H36) | −63.85 | −51.80 | −65.20 | −78.99 |
| (O52−C55) | 1.2105 | 1.2050 | 1.2803 | 1.2740 | (C24−C14−C15−H37) | 178.81 | −167.21 | 179.93 | 166.12 |
| (O53−C55) | 1.2103 | 1.2402 | 1.2216 | 1.2237 | (H35−C14−C15−C16) | −171.43 | 176.19 | −172.06 | 175.99 |
| (N54−C56) | 1.4875 | 1.5124 | 1.5201 | 1.5243 | (H35−C14−C15−H36) | 69.55 | −61.58 | 64.42 | 51.78 |
| (N54−C59) | 1.5090 | 1.4928 | 1.5017 | 1.5128 | (H35−C14−C15−H37) | −47.78 | 60.78 | −50.45 | −63.11 |
| (N54−H60) | 1.0134 | 1.0434 | 1.0198 | 1.0166 | (C13−C14−C24−N6) | −6.01 | 1.88 | −1.44 | −2.63 |
| (N54−H61) | 1.0134 | 1.0015 | 1.0846 | 1.0672 | (C13−C14−C24−C25) | −124.85 | 120.82 | −120.33 | −121.95 |
| (C55−C56) | 1.5297 | 1.5686 | 1.5739 | 1.5795 | (C13−C14−C24−H45) | 105.86 | −109.90 | 110.64 | 108.41 |
| (C56−C57) | 1.5217 | 1.5275 | 1.5348 | 1.5314 | (C15−C14−C24−N6) | −117.84 | 119.03 | −121.48 | −121.68 |
| (C56−H62) | 1.0815 | 1.0804 | 1.0884 | 1.0897 | (C15−C14−C24−C25) | 123.32 | −122.03 | 119.63 | 119.00 |
| (C57−C58) | 1.5251 | 1.5275 | 1.5382 | 1.5373 | (C15−C14−C24−H45) | −5.98 | 7.24 | −9.40 | −10.65 |
| (C57−H63) | 1.0884 | 1.0802 | 1.0896 | 1.0895 | (H35−C14−C24−N6) | 109.11 | −109.07 | 109.31 | −121.68 |
| (C57−H64) | 1.0865 | 1.0871 | 1.0943 | 1.0950 | (H35−C14−C24−C25) | −9.73 | 9.87 | −9.58 | −141.19 |
| (C58−C59) | 1.5332 | 1.5266 | 1.5307 | 1.5294 | (H35−C14−C24−H45) | −139.03 | 139.14 | −138.61 | −141.19 |
| (C58−H65) | 1.0887 | 1.0829 | 1.0929 | 1.0926 | (C14−C15−C16−C17) | 177.41 | −175.36 | −177.63 | 155.24 |
| (C58−H66) | 1.0883 | 1.0845 | 1.0907 | 1.0905 | (C14−C15−C16−H38) | −60.57 | 62.43 | −56.41 | −82.59 |
| (C59−H67) | 1.0883 | 1.0808 | 1.0898 | 1.0874 | (C14−C15−C16−H39) | 59.63 | −59.82 | 61.72 | 35.85 |
| (C59−H68) | 1.0864 | 1.0831 | 1.0918 | 1.0917 | (H36−C15−C16−C17) | −64.00 | −52.91 | −54.95 | −80.89 |
| **Bond angles (°)** | | | | | (H36−C15−C16−H38) | 58.015 | 62.64 | 66.26 | 41.28 |
| (C17−O4−H50) | 118.19 | 108.96 | 107.94 | 111.83 | (H36−C15−C16−H39) | 178.21 | −175.11 | −175.60 | 159.72 |
| (C28−O5−H51) | 121.48 | 109.94 | 109.89 | 111.16 | (H37−C15−C16−C17) | 55.69 | −59.10 | 60.74 | 35.20 |
| (C7−N6−C13) | 134.67 | 133.06 | 133.16 | 133.16 | (H37−C15−C16−H38) | 177.71 | 178.65 | −178.04 | 157.36 |
| (C7−N6−C24) | 128.55 | 131.40 | 131.40 | 131.42 | (H37−C15−C16−H39) | −62.09 | 63.10 | −59.91 | −84.19 |
| (C13−N6−C24) | 93.73 | 94.99 | 95.05 | 94.92 | (C15−C16−C17−O4) | −57.08 | 53.38 | −59.31 | −66.23 |
| (N6−C7−C8) | 121.33 | 120.12 | 120.09 | 119.97 | (C15−C16−C17−C18) | −179.37 | −179.73 | 177.84 | 170.77 |
| (N6−C7−C12) | 119.18 | 120.17 | 120.25 | 120.35 | (C15−C16−C17−H40) | 57.10 | −60.36 | 59.68 | 51.64 |
| (C8−C7−C12) | 119.48 | 119.71 | 119.65 | 119.68 | (H38−C16−C17−O4) | −178.01 | 176.91 | 177.69 | 170.55 |
| (C7−C8−C9) | 119.65 | 120.29 | 120.32 | 120.28 | (H38−C16−C17−C18) | 59.71 | −56.20 | 54.84 | 47.55 |
| (C7−C8−H31) | 116.66 | 120.22 | 120.18 | 120.13 | (H38−C16−C17−H40) | −63.82 | 63.17 | −63.32 | −71.57 |
| (C9−C8−H31) | 123.69 | 119.49 | 119.50 | 119.58 | (H39−C16−C17−O4) | 61.70 | −68.19 | 62.12 | 55.41 |
| (C8−C9−C10) | 118.87 | 118.89 | 118.90 | 118.93 | (H39−C16−C17−C18) | −60.58 | 58.71 | −60.74 | −67.59 |
| (C8−C9−H32) | 124.70 | 121.19 | 121.17 | 121.15 | (H39−C16−C17−H40) | 175.88 | 178.08 | −178.90 | 173.29 |
| (C10−C9−H32) | 116.43 | 119.92 | 119.93 | 119.92 | (O4−C17−C18−C19) | 164.74 | −104.94 | 141.37 | 137.58 |
| (F1−C10−C9) | 120.23 | 119.00 | 119.00 | 119.03 | (O4−C17−C18−C23) | −13.85 | 73.14 | −39.28 | −43.83 |
| (F1−C10−C11) | 116.75 | 119.04 | 119.05 | 119.01 | (C16−C17−C18−C19) | −72.39 | 128.34 | −98.12 | −103.16 |
| (C9−C10−C11) | 123.01 | 121.96 | 121.95 | 121.96 | (C16−C17−C18−C23) | 109.02 | −53.57 | 81.23 | 75.43 |
| (C10−C11−C12) | 120.63 | 119.23 | 119.23 | 119.18 | (H40−C17−C18−C19) | 52.55 | 8.26 | 20.35 | 16.39 |
| (C10−C11−H33) | 118.21 | 119.79 | 119.79 | 119.76 | (H40−C17−C18−C23) | −126.03 | −173.65 | −160.29 | −165.02 |
| (C12−C11−H33) | 121.16 | 120.98 | 120.98 | 121.05 | (C17−C18−C19−C20) | −178.46 | 178.29 | 179.44 | 178.04 |
| (C7−C12−C11) | 118.36 | 119.92 | 119.95 | 119.96 | (C17−C18−C19−H41) | 2.32 | −1.59 | −1.14 | −2.53 |
| (C7−C12−H34) | 118.26 | 120.98 | 119.49 | 119.50 | (C23−C18−C19−C20) | 0.17 | 0.14 | 0.07 | −0.58 |
| (C11−C12−H34) | 123.38 | 120.57 | 120.56 | 120.54 | (C23−C18−C19−H41) | −179.05 | −179.74 | 179.50 | 178.86 |
| (O3−C13−N6) | 131.00 | 132.02 | 131.78 | 132.02 | (C17−C18−C23−C22) | 178.46 | −177.85 | −179.29 | −178.15 |
| (O3−C13−C14) | 131.26 | 135.83 | 136.14 | 135.55 | (C17−C18−C23−H44) | −1.93 | 2.35 | 1.70 | 2.86 |
| (N6−C13−C14) | 94.96 | 92.13 | 92.08 | 92.41 | (C19−C18−C23−C22) | −0.29 | 0.27 | 0.08 | 0.46 |
| (C13−C14−C15) | 112.54 | 115.75 | 118.08 | 116.95 | (C19−C18−C23−H44) | 179.45 | −179.53 | −178.93 | −178.53 |
| (C13−C14−C24) | 82.08 | 85.76 | 85.72 | 85.79 | (C18−C19−C20−C21) | 0.20 | −0.38 | −0.12 | 0.23 |
| (C13−C14−H35) | 116.23 | 111.02 | 110.91 | 110.57 | (C18−C19−C20−H42) | −179.42 | 179.88 | −179.51 | 179.53 |
| (C15−C14−C24) | 123.43 | 118.77 | 119.65 | 120.92 | (H41−C19−C20−C21) | 179.41 | 179.50 | −179.55 | −179.20 |
| (C15−C14−H35) | 109.79 | 111.46 | 109.81 | 110.00 | (H41−C19−C20−H42) | −0.21 | −0.24 | 1.06 | 0.09 |
| (C24−C14−H35) | 110.68 | 111.71 | 110.72 | 110.53 | (C19−C20−C21−F2) | 178.92 | −179.86 | 179.87 | 179.81 |
| (C14−C15−C16) | 111.49 | 115.00 | 115.11 | 115.57 | (C19−C20−C21−C22) | −0.48 | 0.22 | 0.02 | 0.25 |
| (C14−C15−H36) | 108.51 | 109.03 | 108.36 | 109.11 | (H42−C20−C21−F2) | −1.44 | −0.12 | −0.72 | 0.50 |
| (C14−C15−H37) | 107.80 | 107.86 | 108.45 | 106.93 | (H42−C20−C21−C22) | 179.16 | 179.97 | 179.43 | −179.06 |
| (C16−C15−H36) | 111.88 | 109.39 | 109.92 | 109.11 | (F2−C21−C22−C23) | −179.03 | −179.75 | −179.73 | −179.92 |
| (C16−C15−H37) | 108.53 | 108.61 | 108.42 | 108.51 | (F2−C21−C22−C43) | 0.98 | −0.06 | 0.84 | −0.21 |
| (H36−C15−H37) | 108.51 | 106.62 | 106.20 | 109.75 | (C20−C21−C22−C23) | 0.37 | 0.17 | 0.12 | −0.36 |
| (C15−C16−C17) | 110.85 | 114.64 | 112.40 | 112.38 | (C20−C21−C22−H43) | −179.62 | 179.85 | −179.31 | 179.36 |
| (C15−C16−H38) | 108.93 | 110.40 | 111.16 | 110.82 | (C21−C22−C23−C18) | 0.03 | −0.42 | −0.17 | 0.00 |
| (C15−C16−H39) | 108.97 | 108.13 | 109.72 | 110.74 | (C21−C22−C23−H44) | −179.73 | 179.38 | 178.82 | 178.98 |
| (C17−C16−H38) | 110.78 | 108.01 | 108.02 | 108.95 | (H43−C22−C23−C18) | −179.98 | 179.90 | 179.24 | −179.72 |
| (C17−C16−H39) | 107.33 | 109.31 | 108.36 | 106.83 | (H43−C22−C23−H44) | 0.26 | −0.30 | −1.76 | −0.73 |
| (H38−C16−H39) | 110.15 | 106.00 | 106.99 | 106.89 | (N6−C24−C25−C26) | 167.11 | −144.08 | 148.08 | 143.47 |
| (O4−C17−C16) | 108.52 | 111.36 | 107.87 | 106.07 | (N6−C24−C25−C30) | −13.77 | 37.68 | −34.03 | −38.45 |
| (O4−C17−C18) | 107.37 | 111.70 | 111.88 | 112.51 | (C14−C24−C25−C26) | −90.04 | 114.13 | −110.32 | −114.66 |
| (O4−C17−H40) | 104.80 | 103.78 | 109.95 | 109.68 | (C14−C24−C25−C30) | 89.07 | −64.12 | 67.58 | 63.42 |
| (C16−C17−C18) | 117.95 | 113.27 | 110.95 | 111.83 | (H45−C24−C25−C26) | 37.61 | −16.08 | 19.88 | 15.97 |
| (C16−C17−H40) | 110.12 | 108.70 | 108.33 | 108.71 | (H45−C24−C25−C30) | −143.28 | 165.67 | −162.23 | −165.95 |
| (C18−C17−H40) | 107.28 | 107.46 | 107.81 | 107.98 | (C24−C25−C26−C27) | 179.55 | −177.71 | 177.43 | 177.62 |
| (C17−C18−C19) | 120.59 | 121.56 | 121.18 | 120.62 | (C24−C25−C26−H46) | −0.32 | 2.50 | −2.53 | −2.37 |
| (C17−C18−C23) | 121.25 | 120.13 | 120.30 | 120.75 | (C30−C25−C26−C27) | 0.37 | 0.60 | −0.54 | −0.53 |
| (C19−C18−C23) | 118.14 | 118.28 | 118.51 | 118.62 | (C30−C25−C26−H46) | −179.50 | −179.19 | 179.50 | 179.47 |
| (C18−C19−C20) | 119.51 | 121.40 | 121.34 | 121.26 | (C24−C25−C30−C29) | −179.17 | 177.92 | −177.72 | −177.92 |
| (C18−C19−H41) | 119.14 | 119.45 | 119.58 | 119.64 | (C24−C25−C30−H49) | 1.16 | −2.90 | 3.07 | 3.00 |
| (C20−C19−H41) | 121.35 | 119.15 | 119.07 | 119.10 | (C26−C25−C30−C29) | −0.03 | −0.36 | 0.22 | 0.19 |
| (C19−C20−C21) | 118.59 | 118.40 | 118.31 | 118.30 | (C26−C25−C30−H49) | −179.70 | 178.82 | −179.00 | −178.89 |
| (C19−C20−H42) | 123.57 | 121.72 | 121.79 | 121.77 | (C25−C26−C27−C28) | −0.40 | −0.40 | 0.43 | 0.41 |
| (C21−C20−H42) | 117.84 | 119.88 | 119.90 | 119.93 | (C25−C26−C27−H47) | −179.93 | −179.86 | −179.91 | −179.59 |
| (F2−C21−C20) | 118.95 | 118.97 | 118.93 | 118.93 | (H46−C26−C27−C28) | 179.48 | 179.39 | −179.61 | −179.59 |
| (F2−C21−C22) | 119.02 | 118.84 | 118.93 | 118.82 | (H46−C26−C27−H47) | −0.05 | −0.07 | 0.06 | 0.06 |
| (C20−C21−C22) | 122.03 | 122.19 | 122.14 | 122.25 | (C26−C27−C28−O5) | −179.78 | −179.78 | 179.66 | 179.65 |
| (C21−C22−C23) | 119.40 | 118.46 | 118.54 | 118.52 | (C26−C27−C28−C29) | 0.05 | −0.05 | 0.00 | 0.05 |
| (C21−C22−H43) | 120.97 | 119.82 | 119.75 | 119.73 | (H47−C27−C28−O5) | −0.26 | −0.30 | −0.01 | −0.01 |
| (C23−C22−H43) | 119.63 | 121.72 | 121.70 | 121.75 | (H47−C27−C28−C29) | 179.56 | 179.42 | −179.66 | −179.61 |
| (C18−C23−C22) | 122.33 | 121.27 | 121.17 | 121.05 | (O5−C28−C29−C30) | −179.88 | 180.00 | −179.95 | −179.96 |
| (C18−C23−H44) | 121.10 | 120.36 | 118.87 | 119.31 | (O5−C28−C29−H48) | −0.63 | 0.31 | −0.22 | −0.25 |
| (C22−C23−H44) | 116.57 | 118.37 | 119.96 | 119.63 | (C27−C28−C29−C30) | 0.29 | 0.28 | −0.32 | −0.38 |
| (N6−C24−C14) | 88.46 | 87.04 | 87.11 | 86.74 | (C27−C28−C29−H48) | 179.54 | −179.40 | 179.42 | 179.32 |
| (N6−C24−C25) | 116.09 | 116.77 | 116.78 | 117.19 | (C28−C29−C30−C25) | −0.30 | −0.08 | 0.20 | 0.26 |
| (N6−C24−H45) | 111.15 | 111.29 | 111.50 | 110.70 | (C28−C29−C30−H49) | 179.36 | −179.26 | 179.42 | 179.35 |
| (C14−C24−C25) | 117.61 | 118.15 | 117.69 | 118.46 | (H48−C29−C30−C25) | −179.50 | 179.61 | −179.53 | −179.44 |
| (C14−C24−H45) | 108.91 | 111.97 | 112.34 | 112.08 | (H48−C29−C30−H49) | 0.17 | 0.43 | −0.31 | −0.35 |
| (C25−C24−H45) | 112.38 | 109.95 | 109.83 | 109.92 | (H50−O52−C55−O53) | 78.50 | − | −59.07 | −179.60 |
| (C24−C25−C26) | 119.51 | 120.24 | 120.09 | 119.96 | (H50−O52−C55−C56) | 104.72 | − | 120.70 | −79.68 |
| (C24−C25−C30) | 123.92 | 121.61 | 121.73 | 121.99 | (C59−N54−C56−C55) | −104.72 | 113.82 | 118.79 | 114.32 |
| (C26−C25−C30) | 116.56 | 118.13 | 118.15 | 118.03 | (C59−N54−C56−C57) | −6.36 | −6.36 | −1.75 | −6.21 |
| (C25−C26−C27) | 123.27 | 121.50 | 121.47 | 121.39 | (C59−N54−C56−H62) | −150.11 | −128.74 | −124.26 | −128.72 |
| (C25−C26−H46) | 119.65 | 119.57 | 119.54 | 119.55 | (H60−N54−C56−C55) | −154.73 | −119.45 | −113.01 | −119.38 |
| (C27−C26−H46) | 117.08 | 118.92 | 118.98 | 119.07 | (H60−N54−C56−C57) | 85.25 | 120.37 | 126.44 | 120.09 |
| (C26−C27−C28) | 121.96 | 119.57 | 119.58 | 120.02 | (H60−N54−C56−H62) | 31.90 | −2.01 | 3.93 | −2.42 |
| (C26−C27−H47) | 117.99 | 121.26 | 121.27 | 121.04 | (H61−N54−C56−C55) | 36.67 | −3.67 | 1.35 | −4.03 |
| (C28−C27−H47) | 115.52 | 119.17 | 119.15 | 118.94 | (H61−N54−C56−C57) | 156.68 | −123.85 | −119.20 | −124.56 |
| (O5−C28−C27) | 121.59 | 117.49 | 117.48 | 118.27 | (H61−N54−C56−H62) | 86.16 | 113.77 | 118.30 | 112.93 |
| (O5−C28−C29) | 121.14 | 122.73 | 122.72 | 122.48 | (C56−N54−C59−C58) | 11.29 | −18.39 | −21.91 | −17.99 |
| (C27−C28−C29) | 117.28 | 119.79 | 119.80 | 119.24 | (C56−N54−C59−H67) | 108.25 | −141.39 | −144.61 | −140.84 |
| (C28−C29−C30) | 121.49 | 119.99 | 119.99 | 120.01 | (C56−N54−C59−H68) | 130.87 | 99.85 | 96.55 | 100.02 |
| (C28−C29−H48) | 115.52 | 120.05 | 120.05 | 119.39 | (H60−N54−C59−C58) | −107.72 | −145.63 | −150.03 | −144.82 |
| (C30−C29−H48) | 122.98 | 119.96 | 119.97 | 120.60 | (H60−N54−C59−H67) | 132.75 | 91.37 | 87.27 | 92.32 |
| (C25−C30−C29) | 119.41 | 121.02 | 121.01 | 121.31 | (H60−N54−C59−H68) | 5.65 | −27.39 | −31.57 | −26.81 |
| (C25−C30−H49) | 119.49 | 119.88 | 119.84 | 119.81 | (H61−N54−C59−C58) | 135.86 | 89.61 | 84.20 | 90.42 |
| (C29−C30−H49) | 121.07 | 119.10 | 119.14 | 118.88 | (H61−N54−C59−H67) | 7.67 | −33.39 | −38.50 | −32.44 |
| (H50−O52−C55) | 134.53 | − | 130.50 | 135.33 | (H61−N54−C59−H68) | −104.56 | −152.15 | −157.34 | −151.57 |
| (C56−N54−C59) | 108.08 | 109.18 | 109.09 | 109.11 | (O52−C55−C56−N54) | −147.00 | 3.03 | −1.95 | 6.60 |
| (C56−N54−H60) | 112.86 | 113.60 | 113.68 | 113.45 | (O52−C55−C56−C57) | 149.59 | 116.81 | 112.15 | 120.73 |
| (C56−N54−H61) | 109.78 | 98.23 | 96.38 | 98.27 | (O52−C55−C56−H62) | −87.27 | −114.69 | −119.59 | −111.03 |
| (C59−N54−H60) | 108.53 | 112.71 | 113.81 | 112.51 | (O53−C55−C56−N54) | 147.41 | −176.60 | 177.86 | −173.62 |
| (C59−N54−H61) | 111.46 | 112.61 | 113.36 | 113.39 | (O53−C55−C56−C57) | −33.64 | −62.82 | −68.04 | −59.49 |
| (H60−N54−H61) | 106.05 | 109.70 | 109.32 | 109.36 | (O53−C55−C56−H62) | 89.51 | 65.68 | 60.22 | −68.76 |
| (O52−C55−O53) | 119.95 | 132.35 | 130.72 | 130.99 | (N54−C56−C57−C58) | 15.01 | 28.48 | 24.65 | 27.84 |
| (O52−C55−C56) | 119.95 | 112.62 | 112.15 | 112.76 | (N54−C56−C57−H63) | 160.69 | 150.26 | 146.60 | 149.63 |
| (O53−C55−C56) | 120.02 | 115.02 | 117.12 | 116.24 | (N54−C56−C57−H64) | −27.24 | −89.68 | −93.83 | −90.70 |
| (N54−C56−C55) | 111.42 | 104.96 | 104.62 | 105.25 | (C55−C56−C57−C58) | −77.81 | −85.53 | −89.30 | −86.64 |
| (N54−C56−C57) | 102.06 | 104.56 | 104.88 | 104.65 | (C55−C56−C57−H63) | 41.29 | 36.25 | 32.65 | 35.14 |
| (N54−C56−H62) | 110.57 | 109.78 | 110.01 | 109.79 | (C55−C56−C57−H64) | −11.81 | 156.31 | 152.22 | 154.81 |
| (C55−C56−C57) | 112.28 | 113.90 | 114.24 | 114.06 | (H62−C56−C57−C58) | 159.05 | 148.24 | 144.81 | 147.70 |
| (C55−C56−H62) | 110.13 | 109.36 | 109.01 | 108.81 | (H62−C56−C57−H63) | −81.85 | −89.98 | −93.24 | −90.51 |
| (C57−C56−H62) | 110.15 | 113.75 | 113.59 | 113.80 | (H62−C56−C57−H64) | 39.89 | 30.08 | 26.32 | 29.16 |
| (C56−C57−C58) | 102.97 | 103.39 | 103.92 | 103.62 | (C56−C57−C58−C59) | −35.12 | −39.96 | −38.30 | −39.44 |
| (C56−C57−H63) | 111.21 | 110.92 | 110.33 | 109.85 | (C56−C57−C58−H65) | −28.24 | 77.64 | 79.43 | 78.24 |
| (C56−C57−H64) | 111.30 | 110.19 | 110.74 | 110.96 | (C56−C57−C58−H66) | 154.73 | −160.53 | −159.20 | −160.25 |
| (C58−C57−H63) | 111.15 | 113.63 | 113.46 | 113.68 | (H63−C57−C58−C59) | −154.26 | −159.39 | −158.14 | −158.63 |
| (C58−C57−H64) | 111.13 | 110.25 | 110.35 | 110.45 | (H63−C57−C58−H65) | 131.16 | −41.79 | −40.41 | −40.96 |
| (H63−C57−H64) | 109.00 | 108.41 | 108.04 | 108.26 | (H63−C57−C58−H66) | 86.12 | 80.04 | 80.96 | 80.56 |
| (C57−C58−C59) | 104.66 | 103.02 | 103.28 | 103.76 | (H64−C57−C58−C59) | 84.15 | 78.67 | 80.46 | 79.45 |
| (C57−C58−H65) | 110.81 | 110.48 | 110.50 | 110.51 | (H64−C57−C58−H65) | 15.75 | −163.73 | −161.81 | −162.87 |
| (C57−C58−H66) | 110.83 | 113.48 | 113.21 | 113.34 | (H64−C57−C58−H66) | −35.47 | −41.90 | −40.44 | −41.35 |
| (C59−C58−H65) | 110.81 | 110.13 | 110.10 | 109.83 | (C57−C58−C59−N54) | 15.01 | 35.81 | 36.86 | 35.13 |
| (C59−C58−H66) | 110.91 | 111.39 | 111.64 | 111.26 | (C57−C58−C59−H67) | 134.64 | 155.31 | 156.07 | 154.81 |
| (H65−C58−H66) | 108.80 | 108.29 | 108.06 | 108.10 | (C57−C58−C59−H68) | 28.75 | −80.24 | −79.36 | −80.32 |
| (N54−C59−C58) | 104.94 | 103.81 | 103.46 | 103.45 | (H65−C58−C59−N54) | −104.48 | −82.04 | −81.15 | −83.03 |
| (N54−C59−H67) | 110.85 | 109.53 | 109.52 | 109.93 | (H65−C58−C59−H67) | 15.16 | 37.46 | 38.06 | 36.66 |
| (N54−C59−H68) | 110.71 | 108.12 | 108.32 | 107.73 | (H65−C58−C59−H68) | 24.70 | 161.91 | 162.63 | 161.53 |
| (C58−C59−H67) | 110.70 | 114.75 | 114.67 | 114.63 | (H66−C58−C59−N54) | 134.57 | 157.80 | 158.82 | 157.33 |
| (C58−C59−H68) | 110.79 | 111.25 | 111.52 | 111.36 | (H66−C58−C59−H67) | −105.79 | −82.71 | −81.97 | −80.89 |
| (H67−C59−H68) | 108.84 | 109.09 | 109.06 | 109.40 | (H66−C58−C59−H68) | 15.05 | 41.75 | 42.60 | 41.28 |

**Table S2** Theoretical and experimental vibrational wavenumber (cm−1) of EZT with potential energy distribution.

| **Scaled Wavenumber** | **Experimental** | | **Potential Energy Distribution (≥5%)** |
| --- | --- | --- | --- |
|  | **IR** | **Raman** |  |
| 3710 | 3434 |  | R1[(OH](100) |
| 3700 |  |  | [O3H](100) |
| 3115 | 3074 |  | R4[(CH)](99) |
| 3104 |  |  | R4[(CH)](99) |
| 3098 |  |  | R3[(CH)](98) |
| 3097 | 3057 | 3060 | R3[(CH)](98) |
| 3095 |  |  | R4[(CH)](95) |
| 3092 |  |  | R1[(CH)](99) |
| 3091 |  |  | R4[(CH)](98) |
| 3076 | 3047 |  | R1[(CH)](99) |
| 3070 |  |  | R3[(CH)](99) |
| 3063 |  |  | R3[(CH)](99) |
| 3055 |  |  | R1[(CH)](99) |
| 3051 | 3016 | 3017 | R1[(CH)](99) |
| 2986 |  |  | as[C24H2](79)+R2[(C27H)](18) |
| 2976 |  |  | R2[(C27H)](54)[C19H](26)+as[C24H2](20) |
| 2967 |  |  | [C19H](55)+R2[(C27H)](26)as[C24H2(15) |
| 2947 |  |  | [C21H2](77)[C19H](17) |
| 2932 | 2929 | 2935 | R2[(C29H)](63)[C24H2](26)s[C21H2(6) |
| 2928 |  |  | s[C24H2](49)+R2[(C29H)](36)s[C21H2](12) |
| 2917 |  |  | [C21H2](79)[C24H2](19) |
| 1758 | 1728 | 1729 | R2[(C=O)](80)+R2[(C32N)](8)+R2[(C27C32)](6)+R2[](5) |
| 1601 | 1616 |  | R1[(CC)](60)+R1[in(CH)](19)+R1[a](11) |
| 1595 |  | 1611 | R4[(CC)](63)+R4[in(CH)](19)+R4[a](11) |
| 1592 | 1594 |  | R3[(CC)](64)+R3[a](11)+R3[in(CH)](20) |
| 1581 |  |  | R3[(C)](73)+R3[’a](9) |
| 1579 |  |  | R1[(CC)](61)+R1[’a](8)+R1[in(C40H)](5) |
| 1579 |  |  | R4[(CC)](68)+R4[’a](8) |
| 1495 | 1510 | 1510 | R1[in(CH)](49)+R1[(CC)](35)+R1[(CO)](8)+  R2[(C29C31)](5) |
| 1490 |  |  | R3[in(CH)](47)+R3[(CC)](38)+R3[(CF)](6) |
| 1489 |  |  | R4[in(C4H)](43)+R4[(CC)](32)+R4[(C42N)](9)+  R4[(CF)](5) |
| 1435 |  | 1468 | sci[C24H2](81)sci[C21H2](10) |
| 1431 |  |  | sci[C21H2](81)+sci[C24H2](9) |
| 1424 | 1447 |  | R1[in(CH)](37)+R1[(CC)](29)+R2[(C29C31H](10)+  R1[in(OH)](7)+R2[in(C29C31](5)+R1[in(CO)](5) |
| 1408 | 1429 | 1430 | R4[in(CH)](34)+R4[(CC)](32)+R4[in(C42N)](8) |
| 1406 |  |  | R3[(CC)](24)+R3[in(CH)](24)+[C19OH](13)+  γ[C19OH](7)sci[C19OH](6) |
| 1369 | 1400 | 1400 | sci[C19OH](48)in[O3H](15)ρ[C19OH](6)+  R3[(C8C12)](5) |
| 1366 | 1367 | 1358 | [C21H2](14)[C24H2](13)+R4[(C42N)](12)+  R2[(C24C27H)](7)+R2[(C32N)](7)[C24C27](5)+  γ[C24H2](5) |
| 1353 | 1354 |  | [C21H2](18)+[C19OH](13)+sci[C19OH](9)+  R4[(C42N)](8)+γ[C19OH](6)+[C19C21](5)+ R2[(C32N)](5) |
| 1336 |  | 1340 | [C24H2](26)+R2[(C29C31H](12) |
| 1332 |  |  | R2[(C29C31H](22)+R4[(C42N)](12)+[C24H2](10)+  R2[(C29C31H](9)+R1[in(CH)](7)+R2[(C32N)](6) |
| 1320 |  |  | R1[in(CH)](45)+R1[(CC)](40)+R1[in(OH)](14) |
| 1316 |  |  | γ[C24H2](17)+[C21H2](14)+γ[C21H2](9)+  [C19OH](6)+γ[C19OH](5) |
| 1303 | 1304 |  | R3[(CC)](39)+R2[(C24C27H)](8)+[C21H2](6) |
| 1291 | 1293 |  | R4[(CC)](48)+R4[in(CH)](10) |
| 1282 |  | 1301 | R4[in(CH)](58)+R4[(CC)](19) |
| 1282 |  |  | R3[in(CH)](39)+R3[(C)](32) |
| 1278 |  |  | R3[in(CH)](20)+R2[(C24C27H)](9)+[C24H2](5)+  in[O3H](5) |
| 1268 | 1271 | 1272 | R1[(CC)](31)+R1[in(CH)](14)+R2[(C29C31H](9)+  R2[(C29C31)](7)+ R2[(C29C31H](5) |
| 1241 |  |  | R1[(CO)](51)+R1[(CC)](12)+R1[in(CH)](11)+  R1[(C38C40)](9)+R1[ri](9) |
| 1228 |  |  | R2[(C29C31H](27)+R2[γ(C29C31H](16)+γ[C24H2](10)+  [C21H2](5) |
| 1220 | 1217 | 1216 | R3[(CC)](26)+γ[C21H2](16)[C19OH](12)+in[O3H](7) |
| 1203 | 1202 | 1205 | R3[(CF)](46)+R3[(CC)](24)+R3[in(CH)](10)+R3[tri](6) |
| 1198 |  |  | R4[(CF)](43)+R4[in(CH)](17)+R4[(CC)](14)+R4[tri](12) |
| 1195 |  |  | R2[(C29C31)](15)+R2[(C24C27H](8)+R1[ri](5) |
| 1188 |  |  | R3[(CC)](12)+in[O3H](9)+R2[(C29C31)](9)+  R3[in(C15H)](7)+sci[C19OH](6+R3[tri](5) |
| 1181 | 1184 |  | in[O3H](13)+R2[(C24C27H)](12)+γ[C24H2](10)+  γ[C21H2](6)+R2[(C29C31H](5)+[C21H2](5)+  R2[ρ(C24C27H](5) |
| 1174 | 1170 |  | R2[(C24C27H](26)+R3[(C14C19)](13)+R2[γ(C24C27H](10) |
| 1157 | 1155 | 1158 | R1[in(CH)](72)+R1[(CC)](12)+R1[in(OH)](5) |
| 1150 |  |  | R1[in(OH)](46)+R1[(CC)](25)+R1[in(C38H)](11)+  R1[(CO)](6) |
| 1141 | 1140 |  | R3[in(CH)](68)+R3[(CF)](8) |
| 1140 |  |  | R4[in(C45H)](69)+R4[(CF)](8) |
| 1132 |  |  | γ[C21H2](14)+R2[γ(C24C27H](13)+  R2[(C24C27H](12)+in[O3H](11)+γ[C24H2](8) |
| 1117 | 1116 | 1117 | R2[(C29C31H](17)+R2[(CN)](25)+  R2[γ(C29C31H](9)++γ[C24H2](5)+R4[in(C50H)](5) |
| 1097 |  | 1103 | R1[in(CH)](31)+R1[(CC)](14)+R2[oop(C=O)](6)+  R1[in(C33H)](5)+R2[(C27C32)](5) |
| 1089 |  |  | R4[in(CH)](42)+R4[(CC)](16)+R1[in(CH)](17)+  R1[(C38C40)](5) |
| 1086 |  |  | R3[in(CH)](47)+R3[(CC)](25) |
| 1076 |  |  | [C24C27](33)+[C21C24](14)+R2[(C29C31H](5) |
| 1057 | 1063 | 1077 | R2[(C29N)](26)+R4[(CC)](14)+R2[(C27C29)](5)+  [C24C27](5) |
| 1049 |  |  | [C19C21](33)+[C21C24](15)+[CO3](9)+  ρ[C21H2](6)+R2[(C29N)](5) |
| 1035 |  |  | [CO3](21)+ρ[C24H2](14)ρ[C21H2](7)+[C19C21](6)+  R2[(C27C29)](5)+in[O3H](5)sci[C19CC](5) |
| 998 | 1008 | 1011 | R3[tri](48)+R3[(CC)](33)+R3[in(CH)](11) |
| 995 |  |  | R4[tri](43)+R4[(CC)](38)+R4[in(CH)](13) |
| 994 | 983 |  | R1[tri](49)+R1[(CC)](35)+R1[in(CH)](9) |
| 978 |  |  | [CO3](18)+R2[(CC)](13)ρ[C21H2](8)+R4[ri](6)+  [C21C24](5) |
| 947 |  | 949 | R4[oop(CH)](88)+R4[’a](6)+R4[puck](5) |
| 944 | 937 |  | R3[oop[CH)](80)+R3[’a](7) |
| 943 |  |  | R1[oop(CH)](33)+[CO3](9)+[C21C24](8)+  R2[(C27C29)](6)+R3[oop[C8H)](5) |
| 942 |  | 934 | R1[oop(CH)](82)+R1[’a](8) |
| 926 |  |  | R3[oop[CH)](56)+R3[puck](15)R1[oop(CH)](14) |
| 921 |  |  | R1[oop(CH)](53)+R3[oop[CH)](14)+R1[puck](12)+  [CO3](5) |
| 913 |  |  | R4[oop(CH)](75)+R4[puck](17) |
| 903 |  |  | [C21C24](14)+R2[(C27C29)](11)[C19C21](8) |
| 881 |  |  | [CO3](14)+R2[(C27C29)](14)[C19C21](7)+  ρ[C24H2](6)+R2[](5) |
| 842 | 852 | 860 | R3[(CC)](20)ρ[C21H2](8)+R3[(C14C19)](7)+  R3[a](7) |
| 834 |  |  | R1[oop(CH)](19)+R1[a](7)+R1[(CC)](14) |
| 827 | 829 | 824 | R4[oop(CH)](66)+R4[a](11)+R4[oop(CF)](8)+  R4[oop(C42N)](7) |
| 826 |  |  | R3[oop[CH)](60)+R3[a](11)+R3[oop[CF)](9)+  R3[oop(C14C19)](5) |
| 821 |  |  | R1[oop(CH)](44)+R1[oop(CO)](7)+R1[a](7) |
| 807 |  |  | R4[a](18)+R4[(CC)](24)+R4[(CF)](16)+R1[oop(CH)](14) |
| 803 |  |  | R3[oop[CH)](98) |
| 799 |  | 798 | R1[oop(CH)](34)+R2[](7) |
| 795 |  |  | R1[oop(CH)](58)+R4[oop(CH)](10) |
| 793 |  |  | R4[oop(CH)](81)+R1[oop(CH)](6) |
| 778 | 775 | 776 | R1[tri](9)+R3[(CF)](7)+R3[tri](7)+R1[(CO)](6)+  ρ[C24H2](5) |
| 760 |  |  | R1[puck](8)+R2[](7)+R2[(C29C31H](7)+R1[tri](5)+  R2[γ(C29C31H](5)+[C24C27](5) |
| 721 | 735 | 735 | R3[puck](43)+R3[oop[CF)](9)+R3[oop(C14C19)](8)+  R2[oop(C=O)](6)+R2[oop(C=O)](5) |
| 714 |  |  | R1[puck](40)+R1[oop(CO)](8)+R2[oop(C29C31)](8)+  ρ[C24H2](6)+R3[puck](6) |
| 710 |  |  | R3[puck](47)+R3[oop[CF)](9)+R3[oop(C14C19)](8) |
| 696 |  |  | R1[puck](44)ρ[C24H2](9)+R1[oop(CO)](8)+  ρ[C21H2](6)+R2[oop(C29C31)](5) |
| 695 |  |  | R4[puck](66)+R4[oop(C42N)](15)+R4[oop(CF)](14) |
| 630 | 636 | 636 | R1[’a](48)+R3[’a](26) |
| 629 |  |  | R3[’a](42)+R1[’a](32) |
| 626 |  |  | R4[’a](58)+R3[’a](6)+R2[oop(C=O)](6)+  R4[in(CF)](5) |
| 615 | 592 | 593 | R2[oop(C=O)](21)+R4[’a](14)+R2[(C27C32)](5)+  R2[puck](5) |
| 578 | 569 | 571 | R4[a](18)+R2[](17)+R1[a](6)+R4[(CF)](5)+  R4[(C42N)](5) |
| 553 | 555 | 556 | R3[a](16)+R3[oop[CF)](15)+R3[a](12)+  ρ[C19OH](7)+R3[puck](6)+R3[oop(C14C19)](6) |
| 540 | 540 | 537 | R1[a](29)+R1[oop(CO)](8)+R2[(C29C31H](8)+  R2[oop(C=O)](7)+R1[a](6) |
| 532 |  |  | R3[oop[CF)](21)+R3[a](19)+R3[oop(C14C19)](9)+  R3[a](9)sci[C19CC](7) |
| 516 | 519 |  | R1[oop(CO)](26)+R1[a](24)+R2[oop(C29C31)](13) |
| 509 |  |  | R4[a](31)+R4[oop(CF)](30)+R4[oop(C42N)](25) |
| 492 |  |  | ρ[C19OH](14)+sci[C21CC](10)+R3[in(C14C19)](9)+  γ[C19OH](8)ρ[C21H2](5) |
| 434 | 430 | 451 | R4[in(CF)](16)+R4[in(C42N)](11)+R4[’a](8)+  sci[C24CC](8) |
| 415 | 418 | 419 | R3[oop[CF)](19)+R3[puck](18)+R3[’a](12)+  R3[oop[CH)](8) sci[C19CC](7)+R3[a](7) |
| 412 |  |  | R4[’a](81)+R4[oop(CH)](17) |
| 412 |  |  | R3[’a](78)+R3[oop[CH)](11) |
| 410 |  |  | R1[’a](73)+R1[oop(CH)](11) |
| 409 |  |  | R1[in(CO)](37)+R4[in(CF)](17)+R1[’a](10)+  R4[’a](5) |
| 401 |  |  | R1[’a](25)+R1[in(CO)](11)+R1[puck](8)+  R1[oop(CH)](7)+R4[in(CF)](5)+R4[a](5)+  R1[oop(CO)](5) |
| 397 |  | 400 | R3[in(CF)](55)+R3[’a](11)+ρ[C19OH](5) |
| 383 |  | 371 | R4[oop(CF)](21)+R4[puck](20)+R2[oop(CN)](11)+  R4[oop(C42N)](8) |
| 366 |  | 356 | sci[C24CC](13)+R3[a](10)+R2[(C27C32)](7)+  sci[C21CC](6)+R3[(C14C19)](5)+R4[in(CF)](5)+  R2[oop(C=O)](5) |
| 337 |  | 330 | R4[in(CF)](13)+R2[in(CN](9)+R1[a](9)+  R4[in(C42N)](7)+R2[(C29C31)](6)+  R1[puck](6) |
| 318 |  |  | R2[ρ(C24C27H](19)+sci[C21CC](7)+[C21C24](6)+  R2[in(C29C31](5)+R4[oop(C42N)](5)+ρ[C24H2](5) |
| 314 |  | 307 | R1[(C3O)](91) |
| 282 |  | 298 | R4[a](11)+R1[a](7)+R2[oop(C29C31)](7)+  R4[(C42N)](6)+R1[puck](5)+R2[oop(C=O)](5) |
| 273 |  | 273 | γ[C19OH](12)[C19OH](9)+R3[in(C14C19)](8)+  sci[C21CC](6)+R3[oop(C14C19)](5)+R4[a](5) |
| 260 |  |  | R4[in(C42N)](14)+R1[a](11)+R2[(C29C31)](10)+  R2[in(CN](8)+ R2[(C29N)](6) |
| 232 |  |  | R3[a](23)+R3[oop(C14C19)](13)+  R3[in(C14C19)](10)+[CO3](8)+ρ[C19OH](7)+  sci[C19CC](6) |
| 226 |  | 238 | R2[oop(CN)](15)+R4[a](13)sci[C21CC](6)+  R2[in(C29C31](5)+R2[γ(C29C31H](5)+R1[a](5) |
| 205 |  |  | R2[in(C29C31](25)+R2[puck](16)+R2[oop(CN)](10)+  sci[C21CC](6)+R4[a](5) |
| 180 |  |  | [CO3](70)sci[C19OH](7) |
| 179 |  | 167 | R1[a](13)+[CO3](11)+sci[C19CC](10)+R3[a](9)+  R4[in(C42N)](6)sci[C21CC](5) |
| 145 |  | 151 | R1[a](14)+R3[in(C14C19)](13)+R3[a](10)sci[C19CC](6)+  sci[C24CC](5)+R4[a](5) |
| 126 |  | 126 | R1[a](19)+sci[C24CC](11)+R2[γ(C24C27H](10)+  R2[(C24C27H](7)+R3[in(C14C19)](6)+  R2[in(CN](5) |
| 114 |  |  | R4[a](36)+R2[ρ(C24C27H](9)+R2[in(C29C31](6) |
| 103 |  |  | R2[in(CN](16)+R3[a](12)+[C19C21](8)+  R2[γ(C24C27H](8)+R4[in(C42N)](8)+  R3[oop(C14C19)](7)sci[C24CC](6)+[C24C27](5) |
| 77 |  |  | [C21C24](11)+R2[in(CN](8)+R3[a](8)+  R3[oop(C14C19)](8)+[C14C19](7) |
| 70 |  |  | R2[puck](35)+R2[oop(CN)](18)+R2[(C29C31H](9)+  R4[(C42N)](9) |
| 59 |  |  | sci[C19CC](12)+R2[oop(C29C31)](9)+  sci[C21CC](9)+R2[oop(CN)](9)+R3[a](7)+  R3[oop(C14C19)](7)+R2[γ(C29C31H](5)+  [C21C24](5) |
| 41 |  |  | R4[(C42N)](50)+R1[(C29C31)](35) |
| 38 |  |  | R1[(C29C31)](50)+R4[(C42N)](24)+  R2[oop(C29C31)](7) |
| 30 |  |  | [C14C19](50)sci[C19CC](10)+  R3[oop(C14C19)](9)+R2[oop(CN)](5) |
| 27 |  |  | R2[oop(CN)](27)+[C14C19](15)+R2[puck](8)+  R2[oop(C29C31)](8)+  sci[C19CC](6)+R3[oop(C14C19)](5) |
| 21 |  |  | [C19C21](47)+[C21C24](29) |
| 15 |  |  | R2[puck](28)+R2[ρ(C24C27H](23)+[C14C19](8)+  R2[oop(CN)](6)+[C24C27](5)+R2[γ(C24C27H](5) |
| 8 |  |  | [C24C27](48)+[C21C24](22)+[C19C21](7) |

**Table S3** Theoretical and experimental vibrational wavenumber (cm−1) of L-proline with potential energy distribution.

| **Scaled Wavenumber** | **Experimental** | | **Potential Energy Distribution (≥5%)** |
| --- | --- | --- | --- |
|  | **IR** | **Raman** |  |
| 3391 | 3183 |  | R5[as(NH2+)](100) |
| 3322 | 3134 |  | R5[s(NH2+)](100) |
| 3000 | 3053 |  | R5[as(CH2)](93) |
| 2991 | 3006 | 3006 | R5[as(CH2)](90) |
| 2985 |  |  | R5[(CH)](91)+R5[as(CH2)](5) |
| 2980 | 2981 | 2983 | R5[s(CH2)](92) |
| 2945 | 2952 | 2950 | R5[s(CH2)](95) |
| 2938 |  | 2933 | R5[s(CH2)](96) |
| 2907 | 2875 | 2874 | R5[s(CH2)](97) |
| 1729 | 1613 | 1625 | R5[s(COO−)](88)+R5[δsci(C1CO)](8) |
| 1549 | 1554 | 1549 | R5[δ(NH2+)](87)+R5[ω(NH2+)](5) |
| 1489 |  |  | R5[δ(CH2)](93) |
| 1478 | 1473 | 1472 | R5[δ(CH2)](97) |
| 1471 | 1448 | 1453 | R5[δ(CH2)](97) |
| 1368 | 1375 | 1375 | R5[ω(CH2)](68)+R5[(C4C5)](7)+R5[ω(NH2+)](7)  +R5[γ(NH2+)](6) |
| 1342 |  | 1349 | R5CH2)](52)+R5[γ(CH2)](26)+R5[(C3C4)](8) |
| 1332 |  |  | R5[δ(NC2C3)](19)+R5[ω(CH2)](27)+R5[ω(NH2+)](8)  +R5[(CO16)](8)+R5[(C2C)](6)+R5[γ(C4H2)](6)  +R5[γ(NH2+)](5) |
| 1310 | 1319 | 1316 | R5CH2)](38)+R5[δ(NC2C3)](20)+  [γ(NH2+)](11)+R5[(CO16)](9) |
| 1301 | 1292 | 1286 | R5[(COO−)](33)+R5CH2)](28)+  R5[γ(C5H2)](11)+R5[oop(C2H)](5)+  R5[ω(NH2+)](5)+R5[δ(NC2C3)](5) |
| 1272 |  |  | R5[(CO16)](14)+R5[γ(C5H2)](12)+ R5C3H2)](16)+R5[(CC)]+(17)+R5[oop(C2H)](11)+  C3H2)](6)+R5[ω(C2NC)](5) |
| 1263 |  | 1266 | R5[oop(C2H)](31)+R1[γ(C4CN)]( 29)+R5[γ(C4C)](7)+  R5[ρ(C3H2)](6) |
| 1248 | 1253 | 1238 | R5[δ(NC2C3)](23)+R5[γ(NH2)](18)+R5C3H2)](9)+  R5[ρ(CH2)](16)+R5[ω(C5H2)](6) |
| 1197 |  |  | R5[ω(NH2+)]( 48)+R5[γ(C4H2)](16)+R5[ω(C4H2)](5)  +R5[(C4C5)](5) |
| 1177 | 1168 | 1175 | R5CH2)](30)+R5[(CC)](21)+R5[ω(C4H2)](8)+  R5[ω(NH2+)](6)+R5[δ(NC2C3)](6)+R5[ρ(C5H2)](5) |
| 1122 |  |  | R5[oop(C2H)](32)+R5[γ(NH2+)](27)+R5[γ(C4H2)](8) |
| 1080 | 1083 | 1083 | R5[ρ(CH2)](36)+R5C3H2)](18)+R5[(NC2C3)](10)+  R5[(C2C)](8)+R5[(C2N)](5) |
| 1030 | 1033 | 1033 | R5[(CC)](45)+R5[ring](10)+R5[γ(NH2+)](6)+  R5CH2)](10)+R5[ρ(C3H2)](5)+R5[(C5N)](5) |
| 1006 | 981 | 985 | R5[(CN)](32)+R5[ρ(C4H2)](14)+R5[(CC)](20)+  R5['ring](11)+R5[γ(NH2+)](6)+R5[ring](6) |
| 949 | 946 | 951 | R5[ρ(CH2)](27)+R5[oop(C2C1)](13)+R5[γ(CH2)](24)+  R5[ρ(NH2+)](10)+R5[(C2C)](6) |
| 929 | 914 | 920 | R5[(CC)](47)+R5[(C5N)](16)+R5[γ(C4H2)](8)+  R5[ρ(NH2+)](6) |
| 904 | 902 | 898 | R5[(CC)](76)+R5[(C5N)](7) |
| 884 | 883 | 866 | R5[(CN)](35)+R5[ρ(CH2)](21) |
| 840 | 846 | 842 | R5[(CN)](27)+R5[ρ(CH2)](33)+R5[δsci(C1CO)](8)  +R5[(C3C4)](6) |
| 815 |  |  | R5[(C1C)](26)+R5[δsci(C1CO)](17)+R5[ρ(C1CO)](17)+  R5[(C2N)](9)+R5[ρ(C3H2)](8) |
| 787 | 786 | 792 | R5[(C2N)](41)+R5[ρ(C1CO)]( 19)+R5[ρ(NH2+)](11) |
| 761 |  |  | R5[δoop(CC)](47)+R5[ρ(NH2+)](28)+R5[oop(C2H)](10)  +R5[(NC2C3)](5) |
| 652 | 640 | 642 | R5[ring](44)+R5['ring](9)+R5[ρ(C5H2)](13)+  R5[δoop(C1C)](7)+R5[ρ(C1CO)](6)+R5[ρ(NH2+)](5) |
| 603 |  |  | R5[(CC)](37)+R5[δsci(C1CO)](29)+R1[δoop(C1C)](21) |
| 576 | 570 | 573 | R5['ring](42)+R1[ρ(CH2)](33)+R5[ring](7) |
| 465 | 449 | 448 | R5[δsci(C1CO)]( 18)+R5[(NC2C3)](17)+R5[(C1C)](16)  +R5[ρ(C1CO)](16) +R5[(C2N)](8)+R5[ring](6)  +R5[δoop(C1C)](5) |
| 317 |  | 296 | R5[oop(C2C1)](42)+R5[oop(C2H)](7)+R5['ring](7)+  R5[ring](6)+R5[δoop(C1C)](6)+R5[ρ(NH2+)](5) |
| 274 |  |  | R5[(NC2C3)](27)+R5[ρ(C1CO)](15)+R5[δsci(C1CO)](13)  +R5[ρ(NH2+)](9)+R5[ring](8)+R5[(NH2+)](7) |
| 185 |  | 160 | R5[ring](33)+R5[oop(C2C1)](27)+R5[(NC2C3)](22) |
| 141 |  | 136 | R5[ρ(C2NC)]( 30)+R5[ring](21)+R5['ring](17)+  R5[oop(C2C1)](8)+R5[(NH2+)](5) |
| 120 |  | 122 | R5[(C1C)](48)+R5['ring](12)+R5[oop(C2C1)](12)+  R5[δ(NH2+)](6)+R5[(NC2C3)](6)+R5[ω(NH2+)](5) |

**Table S4** Theoretical and experimental vibrational wavenumber (cm−1) of EZT+L-prolinewith potential energy distribution.

| **Scaled wavenumber** | **Experimental** | | **Potential energy distribution (≥5%)** |
| --- | --- | --- | --- |
|  | **IR** | **Raman** |  |
| 3711 | 3471 |  | R1[(OH)](100) |
| 3434 | 3128 |  | [(O4H)](96) |
| 3366 | 3075 | 3077 | R5[as(H2+)](99) |
| 3115 | 3053 |  | R4[(CH)](99) |
| 3103 |  |  | R4[(CH)](99) |
| 3095 |  |  | R3[(CH)](99) |
| 3094 |  |  | R4[(CH)](96) |
| 3092 |  |  | R1[(CH)](99) |
| 3091 |  |  | R3[(CH)](98) |
| 3089 |  |  | R4[(CH)](99) |
| 3083 |  |  | R3[(CH)](98) |
| 3077 |  |  | R1[(CH)](99) |
| 3061 |  | 3061 | R3[(CH)](99) |
| 3057 |  |  | R1[(CH)](97) |
| 3051 |  |  | R1[(CH)](97) |
| 3028 | 3031 | 3036 | R5[as(CH2)](98) |
| 3024 |  | 3028 | R5[(C56H)](69)+R5[as(CH2)](28) |
| 3020 |  |  | R5[as(CH2)](70)+R5[(C56H)](27) |
| 3010 | 3011 | 3007 | R5[as(CH2)](99) |
| 2993 |  | 2984 | [as(C15H2)](91)+[as(C16H2)](8) |
| 2970 | 2973 | 2978 | R5[s(CH2)](98) |
| 2967 |  |  | R2[(C14H)](83)+[s(C16H2)](14) |
| 2963 |  |  | [s(C16H2)](75)+R2[(C14H)](13)+[s(C15H2)](10) |
| 2962 |  |  | R5[s(CH2)](97) |
| 2943 | 2942 | 2947 | R5[(CH2)](99) |
| 2941 |  |  | R2[(C24H)](94) |
| 2923 | 2935 | 2933 | [s(C16H2)](73)+[s(C15H2)](25) |
| 2918 | 2902 | 2903 | [s(C15H2)](72)+[s(C16H2)](26) |
| 2876 | 2854 | 2855 | [(C17H)](98) |
| 2492 | 2741 | 2576 | R5[s(H2+)](96) |
| 1755 | 1736 | 1727 | R2[(C=O)](80)+R2[(C13)](7)+R2[(C13C14)](6)+R2[](5) |
| 1712 | 1605 | 1611 | R5[(COO−)](85)+R5[C55CO)](7) |
| 1601 |  |  | R1[(CC)](60)+R1[a](11)+R1[in(CH](19) |
| 1594 |  |  | R4[(CC)](63)+R4[a](11)+R4[in(CH)](19) |
| 1584 |  | 1588 | R5[(H2+)](33)+R5[(H2+)](27)+R3[(CC)](19) |
| 1582 |  |  | R5[(H2+)](40)+R5[(H2+)](33)+R3[(CC)](15) |
| 1579 |  |  | R1[(CC)](60)+R1[’a](7)+R1[in(C26H)](5) |
| 1578 | 1578 |  | R4[(CC)](67)+R4[a’](8) |
| 1576 |  |  | R3[(CC)](66)+R3[’a](8)+R5[(H2+)](5)+R5[(H2+)](5) |
| 1495 | 1509 | 1511 | R1[in(CH](49)+R1[(CC)](35)+R1[(OH)](8)+R2[(C24C25)](5) |
| 1489 |  |  | R4[in(CH)](47)+R4[(CC)](41)+R4[(CF)](6) |
| 1481 |  |  | R3[in(CH)](52)+R3[(CC)](37)+R3[(CF)](7) |
| 1469 |  | 1475 | R5[(CH2)](94) |
| 1455 |  |  | R5[(CH2)](97) |
| 1449 | 1449 | 1451 | R5[(CH2)](96) |
| 1445 |  |  | [sci(C15H2)](67)+[sci(C16H2)](27) |
| 1431 | 1437 | 1441 | [sci(C16H2)](67)[sci(C15H2)](27) |
| 1423 |  | 1426 | R1[(CC)](35)+R1[in(CH)](24)+R2[(C24C25H)](11)+  R1[in(OH](7)+R1[in(C24C25)](5)+R1[in(CO](5) |
| 1410 |  |  | [in(O4H)](59)+[sci(C17OH)](20)+[(C16C17)](6) |
| 1408 |  |  | R4[(CC)](42)+R4[in(CH)](35)+R4[in(C)](8) |
| 1396 | 1386 | 1389 | R3[(CC)](30)+R3[in(CH)](24)+[(C17OH)](11)+[(C17OH)](7) |
| 1362 |  |  | R5[(C59H2)](44)+R5[(H2+)](23)+R5[(C58C59)](6) |
| 1360 |  |  | [(C15H2)](32)+[(C16H2](27)+[(C15C16)](9)+  R2[(C14C15H)](6) |
| 1355 | 1352 | 1353 | R4[(C7)](25)+R2[(C)](20)+R2[(C24C25H)](8) |
| 1335 |  |  | [sci(C17OH)](16)+R3[(C22C23)](14)+[(C17OH)](12)+  [(C15H2)](7)+[(C17OH)](5)+R3[in(C20H)](5)+  [C17OH)](5) |
| 1331 |  |  | R2[(C24C25H)](27)+R2[C24C25H)](9)+R4[(C7)](8)+  R1[in(CH)](11) |
| 1320 | 1326 | 1324 | R1[(CC)](45)+R1[in(C26H)](39)+R1[in(OH](13) |
| 1318 |  |  | R5[(CH2)](50)+R5[γ(CH2)](23)+R5[(C57C58)](8)+  R5[in(C56H)](5) |
| 1317 |  |  | [γ(C16H2)](19)+[sci(C17OH)](7)+[(C17OH)](7)+  [(C16H2)](7)+[in(O4H)](7)+[C17OH)](5)+[(C17OH)](5)+  [(C15H2)](5) |
| 1312 |  |  | R5[in(C56H)](18)+R5[oop(C56H)](12)+R5[γ(H2+)](12)+  R5[H2+)](12)+R5[γ(CH2)](9)R5[(H2+)](8)+R5[(C59H)](7) |
| 1304 | 1305 | 1307 | R5[(COO−)](28)+R5[(C59H2)](12)+R5[sci(C55CO)](7)+  R5[γ(H2+)](6)+R5[(H2+)](6)+R5[(H2+)](5)+  R5[oop(C56H)](5) |
| 1301 |  | 1298 | R2[(C14C15H)](15)+[(C16H2)](9)+[γ(C16H2)](5)+  R2[(C14C15)](5)+R2[C14C15H)](5) |
| 1291 |  | 1290 | R4[(CC)](51)+R4[in(CH)](11)+[γ(C15H2)](9) |
| 1289 |  |  | R5[(CH2)](64)+R5[in(C56H)](12)+R5[(H2)](7) |
| 1283 |  |  | [γ(C15H2)](20)+R3[(CC)](16)+[sci(C17OH)](14)+ R4[in(C11H)](11)+[(C17OH)](5) |
| 1282 | 1287 |  | R4[(CC)](42)+R4[in(CH)](40) |
| 1277 |  |  | R3[(CC)](41)+R3[in(C19H)](32) |
| 1271 | 1270 | 1271 | R3[in(CH)](25)+R1[(CC)](15)+R2[C24C25H)](6)+  R2[(C24C25)](6) |
| 1260 |  |  | R3[(CC)](13)+R1[(CC)](10)+[(C17OH)](7) |
| 1258 |  | 1258 | R5[oop(C56H)](20)+R5[γ(CH2)](17)+R5[(C55C)](16)+  R5[(COO−)](13)+R5[(H2+)](9)+R5[(C57H2)](5) |
| 1240 |  |  | R1[(OH)](50)+R1[(CC)](22)+R1[in(CH](10)+R1[ri](9) |
| 1237 |  |  | R5[γ(CH2)](57)+R5[in(C56H)](8)+R5[CH2)](9)+  R5[oop(C56H)](5) |
| 1232 |  |  | [(C16H2)](15)+[(C15H2)](11)+R2[(C24C25H)](11)+  R2[(C14C15)](7)+[(C17OH)](7)+R2[(γ(C24C25H)](7) |
| 1223 | 1223 | 1223 | R5[in(C56H)](29)+R5[(C59H2)](15)+R5[γ(C59H2)](10)+  R5[(H2+)](10)+R5[γ(H2+)](9)+R5[(C56C57)](6) |
| 1206 |  |  | R2[(C14C15H)](15)+[γ(C16H2)](10)+R2[(C24C25)](10) |
| 1199 | 1201 | 1205 | R4[(CF)](20)+R4[(CC)](10)+R2[(C14C15H)](7)+  R2[(C14C15H)](6)+R2[(C24C25H)](5) |
| 1195 |  | 1195 | R4[(CF)](23)+R4[in(C8H)](13)+R4[tri](11)+  R2[(C14C15H)](6)+R4[in(C12H)](5) |
| 1192 |  |  | R3[(CF)](22)+R5[(COO−)](9)+R5[(H2+)](5)+R3[(CC)](8)+  R5[(H2+)](5) |
| 1189 |  |  | R2[(C24C25)](22)+R1[(CC)](13)+R1[in(CH)](11)+  R2[(C24C25H)](9)+R1[tri](8)+R1[(C2930)](5) |
| 1188 |  |  | R3[(CF)](20)+R5[(COO−)](10)+R5[(H2+)](8)+  R5[γ(C58H2)](7)+R5[(H2+)](7)+R3[(CC)](7) |
| 1164 | 1165 | 1166 | [(C17C18)](38)+R3[(C18C19)](18)+R3[tri](14)+  R3[in(C19H)](6) |
| 1159 |  |  | R5[γ(C57H2)](33)+R5[(C58H2)](20)+R5[(CC)](14) |
| 1157 | 1154 | 1155 | R1[in(CH](74)+R1[(CC)](14) |
| 1149 | 1145 |  | R1[in(OH](48)+R1[(CC)](22)+R1[in(C27H)](10)+  R1[(OH)](6) |
| 1142 |  |  | R2[(C14C15H)](19)+R2[(γ(C14C15H)](15)+[γ(C15H2)](14)+  [γ(C16H2)](10)+[(C17OH)](5) |
| 1140 |  |  | R4[in(CH)](69)+R4[(CF)](8) |
| 1136 | 1126 | 1124 | R5[oop(C56H)](27)+R5[H2)](14)+R5[γ(CH2)](16)+  R5[C59H2)](6)+R5[γ(H2+)](5) |
| 1134 |  |  | R3[in(CH)](54)+R5[oop(C56H)](6)+R3[(CF)](5) |
| 1114 |  | 1106 | R2[(C)](30)+R2[(C24C25H)](21)+R2[(γ(C24C25H)](13)+  +R4[in(C8H)](5) |
| 1099 | 1095 | 1096 | R1[in(CH)](27)+R1[(CC)](12)+R2[(δin(C=O)](6)+  R2[(CC)](9)+R2[(C24C25H)](5) |
| 1090 |  |  | R4[in(CH)](23)+R4[(CC)](10)+[(CO4)](5)+R1[(C26C27)](5) |
| 1085 |  |  | [(CO4)](12)+R3[in(CH)](25)+R3[(C22C23)](12) |
| 1077 |  | 1079 | R2[(CC)](23)+[(C15C16)](12)+R4[in(C9H)](5) |
| 1065 |  |  | [(CO4)](22)+R3[in(CH)](15)+R3[(C19C20)](6) |
| 1063 | 1067 |  | R5[C57H2)](38)+R5[γ(C57H2)](16)+R5[in(CC)](14)+  R5[H2+)](5) |
| 1055 | 1057 | 1056 | [(CO4)](22)+R2[(C24)](18)+[(C16C17)](11)+R4[(CC)](9) |
| 1034 | 1030 |  | [(C16C17)](25)+[C15H2)](13)+[C16H2)](12)+R2[(C24)](8) |
| 1020 | 1014 | 1040 | R5[(CC)](43)+R5[](9)+R5[in(C56H)](8)+R5[(C57H2)](7) |
| 1007 |  | 1030 | [(C15C16)](35)+R2[(CC)](11)+R2[C14C15H)](10)+  [sci(C15CC)](8) |
| 1001 |  | 1013 | R5[(C)](35)+ R5[(CC)](18)+R5[’](16)+ R5[C58H2)](8) |
| 997 | 988 | 986 | R3[tri](46)+R3[(CC)](33)+R3[in(CH)](11) |
| 995 |  |  | R4[tri](42)+R4[(CC)](39)+R4[in(CH)](9)+R1[tri](5) |
| 995 |  |  | R1[tri](43)+R1[(CC)](25)+R4[tri](6)+R1[in(CH)](8) |
| 963 |  |  | R3[oop(CH)](84)+R3[’a](7) |
| 955 | 957 | 958 | R5[H2+)](17)+R5[oop(C55C)](9)+R5[γ(H2+)](9)+  R5[γ(C59H2)](15)+R5[(H2)](8)+R5[C57H2)](7) |
| 946 |  |  | R4[oop(CH)](88)+R4[a’](6)+R4[puck](5) |
| 942 |  |  | R1[oop(CH)](66)+R1[’a](6) |
| 941 |  |  | R1[oop(CH)](33)+[(CO4)](9)+[(C16C17)](8)+  R2[(C14C24)](8)+R1[oop(C29H)](8) |
| 929 |  | 927 | R3[oop(CH)](74)+R3[puck](16) |
| 923 | 923 |  | R5[(CC)](34)+R5[(C59)](19)+R5[C59H2)](13)+  R5[(C58H2)](6) |
| 921 |  |  | R1[oop(CH)](62)+R1[puck](14) |
| 912 | 910 | 911 | R4[oop(CH)](75)+R4[puck](17) |
| 887 | 893 | 895 | R2[(CC)](25)+R2[](9) R4[(CC)](6)+[C16H2)](5) |
| 883 |  |  | R5[(CC)](71)+R5[(C59)](11) |
| 878 | 871 |  | R2[(CC)](20)+[(C15C16)](5)+[(CO4)](5)+R2[(C14C15H)](5)+  [sci(C16CC)](5) |
| 876 | 861 | 862 | R5[(C)](28)+R5[(CC)](25)+R5[C57H2)](10)+R5[’](5)+  R5[γ(C57H2)](5) |
| 845 | 850 | 844 | R5[C59H2)](15)+R5[H2+)](14)+R5[γ(H2+)](14)+  R5[(C59)](9)+R5[(CC)](9) |
| 844 |  |  | R1[(CC)]22)+R1[a](6) |
| 832 | 833 | 833 | R3[oop(CH)](28)+R5[(C56)](19)+R5[CH2)](16)+R3[a](5) |
| 831 |  |  | R3[oop(CH)](41)+R5[(C56)](13)+R5[CH2)](10)+R3[a](7)+  R3[oop(CF)](5) |
| 827 | 828 |  | R4[oop(CH)](64)+R4[a](11)+R4[oop(CF)](8)+R4[oop(C)](8) |
| 824 |  | 825 | R3[(CC)](20)+R3[a](10)+[(C17C18)](6)+R3[(CF)](6)+  R1[oop(CH)](8) |
| 821 |  |  | R1[oop(CH)](43)+R1[a](7) |
| 812 | 816 |  | R3[oop(CH)](90) |
| 809 |  | 809 | R2[(CC)](13)+R1[oop(CH)](13)+R4[a](10)+R4[(CF)](9)+ +[C16H2)](6) |
| 802 |  |  | R5[sci(C55CO)](32)+R5[(CC)](23)+R5[C57H2)](8)+  R5[oop(C55C)](6)+R5[(C55CO)](5) |
| 798 |  | 800 | R1[oop(CH)](58)+R1[(OH)](6)+R1[tri](5) |
| 793 |  |  | R4[oop(CH)](41)+R1[oop(CH)](27) |
| 792 |  |  | R4[oop(CH)](55)+R1[oop(CH)](21) |
| 780 | 785 | 786 | [C16H2)](13)+R4[(CF)](9)+[C15H2)](9)+R2[](8)+  R4[tri](7)+R4[(CC)](6)+R4[a](5) |
| 768 |  |  | [C15H2)](7)+R3[tri](6)+R3[(CF)](6)+[C16H2)](5) |
| 762 | 763 | 763 | R5[(C55CO)](23)+R5[oop(C55C)](11)+R5[sci(C55CO)](9)+  R5[oop(C56H)](8)+R5[γ(H2+)](6)+R5[(C56)](5)+  R5[(C56C57)](5) |
| 749 | 732 |  | R3[tri](9)+R1[puck](7)+R3[(CF)](7)+R2[(C14C15)](6)+  [(C17C18)](6) |
| 719 | 712 | 717 | [(C17O)](71) |
| 708 |  |  | R3[puck](44)+R1[puck](18)+R3[oop(C17C18)](11)+  R3[oop(CF)](9) |
| 705 |  | 701 | R1[puck](40)+R3[puck](24)+R1[oop(CO)](8)+  R1[oop(C24C25)](6)+R3[oop(C17C18)](6)+R3[oop(CF)](5) |
| 695 |  |  | R4[puck](67)+R4[oop(C)](15)+R4[oop(CF)](14) |
| 690 |  |  | [C15H2)](24)+R2[(δin(C=O)](12)+[C16H2)](9)+  R2[(C14C24)](5)+[(C15C16)](5) |
| 646 | 668 | 666 | R5[](37)+R5[(H2+)](15)+R5[γ(H2)](8)+R5[(C55CO)](7)+  R5[C55CO)](5) |
| 629 | 645 | 635 | R1[’a](79)+R1[in(CO](5) |
| 626 |  |  | R3[’a](48)+R4[a’](26) |
| 626 |  |  | R4[a’](45)+R3[’a](29) |
| 615 | 615 | 617 | R5[(CC)](27)+R5[sci(C55CO)](13)+R5[C55CO)](11)+  R5[(C55CO)](10)+R5[oop(C55C)](9)+R5[γ(H2+)](8)+  R5[in(C55C)](5) |
| 603 | 593 | 593 | R2[(C13C14)](12)+R4[a](12)+R2[(δin(C=O)](8)+R4[(C7)](6)+  R4[a’](6)+R4[tri](6)+R2[(δin(C=O)](5)+R4[(CF)](5) |
| 578 | 570 | 572 | R2[](15)+R4[a](10)+R1[a](7)+R2[(γ(CCH)](9)+  R2[(δin(C=O)](5) |
| 560 | 561 | 560 | R5[’](46)+R5[CH2)](32) |
| 560 |  |  | [C17OH)](19)+R3[a](14)+[C16H2)](6)+[sci(C17CC)](5)+  R3[in(C17C18)](5)+R3[’a](5) |
| 552 |  |  | R3[oop(CF)](10)+R2[(δin(C=O)](9)+R3[a](9)+R1[a](7)+  R3[oop(C17C18)](7)+R3[a](7)+[(C17OH)](5) |
| 540 | 540 | 540 | R3[a](18)+R3[oop(CF)](18)+R3[oop(C17C18)](13)+R1[a](8)+  [sci(C17CC)](5) |
| 514 |  | 512 | R4[a](12)+R4[oop(CF)](12)+R4[oop(C)](11)+  R1[oop(CO)](9)+R1[a](8)+R3[a](7)+R3[oop(CF)](6)+  R1[oop(C24C25)](5) |
| 509 | 508 |  | R1[oop(CO)](13)+R1[a](13)+R1[oop(C24C25)](6)+  [C17OH)](5) |
| 504 |  |  | R4[a](21)+R4[oop(CF)](20)+R4[oop(C)](15)+  R1[oop(CO)](8)+R1[a](8) |
| 451 | 469 | 469 | R5[C55CO)](25)+R5[(C55C)](15)+R5[in(C55C)](14)+  R5[](10)+R5[(C56)](5)+R5[(C55CO)](5)+  R5[sci(C55inCO)](5) |
| 440 | 456 | 457 | [sci(C15CC)](11)+[sci(C17CC)](8)+R2[C14C15H)](7)+  R3[oop(CF)](5) |
| 418 | 429 | 446 | R4[in(CF)](28)+R1[in(CO](13)+R4[a’](10)+R4[in(C)](10)+  R2[(δin(C=O)](7) |
| 416 |  |  | R3[oop(CF)](12)+R3[puck](10)+R3[’a](9)+R3[a](6)+  [sci(C17CC)](5)+R3[in(CF)](5)+R4[(CF)](5) |
| 412 | 421 | 429 | R3[’a](74)+R3[oop(CH)](15) |
| 412 |  |  | R4[a’](78)+R4[oop(CH)](15) |
| 409 | 408 | 408 | R1[’a](77)+R1[oop(CH)](15) |
| 402 |  |  | R1[in(CO](37)+R1[’a](10)+R1[’a](9)+R4[in(CF)](6) |
| 398 |  | 396 | R3[in(CF)](23)+R1[puck](10)+R4[a](7)+R1[oop(CO)](6)+  R3[’a](5)+R4[in(CF)](5)+R2[(γ(C24C25H)](5) |
| 388 |  | 382 | R3[in(CF)](30)+R3[puck](8)+R3[oop(CF)](7)+R3[’a](6)+  [sci(C17CC)](5) |
| 367 |  | 359 | R4[puck](19)+R4[oop(CF)](19)+R4[oop(C)](12)+  R2[(δoop(C)](5)+R1[a](5) |
| 340 |  |  | R5[γ(H2+)](23)+R5[oop(C55C)](15)+R5[’](14)+  R5[C55CO)](11)+R5[H2+)](10)+R5[(H2+)](9) |
| 337 |  | 332 | R4[in(CF)](15)+R4[in(C)](8)+R2[(δin(C)](8)+R1[in(CO](6)+  R1[puck](5) |
| 311 |  |  | R3[a](12)+R3[oop(C17C18)](11)+[(C17OH)](10)+  [(O52H50)](9)+[(C17C18)](8)+[(C17OH)](6) |
| 309 |  |  | R1[(CO)](86) |
| 290 |  | 294 | R5[](29)+R5[in(C55C)](22)+R5[oop(C55C)](12)+  R5[C55CO)](7)+R5[’](6) |
| 286 |  | 275 | R4[a](13)+R1[a](9)+R1[oop(C24C25)](9)+R1[puck](8)+  R4[(C7)](7)+R2[(C)](9) |
| 265 |  | 259 | R4[in(C)](15)+R2[(δin(C)](9)+R1[a](7)+R2[(δin(C=O)](6)+  R2[(C24C25)](5)+R4[in(CF)](5) |
| 241 |  | 239 | R3[oop(C17C18)](13)+[sci(C16CC)](12)+[sci(C17CC)](11)+  R3[a](10)+[(C16C17OO52)](8)+[sci(C15CC)](7) |
| 229 |  | 229 | R2[(γ(CCH)](12)+R2[(δoop(C)](9)+R4[a](8)+  [(δin(C=O)](6)+R2[(C14C15H)](6)+R1[in(C24C25)](5) |
| 209 |  |  | [(O52H50)](9)+[(C17OO52)](7)+[(C14C15)](7)+  [(C16C17)](6)+R3[a](6)+R3[in(C17C18)](6) |
| 205 |  | 204 | R3[in(C17C18)](14)+R1[in(C24C25)](14)+R2[(δoop(C)](9)+  R2[puck](7)+R4[a](5) |
| 202 |  |  | [(O52H50)](12)+[(C17OO52)](10)+R5[’](9)+  R3[in(C17C18)](8)+R5[γ(H2+)](8)+R5[in(C55C)](8)+  R5[oop(C55C)](7)+R5[](6)+[(C16C17)](5) |
| 199 |  |  | R5[’](25)+R5[γ(H2+)](18)+R5[in(C55C)](17)+  R5[oop(C55C)](5) |
| 163 |  | 177 | R1[a](18)+R3[a](7)+R5[(C55C56)](7) |
| 159 |  |  | R5[(C55C56)](11)+R1[a](10)+[(C17OO52)](7)+  [sci(C16CC)](5)+R5[oop(C55C)](5) |
| 134 |  | 141 | R4[a](23)+R3[a](14)+[sci(C15CC)](7)+R1[in(C24C25)](7)+  [(C16C17OO52)](6) |
| 130 |  |  | [(C16C17OO52)](16)+[(C17OO52)](12)+[(C14C15)](8)+  [(O52H50)](7)+[(C17O)](6)+[(C17C18)](6)+[(C16C17)](6)+  [(C55O52O4)](5) |
| 111 |  | 108 | R2[(δin(C)](18)+R1[a](12)+R4[in(C)](10)+  [(C16C17OO52)](9) |
| 103 |  |  | [(C17OO52)](19)+R5[γ(H2+)](15)+R5[’](11)+R3[a](6)+  R4[a](6) |
| 90 |  |  | R5[γ(H2+)](33)+R5[’](21)+R5[(C55C56)](13)+  [(C17OO52)](10) |
| 81 |  |  | R2[puck](16)+[(C17OO52)](9)+[(C16C17OO52)](8)+  R2[(δoop(C)](8)+[(C14C15)](7) |
| 65 |  |  | R2[puck](15)+[(C55O52O4)](11)+R2[(δoop(C)](10)+  [(C16C17)](8)+R2[(C)](7)+[(C16C17OO52)](6)+  R2[C24C25H)](6) |
| 59 |  |  | R5[γ(H2)](28)+R5[(C55C56)](24)+[(O4O52CC)](20)+  R5[’](11)+[(C17OO52)](8) |
| 49 |  |  | [(O4O52CC)](27)+[(C16C17OO52)](17)+[(C17C18)](8)+  [sci(C17CC)](6)+[(C17O)](5) |
| 47 |  |  | [(C17C18)](57)+R3[oop(C17C18)](6)+[(C16C17OO52)](5) |
| 46 |  |  | [(O4O52CC)](58)+R2[(C)](13)+[(C17OO52C)](8) |
| 43 |  |  | R2[(C)](26)+[(O4O52CC)](18)+[sci(C17CC)](9)+  [(C55O52O4)](8)+R3[oop(C17C18)](7)+R2[puck](6)+  [(C17OO52C)](6) |
| 37 |  |  | [(C16C17OO52)](39)+[(O4O52CC)](27)+[(C17O)](14)+  [(C17OO52C)](9) |
| 32 |  |  | [(O4O52CC)](28)+R2[(C24C25)](24)+[(C55O52O4)](13)+  [(C16C17OO52)](7) |
| 30 |  |  | [(C55O52O4)](16)+R5[(C55C56)](15)+R5[γ(H2+)](14)+  [(O4O52CC)](10)+[(C16C17OO52)](10)+R5[’](6)+  [(C17OO52)](5) |
| 27 |  |  | [(C16C17OO52)](16)+R2[(C24C25)](14)+R2[(δoop(C)](12)+  R1[oop(C24C25)](11)+[(C15C16)](6) |
| 21 |  |  | [(C17OO52C)](26)+R5[γ(H2+)](18)+R5[(C55C56)](17)+R5[’](8)+  [(C15C16)](5) |
| 14 |  |  | [(C14C15)](20)+[(C16C17)](17)+R2[(δoop(C)](14)+  [(C55O52O4)](7)+[(C17OO52)](5)+[(C17C18)](5) |
| 12 |  |  | [(C16C17OO52)](21)+R2[C14C15H)](13)R2[puck](11)+  R2[(δoop(C)](5)[(C14C15)](5)+R5[γ(H2+)](5) |
| 10 |  |  | [(C16C17OO52)](25)+[(C17OO52C)](24)+[(C17OO52)](14)+  [(C17C18)](11)+[(C15C16)](5)+[(C17O)](5) |

**Table S5** Theoretical and experimental vibrational wavenumber (cm−1) of EZT+2L-proilne with potential energy distribution.

| **Scaled wavenumber** | **Experimental** | | **Potential Energy Distribution (≥5%)** |
| --- | --- | --- | --- |
|  | **IR** | **Raman** |  |
| 3465 | 3471 |  | [(O4H)](96) |
| 3416 |  |  | R6[as(H2+)](100) |
| 3411 |  |  | R5[as(H2+)](99) |
| 3202 | 3128 |  | R1[(OH)](94)+[(O69H51)](6) |
| 3115 | 3075 | 3077 | R6[(as(H2+)](78) +R5[as(H2+)](11) |
| 3105 | 3053 |  | R4[(CH)](99) |
| 3097 |  |  | R3[(CH)](99) |
| 3096 |  |  | R3[(CH)](97) |
| 3094 |  |  | R4[(CH)](97) |
| 3091 |  |  | R4[(CH)](97) |
| 3085 |  |  | R1[(CH)](98) |
| 3081 |  |  | R3[(CH)](99) |
| 3080 |  |  | R1[(CH)](98) |
| 3060 |  | 3061 | R3[(CH)](99) |
| 3060 |  |  | R1[(CH)](99) |
| 3058 | 3031 | 3036 | R5[as(CH2)](98) |
| 3048 |  |  | R1[(CH)](99) |
| 3031 |  |  | R6[as(CH2)](93) |
| 3026 |  |  | R6[as(CH2)](99) |
| 3022 |  |  | R5[as (CH2)](98) |
| 3016 |  |  | R6[(C73H)](92) |
| 3014 |  |  | R5[as (CH2)](94) |
| 3012 | 3011 | 3007 | R6[as (CH2)](85)+R5[(C73H)](13) |
| 3008 |  |  | R5[(C56H)](95) |
| 2981 |  | 2984 | [as (C15H2)](53)+[ as(C16H2)](46) |
| 2975 | 2973 | 2978 | R5[s(CH2)](99) |
| 2969 |  |  | R6[s(CH2)](99) |
| 2965 |  |  | R5[s(CH2)](90)+R2[(C14H)](5) |
| 2965 |  |  | R2[(C14H)](44)+[s(C15H2)](23)+[s(C16H2)](23) |
| 2963 |  |  | R5[s(C75H2)](96) |
| 2963 |  |  | R2[(C14H)](49)+[s(C15H2)](26)+[s(C16H2)](24) |
| 2951 |  |  | R2[(CH)](95) |
| 2950 | 2942 | 2947 | R6[s(C74H2)](98) |
| 2935 |  |  | R5[s(C57H2)](96) |
| 2932 | 2935 | 2933 | [s(C15H2)](53)+[s(C16H2)](44) |
| 2925 | 2902 | 2903 | [(C16H2)](55)+[(C15H2)](42) |
| 2879 | 2854 | 2855 | [(C17H)](97) |
| 2705 | 2741 | 2576 | R5[s(H2+)](98) |
| 2166 |  |  | R6[s(H2+)](81)+R6[γ(H2+)](6)+R6[(H2+)](5)+R6[(H2+)](5) |
| 1733 | 1736 | 1727 | R2[(C=O)](77)+R2[(C13)](9)+R2[(C13C14)](6)+R2[](5) |
| 1706 | 1605 | 1611 | R5[(COO−)](80)+R5[(C55CO)](7) |
| 1704 |  |  | R6[(COO−)](80)+R6[(C72CO)](9) |
| 1602 |  |  | R1[(CC)](60)+R1[in(CH](15)+R1[a](10) |
| 1595 | 1578 | 1588 | R4[(CC)](59)+R4[in(C8H)](19)+R4[a](11) |
| 1592 |  |  | R3[(CC)](46)+R5[(H2+)](17)+R5[(H2+)](11)+R3[a](8)+  R3[in(CH)](8) |
| 1590 |  |  | R5[(H2+)](54)+R5[(H2+)](35) |
| 1584 |  |  | R3[(CC)](71)+ R3[’a](9) |
| 1579 |  |  | R4[(C9C10)](75)+R4[a’](9) |
| 1576 |  |  | R1[(CC)](59)+R1[in(OH](12)+R1[in(C26H)](7)+R1[’a](7) |
| 1561 |  |  | R6[(H2+)](71)+R6[(H2+)](17) |
| 1499 | 1509 | 1511 | R1[in(CH](45)+R1[(CC)](37)+R1[(CO)](9) |
| 1489 |  |  | R4[in(CH)](45)+R4[(CC)](32)+R4[(C7)](9)+R4[(CF)](5) |
| 1487 |  |  | R3[in(CH)](48)+R3[(CC)](34)+R3[(CF)](6) |
| 1470 |  | 1475 | R6[(CH2)](95) |
| 1463 |  |  | R5[(CH2)](93) |
| 1455 |  |  | R6[(CH2)](97) |
| 1451 |  |  | R5[(CH2)](88)+[sci(C15H2)](6) |
| 1449 | 1449 | 1451 | R6[(CH2)](80)+R1[in(OH](6) |
| 1448 |  |  | R1[in(OH](28)+R1[(CC)](17)+R6[(CH2)](16)+  R1[in(C27H)](11) |
| 1448 |  |  | R5[(C57H)](96) |
| 1444 |  | 1441 | [sci(C15H2)](62)+[sci(C16H2)](24)+R5[(C59H2)](5) |
| 1430 | 1437 |  | [sci(C16H2)](68)+[sci(C15H2)](24) |
| 1408 |  | 1426 | R4[(CC)](42)+R4[in(CH)](33)+R4[in(C)](9) |
| 1401 | 1386 | 1389 | R3[(CC)](27)+R3[in(CH)](25)+[sci(C17OH)](8)+  [(C17OH)](7)+[γ(C17OH)](7)+[(C17OH)](5)+  R3[in(C17C18)](5) |
| 1375 |  |  | R1[in(OH](27)+R1[in(C2H)](27)+R1[(CC)](25)+  R2[(C24C25H)](6) |
| 1368 |  |  | [in(O4H)](28)+[sci(C17OH)](19)+[(C16H2)](11)+  [(C17OH)](8)+[(C16C17)](8)+[(C17OH)](5) |
| 1361 |  |  | R6[(CH2)](45)+R6[(H2+)](23)+R6[γ(H2+)](6)+  R5[(C75C76)](6) |
| 1359 | 1352 | 1353 | R4[(C7)](20)+R1[(CC)](8)+R2[(C)](17)+  R2[(C14C15H)](6)+[(C15H2)](5) |
| 1357 |  |  | R5[(C59H2)](31)+[(C15H2)](14)+[(C16H2)](11)+  R5[(H2+)](10) |
| 1354 |  |  | R5[(C59H2)](26)+[(C15H2)](13)+[sci(C17OH)](10)+  [(C16H2)](7)+[in(O4H)](5) |
| 1339 |  |  | R2[(C24C25H)](26)+R2[(C24C25H)](10)+R4[(C7)](8)+  R2[(C13)](6)+R1[(CC)](9) R1[in(CH)](9) |
| 1334 |  |  | [(C17OH)](19)+[(C15H2)](11)+[γ(C17OH)](9)+R3[(CC)](12)+ [sci(C17OH)](6)+R3[in(C20H)](7) |
| 1325 | 1326 | 1334 | R6[(COO−)](33)+R6[(CH2)](18)+R5[(C72C)](10)+  R6[γ(CH2)](10)+R6[sci(C72CO)](9) |
| 1317 |  |  | R6[(CH2)](42)+R6[in(C73H)](19)+R5[(C74C75)](8)+  R6[γ(H2+)](5) |
| 1314 | 1305 | 1307 | R5[(COO−)](11)+R5[(CH2)](15)+R5[γ(CH2)](16)+  R5[(H2+)](7) |
| 1314 |  |  | R5[(CH2)](42)+R5[in(C56H)](15)+R5[(C57C58)](7)+  R5[γ(C59H2)](9)+R5[(H+)](6) |
| 1306 |  |  | R6[γ(H2+)](25)+R6[γ(CH2)](12)+R6[(COO−)](11)+  R6[oop(C73H)](10)+R6[in(C73H)](7)+R6[(H2+)](6)+  R6[sci(C72CO)](5) |
| 1304 |  | 1298 | R2[(C14C15H)](11)+[(C16H2)](9)+[in(O4H)](6) |
| 1298 |  |  | [γ(C16H2)](19)+[γ(C15H2)](15)+[(C17OH)](7)+[γ(C17OH)](6) |
| 1294 |  | 1290 | R4[(CC)](16)+[γ(C15H2)](12)+R5[oop(C56H)](7)+  [γ(C16H2)](6)+R5[γ(H2+)](5)+R5[(COO−)](5) |
| 1289 |  |  | R6[(CH2)](75)+R6[in(C73H)](8) |
| 1289 | 1287 |  | R4[(CC)](68) |
| 1285 |  |  | R5[(CH2)](60)+R5[in(C56H)](20)+R5[(H2+)](6) |
| 1281 |  |  | R4[in(CH)](57)+R4[(CC)](33)+R4[in(CF)](5) |
| 1280 |  |  | R3[(CC)](46)+R3[in(CH)](21) |
| 1275 | 1270 | 1271 | R1[in(CH](19)+R3[in(CH)](17)+R1[(CC)](14)+  R2[(C24C25H)](6) |
| 1267 |  |  | R3[in(CH)](16)+R3[(CC)](11)+R1[in(CH](10)+  R1[(CO)](5)+ [(C15H2)](5) |
| 1265 |  |  | [in(O4H)](14)+[γ(C15H2)](13)+[sci(C17OH)](7)+  R1[(CO)](7)+ |
| 1259 |  |  | R6[oop(C73H)](20)+R1[(CO)](15) R6[γ(CH2)](14) |
| 1256 |  |  | R1[(CC)](18)+R6[oop(C73H)](14)+R1[(CO)](11)+  R6[γ(C75H2)](5) |
| 1252 |  | 1258 | R5[(COO−)](16)+R5[oop(C56H)](10)+R5[(C55C)](9)+  R5[(H2+)](7)+R5[γ(C58H2)](7) |
| 1242 |  |  | R1[(CC)](23)+R5[γ(C59H2)](9)+R1[in(C26H)](9)+  R1[(CC)](8)+R1[in(OH](7)+R1[(CO)](6)+R6[γ(C76H2)](5) |
| 1241 |  |  | R5[γ(C59H2)](39)+R5[oop(C56H)](9) |
| 1235 |  |  | R6[γ(C76H2)](41)+R6[in(C73H)](7)+R6[γ(H2+)](6)+  R6[(C75H2)](5) |
| 1233 |  |  | [(C16H2)](19)+[(C17OH)](13)+R2[(C24C25H)](7)+  R3[(CC)](7) [(C15H2)](6)+[γ(C17OH)](6) |
| 1222 | 1223 | 1223 | R6[in(C73H)](29)+R6[(C76H2)](16)+R6[γ(C76H2)](12)+  R6[(H2+)](9)+R6[γ(H2+)](7)+R5[(C73C74)](6) |
| 1221 |  |  | R5[in(C56H)](27)+R5[γ(H2+)](15)+R5[(H2+)](13)+  R5[(CH2)](12)+R5[(CH2)](9)+R5[(C56C57)](5) |
| 1214 |  |  | R2[(CCH)](17)+R2[(CC)](10)+R2[(C14C15H)](8)+  R2[(γ(C24C25H)](5)+[in(O4H)](5) |
| 1197 | 1201 | 1205 | R4[(CF)](41)+R4[in(CH)](16)+R4[(C8C9)](14)+R4[tri](11) |
| 1196 |  | 1195 | R3[(CF)](13)+R5[(H2+)](11)+R5[γ(C58H2)](9)+  R5[(COO−)](7)+R5[(H2+)](5) |
| 1196 |  |  | R3[(CF)](32)+R3[(CC)](15)+R3[in(CH)](7)+R5[(H2+)](6)+R5[γ(C58H2)](5)+R3[tri](5) |
| 1190 |  |  | R2[(C24C25)](22)+R2[(C24C25H)](12)+R1[(CC)](18)+  R1[in(CH)](9)+R1[tri](7)+R2[(C24C25H)](5) |
| 1180 |  |  | R2[(C14C15H)](12)+[(C17OH)](9)+[γ(C16H2)](9)+  R2[(C14C15H)](8)+[γ(C17OH)](6)+[(C15H2)](6)+  [in(O4H)](5)+[(C15H2)](5) |
| 1173 | 1165 | 1166 | [(C17C18)](34)+R3[tri](15)+R3[in(C19H)](9)+R3[(CC)](12) |
| 1166 |  |  | R6[γ(C75H2)](32)+R6[(CH2)](22)+R5[(CC)](16)+R6[](7) |
| 1155 |  |  | R5[γ(CH2)](26)+R5[(CH2)](17)+R1[in(CH](12)+R5[(CC)](12)+R5[(CH2)](8)+R5[](6) |
| 1155 | 1154 | 1155 | R1[in(CH](51)+R5[γ(CH2)](10)+R5[(C58H2)](6) |
| 1145 | 1145 |  | R6[γ(CH2)](38)+R6[oop(C73H)](19)+R6[(C76H2)](12)+  R6[(H2+)](10) |
| 1139 |  |  | R4[in(CH)](58)+R4[(CF)](7) |
| 1138 |  |  | R2[(C14C15H)](20)+R2[(γ(C14C15H)](14)+[γ(C15H2)](10) [γ(C16H2)](8) |
| 1136 |  |  | R3[in(CH)](63)+R3[(CF)](7) |
| 1124 |  |  | R2[(C)](26)+R2[(C24C25H)](25)+R2[(γ(C24C25H)](17)+  R2[(δin(C=O)](6)+R4[in(C8H)](5)+R2[(C13C14)](5) |
| 1122 | 1126 | 1124 | R5[oop(C56H)](31)+R5[(H2+)](19)+R5[γ(C58H2)](18)+  R5[γ(H2+)](8)+R5[(C59H2)](5) |
| 1101 |  |  | R1[in(CH)](30)+R1[(CC)](12)+R2[(C14C15)](5)+  R2[(C24)](5) |
| 1092 | 1095 | 1096 | R1[in(CH)](22)+R4[in(CH)]22)+R1[(C26C27)](11)+  R4[(C11C12)](9)+R2[(C14C15)](6) |
| 1084 |  |  | R3[in(CH)](33)+R3[(CC)](15)+R2[(C14C15)](7) |
| 1080 |  | 1079 | R3[in(CH)](16)+R2[(C14C15)](11)+R4[in(CH)](10)+  R4[(C8C9)](6)+[(C15C16)](6) R3[(CC)](8) |
| 1079 | 1067 |  | R6[(CH2)](23)+R6[(H2+)](9)+R6[γ(H2+)](6)+R6[(H2+)](6)+R6[()](6)+R6[(C)](11)+R6[γ(C75H2)](7)+  R6[oop(C73H)](6) |
| 1062 | 1057 | 1056 | [(CO4)](13)+R5[(C57H2)](10)+[(C15H2)](6)+[(C16H2)](6)+  R2[(γ(C14C15H)](5)+R5[γ(C57H2)](5) |
| 1061 |  |  | R5[(CH2)](24)+R5[γ(C57H2)](10)+R5[(CC)](8)+  R5[in(C55C)](7) |
| 1048 |  |  | R6[γ(H2+)](14)+R6[(H2+)](14)+R6[(C76)](11)+  R6[in(CC)](11)+R5[(CC)](9)+R6[γ(C74H2)](8)+R5[(CH2)](7) |
| 1043 |  |  | R2[(C24)](26)+[(CO4)](18)+R4[(C7C12)](6) |
| 1021 | 1030 |  | [(C16C17)](31)+[(C16H2)](12)+[in(O4H)](7)+[(CO4)](7)+  [(C15H2)](5) |
| 1016 |  |  | R5[(CC)](31)+R6[(C)](11)+R6[in(C73H)](8)+R6[γ(H2+)](8)+R6[(C75H2)](8)+R6[(C74H2)](5) |
| 1014 | 1014 | 1040 | R5[(CC)](41)+R5[(C57H2)](12)+R5[](9)+R5[in(C56H)](8)+R5[(CH2)](7)+R5[(H2+)](5) |
| 1004 |  | 1030 | [(C15C16)](34)+R2[(C14C24)](11)+R2[(C14C15H)](8)+  [(CO4)](8)+[sci(C15CC)](5)+[(C16C17)](5) |
| 998 | 988 | 986 | R3[tri](46)+R3[(CC)](34)+R3[in(CH)](10) |
| 995 |  |  | R4[tri](47)+R4[(C)](36)+R4[in(CH)](13) |
| 993 |  |  | R1[tri](47)+R1[(CC)](36)+R1[in(CH)](9) |
| 992 |  |  | R5[(C)](28)+R5[(CC)](24)+R5[’](16)+R5[(C58H2)](9)+  R5[(C56C57)](8) |
| 976 |  |  | R5[(C75C76)](20)+R6[(H2+)](17)+R6[(H2+)](16)+R6[’](6)+R6[γ(C75H2)](6)+R6[oop(C72C)](5) |
| 950 |  |  | [(CO4)](15)+R1[oop(CH)](12)+[(C15H2)](9)+  R2[(C14C24)](9) [(C16C17)](8)+[γ(C16H2)](6) |
| 945 |  |  | R3[oop(CH)](55)+R1[oop(CH)](28)+R3[’a](5) |
| 945 | 957 | 958 | R6[(H2+)](26)+R6[γ(C76H2)](10)+R6[(H2+)](9)+  R6[(C74H2)](9)+R6[oop(C72C)](8) +R5[(C75C76)](5) |
| 945 |  |  | R5[(H2+)](12)+R5[oop(C55C)](11)+R5[(C57H2)](10)+  R5[γ(C59HH)](9)+R5[γ(H2+)](7)+R5[(H2+)](5) |
| 944 |  |  | R1[oop(CH)](49)+R3[oop(CH)](25) |
| 943 |  |  | R4[oop(CH)](87)+R4[a’](7) |
| 926 |  | 927 | R1[oop(CH)](39)+R3[oop(CH)](28)+R1[puck](9) |
| 923 |  |  | R3[oop(CH)](45)+R1[oop(CH)](23)+R3[puck](11) |
| 918 | 923 |  | R5[(CC)](27)+R6[(C76)](15)+R56[(C76H2)](12)+  R6[’](6)+R6[(H2+)](6) |
| 917 | 910 | 911 | R4[oop(CH)](77)+R4[puck](18) |
| 910 |  |  | R5[(CC)](40)+R5[(C59)](14)+R5[(C59H2)](15)+R5[’](8) |
| 891 | 893 | 895 | R2[(CC)](21)+R2[](8)+[(C15H2)](5)+R1[oop(CH)](6)+  [(CO4)](5) |
| 884 |  |  | R5[(CC)](73)+R5[(C)](7) |
| 883 | 871 |  | R2[(CC)](24)+[(C15C16)](6)+[(C16H2)](5)+  R2[(C14C15H)](5) |
| 881 |  |  | R5[(CC)](69)+R6[(C76)](8) |
| 872 |  |  | R6[(C)](33)+R5[(CC)](18)+R6[(C74H2)](6) |
| 863 | 861 | 862 | R5[(C)](36)+R5[(CH2)](16)+R5[γ(H2)](8)+R5[(C55C)](7) |
| 849 |  |  | R1[(CC)](31)+R1[a](6) |
| 840 | 850 | 844 | R6[(H2+)](22)+R56[(CH2)](16)+R6[(C73)](14)+  R5[(C75C76)](7)+R6[oop(C72C)](6)+R6[(C76)](6) |
| 831 | 833 | 833 | R3[(CC)](21)+R1[oop(CH)](12)+R3[a](10)+[(C17C18)](7)+  R3[(CF)](6) |
| 829 |  |  | R5[(CH2)](27)+R5[(C59)](21)+R5[γ(H2+)](10)+  R5[(H2+)](9)+R5[(CC)](9) |
| 829 |  |  | R1[oop(CH)](40)+R1[oop(CO)](12)+R1[a](8) |
| 826 | 828 |  | R4[oop(CH)](50)+R4[a](10)+R4[oop(CF)](8)+  R4[oop(C)](7) |
| 825 |  | 825 | R6[(C)](26)+R6[(CH2)](22)+R6[sci(C72CO)](5) |
| 824 |  |  | R5[(C56)](37)+R5[(CH2)](21)+R5[(H2+)](10)+  R5[γ(H2+)](8)+R5[(C55CO)](6) |
| 823 | 816 |  | R3[oop(CH)](48)+R3[a](9) |
| 821 |  |  | R1[(CO)](52)+R1[oop(CH)](25)+[(C73C72H51O5)](21) |
| 809 |  | 809 | R6[sci(C72CO)](22)+R5[(CC)](14)+R56[(CH2)](12) |
| 806 |  |  | R4[a](12)+R4[(CF)](10)+R6[sci(C72CO)](8)+  R4[(C10C11)](5)+R4[(C9C10)](5) |
| 802 |  |  | R1[tri](9)+R1[(CO)](8)+R1[(CC)](8) |
| 802 |  | 800 | R3[oop(CH)](95) |
| 798 |  |  | R5[sci(C55CO)](31)+R5[(C55C)](20)+R5[oop(C55C)](7)+  R5[(C55CO)](6)+[(O4O52CC)](5) |
| 794 |  |  | R4[oop(C8H)](33)+R4[oop(C9H)](33)+R4[oop(C11H)](18)+  R4[oop(C12H)](10) |
| 788 | 785 | 786 | R1[(CO)](50)+[(C73C72H51O5)](24)+R1[oop(C27H)](10)+  R1[oop(C26H)](6) |
| 775 |  |  | R3[(CF)](9) R3[tri](9)+R2[](7) |
| 765 | 763 | 763 | R6[(C72CO)](24)+R6[oop(C72C)](17)+R6[sci(C72CO)](10)+R6[(C73)](9)+R6[oop(C73H)](9)+R6[in(C72C)](5)+  R5[(C73C74)](5) |
| 761 |  |  | [(C16H2)](16)+R5[(C55CO)](8)+[(C15H2)](7)+  R5[sci(C55CO)](5) |
| 758 |  |  | R5[(C55CO)](16)+R5[sci(C55CO)](10)+R5[oop(C55C)](8)+  [(C16H2)](7)+R5[oop(C56H)](6)+R5[(C56)](5)+  R5[γ(H2+)](5) |
| 752 | 732 |  | R1[puck](9)+R2[(C14C15)](8)+R3[tri](6)+R2[](6)+  R1[oop(CO)](5)+R3[(CF)](5) |
| 713 | 712 | 717 | R3[puck](53)+R1[puck](11)+R3[oop(CF)](11)+  R3[oop(C17C18)](10) |
| 708 |  | 701 | R1[puck](47)+R1[oop(CO)](14)+R3[puck](11)+  R1[oop(C24C25)](7)+ |
| 696 |  |  | R4[puck](66)+R4[oop(C)](15)+R4[oop(CF)](14) |
| 689 |  |  | [(C15H2)](19)+R2[(δin(C=O)](12)+[(C15C16)](10)+  [(C17O)](8)+[(C16HH)](6)+R3[puck](6) |
| 674 | 668 | 666 | [(C17O)](76) |
| 649 | 645 | 635 | R6[](46)+R6[(C72CO)](12)+R56[(CH2)](9)+  R6[(C72CO)](7)+R6[(H2+)](5) |
| 643 |  |  | R5[](43)+R5[(H2+)](13)+R5[(C55CO)](6)+  R5[γ(H2+)](5)+R5[sci(C55CO)](5) |
| 630 |  |  | R1[’a](73)+R1[in(CO](5) |
| 628 |  |  | R3[’a](71)+R3[in(CF)](5) |
| 627 |  |  | R4[a’](64)+R3[’a](5)+R4[in(CF)](5) |
| 616 | 615 | 617 | R5[(C72C)](22)+R6[(C72CO)](11)+R5[(C73C74)](11)+  R6[(C72CO)](10)+R6[oop(C72C)](9)+R6[sci(C72CO)](8)+  R6[in(C72C)](8) |
| 604 | 593 | 593 | R2[(CC)](12)+R4[a](9)+R4[a’](7)+R2[(δin(C=O)](6)+  R4[(C7)](5) |
| 590 |  |  | R5[(C55C)](17)+R5[(C55CO)](13)+R5[sci(C55CO)](10)+  R5[γ(H2+)](10)+R5[(C55CO)](8)+R5[oop(C55C)](8)+  [(O4O52CC)](7)+R5[(C56C57)](7) |
| 580 | 570 | 572 | R2[](14)+R4[a](12)+R1[oop(CO)](6)+R2[(γ(C24C25H)](6) R1[a](6) |
| 563 |  |  | [(C17OH)](21)+R3[a](12)+R5[’](6)+R3[’a](5)+[sci(C15CC)](5)+R3[i(C17C18)](5) |
| 561 | 561 | 560 | R5[’](43)+R5[(CH2)](29)+R5[(C55CO)](6) |
| 561 |  |  | R6[’](48)+R6[(CH2)](33)+R6[(C72CO)](5) |
| 551 |  |  | R1[a](12)+R2[(δin(C=O)](12)+R3[a](6+R3[oop(CF)](5)+  R1[oop(CO)](5) |
| 538 | 540 | 540 | R3[oop(CF)](20)+R3[a](18)+R3[oop(C17C18)](10)+R1[a](6)+R1[oop(CO)](5)+[sci(C17CC)](5) |
| 516 |  |  | R1[oop(CO)](15)+R1[a](10)+R4[a](10)+R4[oop(CF)](10)+  R4[oop(C)](9)+R3[a](6)+R3[oop(CF)](6)+  R1[oop(C24C25)](6) |
| 511 | 508 | 512 | R1[oop(CO)](20)+R1[a](14)+R1[oop(C24C25)](6)+  R2[(δin(C=O)](5) |
| 504 |  |  | R4[a](23)+R4[oop(CF)](22)+R4[oop(C)](17)+  R1[oop(CO)](5) |
| 468 | 469 | 469 | R6[(C72CO)](20)+R1[in(CO](13)+R5[(C72C)](11)+  R6[in(C72C)](8)+R6[](7) |
| 456 | 456 | 457 | R5[(C55CO)](28)+R5[(C55C)](12)+R5[in(C55C)](12)+  R5[](8)+R5[(C56)](6)+R5[(C55CO)](5) |
| 448 |  | 446 | R1[in(CO](11)+[sci(C15CC)](7)+[(C15C16)](5)+  R2[(C14C15H)](5) |
| 438 | 429 | 429 | R1[in(CO](22)+R1[’a](5)+sci(C15CC)](5) |
| 417 |  |  | R4[in(CF)](30)+R4[a’](11)+R4[in(C)](10)+R3[oop(CF)](6) |
| 414 | 421 |  | R1[’a](25)+R3[’a](20)+R4[in(CF)](5) |
| 412 |  |  | R4[a’](81 |
| 412 |  |  | R3[’a](43)+R1[’a](34) |
| 409 | 408 | 408 | R1[’a](26)+R3[’a](21) R3[in(CF)](7) |
| 398 |  | 396 | R3[in(CF)](24)+R1[’a](10)+R1[puck](9)+R1[oop(CO)](6)+  R4[a](6)+R3[’a](5) |
| 392 |  | 382 | R3[in(CF)](22+R3[puck](8)+R3[oop(CF)](7)+R1[puck](5) |
| 370 |  |  | R4[puck](20)+R4[oop(CF)](19)+R4[oop(C)](12)+  R2[(δoop(C)](7) |
| 343 |  | 359 | R4[in(CF)](16)+R2[(δin(C)](8)+R4[in(C)](8)+R1[a](6)+  R1[in(C24C25)](6) |
| 337 |  | 332 | R5[γ(H2+)](26)+R5[’](17)+R5[oop(C55C)](16)+  R5[(H2+)](9)+R5[(C55CO)](9) |
| 311 |  |  | [(C17OO52)](14)+R3[a](10)+[γ(C17OH)](9)+[(C17C18)](7)+  [(C17OH)](6)+R3[oop(C17C18)](5) |
| 298 |  | 294 | R6[oop(C72C)](29)+R6[](20)+R6[in(C72C)](19)+  R6[(H2+)](6)+R6[(C72CO)](5) |
| 290 |  |  | R5[](20)+R5[in(C55C)](17)+R5[(C55CO)](9)+R5[’](7) |
| 288 |  | 275 | R5[](10)+R5[in(C55C)](8)+R4[a](7)+R1[a](7)+  R1[oop(C24C25)](6)+R1[puck](5)+R5[(C55CO)](5) |
| 268 |  | 259 | R4[in(C)](14)+R2[(δin(C)](8)+R2[(δin(C=O)](7)+R1[a](5)+R4[in(CF)](5) |
| 256 |  |  | R6[(H2+)](32)+R6[(C72CO)](15)+R6[’](14)+R6[(H2+)](9)+R6[γ(H2+)](8)+R6[oop(C72C)](5) |
| 253 |  |  | sci(C16CC)](17)+[(C14C15)](7)+[(C15C16)](6)+  [(C16C17)](5) |
| 235 |  |  | R2[(δoop(C)](10)+R3[a](9)+R4[a](8)+[(C15C16)](7)+  [sci(C17CC)](5)+R3[oop(C17C18)](5) |
| 222 |  |  | R1[in(C24C25)](11)+[(C15C16)](8)+R2[(δoop(C)](7)+  R2[puck](7)+[(O69H51)](6)+[(C14C15)](6)+[(O52H50)](5) |
| 211 |  | 204 | [(O52H50)](13)+R3[in(C17C18)](9)+R2[(γ(C14C15H)](5)+  [(C16C17)](5) |
| 198 |  |  | R3[in(C17C18)](13)+R5[’](12)+[(C16C17)](10)+  [(O52H50)](8)+R5[γ(H2+)](7)+(C17OO52C)](5)+  [(C15C16)](5)+[(C17OH)](5) |
| 190 |  |  | R5[’](19)+R5[γ(HH2)](12)+R5[in(C55C)](11)+R6[(H2)](10)+R5[](7)+R5[oop(C55C)](5) |
| 188 |  |  | R6[(H2+)](26)+R6[(C72CO)](12)+R6[in(C72C)](9) |
| 173 |  | 177 | [(O52H50)](11)+R1[a](10)+[(C15C16)](9)+R5[oop(C55C)](7)+R1[(CO)](7)+[(O5O69CC)](6) R5[](5) |
| 154 |  | 141 | R1[a](11)+R1[(CO)](10)+[(O5O69CC)](10)+  [(C16C17OO52)](8) |
| 150 |  |  | R6[oop(C72C)](24)+R6[in(C72C)](15)+R6[](10)+  R6[(H2)](9) R6[(C72C)](8)+[(O69H51)](8) |
| 143 |  |  | R6[(C72C)](13)+[(O69H51)](13)+R6[(H2)](11)+  R6[(H2)](9)+R6[(C72CO)](8)+R6[(H2)](5)+  R6[oop(C72C)](5) |
| 136 |  |  | [(O69H51)](12)+[(O5O69CC)](11)+R1[(CO)](10)+R3[a](6)+R2[puck](5) |
| 118 |  |  | [(C17OO52)](16)+[(C16C17OO52)](11)+[(C14C15)](8)+  [(C16C17)](7)+R4[a](6)+[(C17O)](5) |
| 110 |  | 108 | [(C15C16)](14)+[(O5O69CC)](11)+R1[(CO)](10)+  R2[(δin(C)](10)+[(C17OO52)](9)+R5[’](8)+R5[γ(H2)](7)+  R4[in(C)](5) |
| 100 |  |  | R5[γ(H2)](28)+R5[’](25)+[(O52H50)](9)+R5[oop(C55C)](5) |
| 92 |  |  | R1[(CO)](24)+[(O5O69CC)](23)+R6[(C72C)](9)+R6[’](8)+  [(C17OO52)](7)+R2[puck](5) |
| 87 |  |  | [(C17OO52)](18)+R6[(C72C)](14)+R6[’](12)+R1[(CO)](11)+[(O4O52CC)](10)+[(O5O69CC)](9) |
| 84 |  |  | [(O4O52CC)](19)+[(C17OO52)](15)+R5[γ(H2)](9)+[(C16C17OO52)](8)+R5[(C55C56)](8)+R5[’](6)+R6[(C72C)](5) |
| 74 |  |  | R5[γ(H2)](26)+R5[(C55C56)](20)+R5[’](14)+  [(C55O52O4)](10)+ [(C17OO52)](6)+R5[(H2)](5) |
| 61 |  |  | [(O5O69CC)](41)+R1[(CO)](36)+R6[(C72C)](5) |
| 57 |  |  | [(O5O69CC)](31)+R1[(CO)](29)+[(O4O52CC)](7)+  [(C16C17)](5) |
| 48 |  |  | [(O4O52CC)](23)+[(O5O69CC)](16)R1[(CO)](16)+  [(C16C17OO52)](5)+ [(C28OO69)](5) |
| 43 |  |  | [(O4O52CC)](37)+R5[γ(H2)](11)+R2[(C)](8)+R5[’](8)+  R5[(C55C56)](7)+[(C17OO52)](5) |
| 42 |  |  | [(O4O52CC)](16)+R2[(C)](13)+R5[γ(H2)](13)+R5[’](9)+  [(C17OO52)](8)+R1[(CO)](8)+[(O5O69CC)](7)+  R5[(C55C56)](7) |
| 36 |  |  | [(C73C72H51O5)](36)+R2[(C24C25)](22)+[(O4O52CC)](5)+  R1[oop(C24C25)](5) |
| 35 |  |  | [(C16C17OO52)](20)+[(C55O52O4)](8)+[(C17O)](7)+  [(C28OO69)](7)+[(C17OO52C)](5) |
| 32 |  |  | [(O4O52CC)](38)+[(C55O52O4)](33)+[(C17OO52C)](6)+  [(C16C17OO52)](6) |
| 28 |  |  | [(C28OO69C)](18)+[(C73C72H51O5)](13)+[(O5O69CC)](10)+R1[oop(C24C25)](6)+[(C16C17OO52)](6)+R2[(δoop(C)](6) |
| 24 |  |  | [(C72O69O5)](20)+[(C55O52O4)](13)+[(C15C16)](12)+  [(C73C72H51O5)](7)+[(C28OO69)](6)+[(C17C18)](6)+  R2[(δoop(C)](5)+[(C17OO52)](5) |
| 21 |  |  | [(C17OO52C)](27)+[(C16C17OO52)](15)+[(O4O52CC)](14)+  [(C15C16)](7) [(C17OO52)](7)+[(C17C18)](7)+R5[γ(H2)](6) |
| 20 |  |  | [(C17OO52C)](39)+[(C17O)](19)+[(C16C17OO52)](8)+  R5[γ(H2)](8)+R5[’](7) |
| 17 |  |  | [(C16C17OO52)](16)+[(C72O69O5)](11)+[(C55O52O4)](10)+[(C17OO52C)](9)+[(C17C18)](8)+[(C17O)](8)+  [(C28OO69)](8) |
| 15 |  |  | [(O5O69CC)](28)+R1[(CO)](21)+[(C16C17OO52)](20)+  [(C17O)](9) |
| 13 |  |  | [(C16C17OO52)](40)+[(C17O)](19)+[(C17OO52C)](11) |
| 12 |  |  | [(C16C17OO52)](38) [(C17O)](15)+[(C17OO52C)](10)+R1[(CO)](8) [(O5O69CC)](7) |
| 8 |  |  | [(C28OO69C)](24)+[(C16C17OO52)](16)+[(O5O69CC)](10)+  [(C17O)](10) [(C73C72H51O5)](7) |
| 7 |  |  | [(C17OO52C)](25)+[(C14C15)](18)+[(C28OO69)](7)+  [(C72O69O5)](6)+[(C16C17)](6)+R1[(CO)](5)+[(C16C17OO52)](5)+R2[puck](5) |

**Table S6** Second order perturbation theory analysis of Fock matrix in NBO for cocrystal using B3LYP/6-311++G(d,p).

| **Donor Orbitals** | **Acceptor Orbitals** | **Donor occupancy** | **Acceptor occupancy** | **E(i,j)** |
| --- | --- | --- | --- | --- |
| **Within unit 1** | | | | |
| C7-C8) | C9-C10) | 1.64921 | 0.37935 | 20.48 |
| C7-C8) | C11-C12) | 1.64921 | 0.33857 | 19.69 |
| C9-C10) | C7-C8) | 1.66581 | 0.39404 | 19.35 |
| C9-C10) | C11-C12) | 1.66581 | 0.33857 | 19.87 |
| C11-C12) | C7-C8) | 1.69591 | 0.39404 | 19.63 |
| C11-C12) | C9-C10) | 1.69591 | 0.37935 | 21.29 |
| C18-C19) | C20-C21) | 1.66080 | 0.37012 | 19.96 |
| C18-C19) | C22-C23) | 1.66080 | 0.33107 | 21.39 |
| C20-C21) | C18-C19) | 1.66745 | 0.34580 | 19.83 |
| C20-C21) | C22-C23) | 1.66745 | 0.33107 | 18.51 |
| C22-C23) | C18-C19) | 1.68214 | 0.34580 | 19.21 |
| C22-C23) | C20-C21) | 1.68214 | 0.37012 | 23.01 |
| C25-C26) | C27-C28) | 1.67929 | 0.38275 | 17.11 |
| C25-C26) | C29-C30) | 1.67929 | 0.32977 | 21.93 |
| C27-C28) | C25-C26) | 1.61916 | 0.39351 | 24.57 |
| C27-C28) | C29-C30) | 1.61916 | 0.32977 | 17.08 |
| C29-C30) | C25-C26) | 1.70481 | 0.39351 | 17.86 |
| C29-C30) | C27-C28) | 1.70481 | 0.38275 | 21.77 |
| LP(3)F1 | C9-C10) | 1.93125 | 0.37935 | 17.28 |
| LP(3)F2 | C20-C21) | 1.92972 | 0.37012 | 17.64 |
| LP(2)O3 | N6-C13) | 1.84029 | 0.08591 | 27.57 |
| LP(2)O3 | C13-C14) | 1.84029 | 0.07703 | 20.62 |
| LP(2)O5 | C27-C28) | 1.85369 | 0.38275 | 30.89 |
| LP(1)N6 | O3-C13) | 1.62233 | 0.29522 | 60.01 |
| LP(1)N6 | C7-C8) | 1.62233 | 0.39404 | 35.81 |
| **from unit 1 to unit 2** | | | | |
| O4-H50) | N54-H61) | 1.98693 | 0.09092 | 0.23 |
| LP(2)O3 | C59-H67) | 1.84029 | 0.01248 | 0.36 |
| LP(1)O4 | N54-H61) | 1.97604 | 0.09092 | 0.39 |
| LP(1)O4 | C59-H67) | 1.97604 | 0.01248 | 0.20 |
| **from unit 1 to unit 3** | | | | |
| O5-H51) | O69-C72) |  | 0.06053 | 0.17 |
| LP(1)O5 | O69-C72) |  | 0.06053 | 0.16 |
| LP(2)O5 | C74-H80) |  | 0.01089 | 0.17 |
| **from unit 2 to unit 1** | | | | |
| LP(1)O52 | O4-H50) | 1.96102 | 0.02682 | 5.21 |
| LP(3)O52 | O4-H50) | 1.66687 | 0.02682 | 4.62 |
| **within unit 2** | | | | |
| LP(2)O52 | O53-C55) | 1.83692 | 0.05475 | 16.64 |
| LP(2)O52 | N54-H61) | 1.83692 | 0.09092 | 25.74 |
| LP(2)O52 | C55-C56) | 1.83692 | 0.12058 | 12.66 |
| LP(2)O52 | O53-C55) | 1.83692 | 0.30243 | 68.49 |
| LP(2)O53 | O52-C55) | 1.66687 | 0.06544 | 22.57 |
| LP(2)O53 | C55-C56) | 1.66687 | 0.12058 | 22.30 |
| **from unit 3 to unit 1** | | | | |
| LP(1)O69 | O5-H51) | 1.95981 | 0.05814 | 7.87 |
| LP(2)O69 | O5-H51) | 1.85095 | 0.05814 | 18.65 |
| **within unit 3** | | | | |
| LP(2)O69 | O70-C72) | 1.85095 | 0.05508 | 22.96 |
| LP(2)O69 | C72-C73) | 1.85095 | 0.11274 | 14.77 |
| LP(2)O70 | O69-C72) | 1.77726 | 0.06053 | 16.63 |
| LP(2)O70 | C72-C73) | 1.77726 | 0.11274 | 10.86 |
| LP(3)O70 | O69-C72) | 1.63459 | 0.06053 | 75.32 |
| O69-C72) | O69-C72) | 0.06053 | 0.06053 | 10.87 |

**Table S7**Reactivity descriptors as Fukui functions (*fk0, fk+, fk−*), local softness (*Sk0, Sk+, Sk−*), local electrophilicity indices (*ωk0, ωk+, ωk−*) for EZT-L-proline using Hirshfeld atomic charges.

| **atoms** | ***fk0*** | ***fk+*** | ***fk−*** | ***Sk0*** | ***Sk+*** | ***Sk−*** | ***ωk0*** | ***ωk+*** | ***ωk−*** |
| --- | --- | --- | --- | --- | --- | --- | --- | --- | --- |
| 1 F | −0.1136 | 0.0432 | 0.0109 | −0.0234 | 0.0089 | 0.0022 | −0.3261 | 0.1240 | 0.0313 |
| 2 F | −0.1133 | 0.0104 | 0.0238 | −0.0234 | 0.0021 | 0.0049 | −0.3250 | 0.0297 | 0.0684 |
| 3 O | −0.2928 | 0.0607 | 0.0091 | −0.0604 | 0.0125 | 0.0019 | −0.8402 | 0.1741 | 0.0260 |
| 4 O | −0.2371 | 0.0048 | 0.0068 | −0.0489 | 0.0010 | 0.0014 | −0.6802 | 0.0138 | 0.0195 |
| 5 O | −0.1991 | 0.0393 | 0.0115 | −0.0411 | 0.0081 | 0.0024 | −0.5712 | 0.1128 | 0.0330 |
| 6 N | −0.0535 | 0.0418 | −0.0007 | −0.0110 | 0.0086 | −0.0002 | −0.1534 | 0.1199 | −0.0021 |
| 7 C | 0.0439 | 0.0288 | 0.0021 | 0.0091 | 0.0059 | 0.0004 | 0.1260 | 0.0826 | 0.0059 |
| 8 C | −0.0524 | 0.0293 | 0.0078 | −0.0108 | 0.0060 | 0.0016 | −0.1504 | 0.0840 | 0.0224 |
| 9 C | −0.0566 | 0.0332 | 0.0171 | −0.0117 | 0.0069 | 0.0035 | −0.1624 | 0.0954 | 0.0491 |
| 10 C | 0.0802 | 0.0521 | 0.0111 | 0.0165 | 0.0107 | 0.0023 | 0.2301 | 0.1494 | 0.0319 |
| 11 C | −0.0530 | 0.0308 | 0.0159 | −0.0109 | 0.0064 | 0.0033 | −0.1521 | 0.0884 | 0.0457 |
| 12 C | −0.0455 | 0.0338 | 0.0150 | −0.0094 | 0.0070 | 0.0031 | −0.1306 | 0.0970 | 0.0432 |
| 13 C | 0.1820 | 0.0214 | 0.0020 | 0.0375 | 0.0044 | 0.0004 | 0.5221 | 0.0613 | 0.0058 |
| 14 C | −0.0272 | 0.0107 | 0.0022 | −0.0056 | 0.0022 | 0.0005 | −0.0782 | 0.0307 | 0.0064 |
| 15 C | −0.0495 | 0.0049 | 0.0025 | −0.0102 | 0.0010 | 0.0005 | −0.1422 | 0.0139 | 0.0071 |
| 16 C | −0.0588 | 0.0003 | 0.0019 | −0.0121 | 0.0001 | 0.0004 | −0.1686 | 0.0010 | 0.0055 |
| 17 C | 0.0486 | 0.0012 | 0.0012 | 0.0100 | 0.0003 | 0.0002 | 0.1395 | 0.0035 | 0.0034 |
| 18 C | −0.0140 | −0.0050 | 0.0076 | −0.0029 | −0.0010 | 0.0016 | −0.0401 | −0.0143 | 0.0217 |
| 19 C | −0.0433 | −0.0016 | 0.0160 | −0.0089 | −0.0003 | 0.0033 | −0.1244 | −0.0047 | 0.0460 |
| 20 C | −0.0625 | 0.0051 | 0.0259 | −0.0129 | 0.0011 | 0.0053 | −0.1794 | 0.0147 | 0.0742 |
| 21 C | 0.0834 | 0.0079 | 0.0155 | 0.0172 | 0.0016 | 0.0032 | 0.2394 | 0.0227 | 0.0446 |
| 22 C | −0.0539 | 0.0059 | 0.0187 | −0.0111 | 0.0012 | 0.0039 | −0.1545 | 0.0170 | 0.0537 |
| 23 C | −0.0301 | −0.0004 | 0.0210 | −0.0062 | −0.0001 | 0.0043 | −0.0865 | −0.0010 | 0.0602 |
| 24 C | 0.0307 | 0.0077 | 0.0013 | 0.0063 | 0.0016 | 0.0003 | 0.0882 | 0.0222 | 0.0037 |
| 25 C | −0.0305 | 0.0154 | 0.0032 | −0.0063 | 0.0032 | 0.0007 | −0.0876 | 0.0443 | 0.0092 |
| 26 C | −0.0410 | 0.0147 | 0.0175 | −0.0085 | 0.0030 | 0.0036 | −0.1177 | 0.0422 | 0.0503 |
| 27 C | −0.0571 | 0.0261 | 0.0205 | −0.0118 | 0.0054 | 0.0042 | −0.1637 | 0.0748 | 0.0587 |
| 28 C | 0.0737 | 0.0297 | 0.0121 | 0.0152 | 0.0061 | 0.0025 | 0.2114 | 0.0852 | 0.0348 |
| 29 C | −0.0647 | 0.0227 | 0.0230 | −0.0134 | 0.0047 | 0.0048 | −0.1857 | 0.0651 | 0.0661 |
| 30 C | −0.0391 | 0.0098 | 0.0156 | −0.0081 | 0.0020 | 0.0032 | −0.1122 | 0.0280 | 0.0446 |
| 31 H | 0.0455 | 0.0142 | 0.0054 | 0.0094 | 0.0029 | 0.0011 | 0.1307 | 0.0406 | 0.0154 |
| 32 H | 0.0560 | 0.0211 | 0.0103 | 0.0115 | 0.0043 | 0.0021 | 0.1606 | 0.0605 | 0.0295 |
| 33 H | 0.0576 | 0.0215 | 0.0100 | 0.0119 | 0.0044 | 0.0021 | 0.1654 | 0.0617 | 0.0287 |
| 34 H | 0.0510 | 0.0165 | 0.0071 | 0.0105 | 0.0034 | 0.0015 | 0.1462 | 0.0474 | 0.0203 |
| 35 H | 0.0497 | 0.0126 | 0.0048 | 0.0102 | 0.0026 | 0.0010 | 0.1425 | 0.0361 | 0.0138 |
| 36 H | 0.0337 | 0.0076 | 0.0024 | 0.0070 | 0.0016 | 0.0005 | 0.0968 | 0.0218 | 0.0070 |
| 37 H | 0.0461 | 0.0091 | 0.0043 | 0.0095 | 0.0019 | 0.0009 | 0.1323 | 0.0260 | 0.0123 |
| 38 H | 0.0260 | −0.0005 | 0.0024 | 0.0054 | −0.0001 | 0.0005 | 0.0747 | −0.0015 | 0.0068 |
| 39 H | 0.0393 | 0.0006 | 0.0020 | 0.0081 | 0.0001 | 0.0004 | 0.1126 | 0.0016 | 0.0057 |
| 40 H | 0.0281 | 0.0012 | 0.0029 | 0.0058 | 0.0002 | 0.0006 | 0.0806 | 0.0034 | 0.0084 |
| 41 H | 0.0450 | 0.0000 | 0.0130 | 0.0093 | 0.0000 | 0.0027 | 0.1292 | 0.0000 | 0.0372 |
| 42 H | 0.0531 | 0.0058 | 0.0326 | 0.0109 | 0.0012 | 0.0067 | 0.1523 | 0.0168 | 0.0934 |
| 43 H | 0.0562 | 0.0063 | 0.0161 | 0.0116 | 0.0013 | 0.0033 | 0.1613 | 0.0182 | 0.0462 |
| 44 H | 0.0542 | 0.0012 | 0.0129 | 0.0112 | 0.0002 | 0.0027 | 0.1554 | 0.0033 | 0.0371 |
| 45 H | 0.0379 | 0.0162 | 0.0026 | 0.0078 | 0.0033 | 0.0005 | 0.1088 | 0.0465 | 0.0076 |
| 46 H | 0.0449 | 0.0112 | 0.0096 | 0.0093 | 0.0023 | 0.0020 | 0.1288 | 0.0322 | 0.0277 |
| 47 H | 0.0527 | 0.0169 | 0.0117 | 0.0109 | 0.0035 | 0.0024 | 0.1512 | 0.0486 | 0.0334 |
| 48 H | 0.0455 | 0.0155 | 0.0137 | 0.0094 | 0.0032 | 0.0028 | 0.1306 | 0.0444 | 0.0392 |
| 49 H | 0.0482 | 0.0058 | 0.0081 | 0.0099 | 0.0012 | 0.0017 | 0.1384 | 0.0165 | 0.0231 |
| 50 H | 0.1138 | 0.0039 | 0.0029 | 0.0235 | 0.0008 | 0.0006 | 0.3266 | 0.0112 | 0.0084 |
| 51 H | 0.1765 | 0.0168 | 0.0082 | 0.0364 | 0.0035 | 0.0017 | 0.5065 | 0.0481 | 0.0235 |
| 52 O | −0.2883 | 0.0234 | 0.0082 | −0.0594 | 0.0048 | 0.0017 | −0.8271 | 0.0673 | 0.0235 |
| 53 O | −0.3467 | 0.0875 | 0.0182 | −0.0715 | 0.0180 | 0.0037 | −0.9949 | 0.2509 | 0.0522 |
| 54 N | 0.0001 | 0.0042 | 0.0026 | 0.0000 | 0.0009 | 0.0005 | 0.0004 | 0.0122 | 0.0073 |
| 55 C | 0.1635 | 0.0235 | 0.0043 | 0.0337 | 0.0048 | 0.0009 | 0.4692 | 0.0675 | 0.0124 |
| 56 C | 0.0296 | 0.0097 | 0.0112 | 0.0061 | 0.0020 | 0.0023 | 0.0850 | 0.0278 | 0.0322 |
| 57 C | −0.0507 | 0.0047 | 0.0238 | −0.0105 | 0.0010 | 0.0049 | −0.1455 | 0.0136 | 0.0682 |
| 58 C | −0.0496 | 0.0043 | 0.0254 | −0.0102 | 0.0009 | 0.0052 | −0.1424 | 0.0124 | 0.0728 |
| 59 C | 0.0198 | 0.0046 | 0.0398 | 0.0041 | 0.0009 | 0.0082 | 0.0567 | 0.0131 | 0.1143 |
| 60 H | 0.1664 | 0.0037 | 0.0157 | 0.0343 | 0.0008 | 0.0032 | 0.4775 | 0.0106 | 0.0451 |
| 61 H | 0.1142 | 0.0035 | 0.0052 | 0.0235 | 0.0007 | 0.0011 | 0.3277 | 0.0100 | 0.0150 |
| 62 H | 0.0545 | 0.0063 | 0.0252 | 0.0112 | 0.0013 | 0.0052 | 0.1564 | 0.0182 | 0.0724 |
| 63 H | 0.0556 | 0.0046 | 0.0168 | 0.0115 | 0.0009 | 0.0035 | 0.1594 | 0.0131 | 0.0482 |
| 64 H | 0.0359 | 0.0089 | 0.0657 | 0.0074 | 0.0018 | 0.0136 | 0.1029 | 0.0255 | 0.1886 |
| 65 H | 0.0444 | 0.0025 | 0.0162 | 0.0091 | 0.0005 | 0.0033 | 0.1273 | 0.0071 | 0.0465 |
| 66 H | 0.0496 | 0.0089 | 0.0622 | 0.0102 | 0.0018 | 0.0128 | 0.1423 | 0.0255 | 0.1785 |
| 67 H | 0.0394 | 0.0056 | 0.0376 | 0.0081 | 0.0012 | 0.0077 | 0.1131 | 0.0162 | 0.1078 |
| 68 H | 0.0475 | 0.0060 | 0.0967 | 0.0098 | 0.0012 | 0.0199 | 0.1364 | 0.0173 | 0.2775 |
